# New Solids in As-O-Mo, As(P)-O-Mo(W) and As(P)-O-Nb(W) Systems That Exhibit Nonlinear Optical Properties

**DOI:** 10.3390/molecules26051494

**Published:** 2021-03-09

**Authors:** Nikolay Gerasimchuk, Lauri Kivijarvi, Bruce Noll, Meriem Goudjil, Soma Khanra, Yu Ping, Miles Pearson, Frank Röminger

**Affiliations:** 1 Department of Chemistry, Missouri State University, Temple Hall 456, Springfield, MO 65897, USA; LauriKivijarvi@MissouriState.edu (L.K.); Miles27@live.missouristate.edu (M.P.); 2 Bruker AXS, Inc., Madison, WI 53722, USA; Bruce.Noll@bruker-axs.com; 3 Laboratoire des Sciences des Matériaux, Faculté de Chimie, Université des Sciences et de la Technologie Houari Boumediène, Alger 16111, Algeria; bonus.chudo@gmail.com; 4 Department of Physics and Astronomy, 220 Physics Building University of Missouri Columbia, Columbia, MO 65211, USA; sk9n6@mail.missouri.edu (S.K.); yuping@missouri.edu (Y.P.); 5 Organisch-Chemisches Institut der Universität Heidelberg Lehrstuhl für Organische Chemie III 6 Im Neuenheimer Feld 270, Heidelberg University, D-69120 Heidelberg, Germany; frank.rominger@oci.uni-heidelberg.de

**Keywords:** oxide bronzes, mixed valence, crystal structures, EPR spectra, band gaps, second-harmonic-generation, single crystal electrical conductivity

## Abstract

Interactions between well-mixed fine powders of As_2_O_3_, P_2_O_5_, MoO_3_, WO_3_ and Nb_2_O_5_ at different stoichiometry in quartz ampoules under vacuum at ~1000 °C in the presence of metallic molybdenum (or niobium), over several weeks, led to shiny dichroic crystalline materials being formed in cooler parts of the reaction vessel. An addition of small quantities of metals-Mo or Nb-was made with the aim of partially reducing their highly oxidized Mo(VI), W(VI) or Nb(V) species to corresponding Mo(V), W(V) and Nb(IV) centers, in order to form mixed valence solids. Sublimed crystals of four new compounds were investigated using a variety of techniques, with prime emphasis on the X-ray analysis, followed by spectroscopy (diffusion reflectance, IR, Raman and EPR), second harmonic generation (SHG), thermal analysis under N_2_ and air atmosphere, and single crystals electrical conductivity studies. The results evidenced the formation of new complex solids of previously unknown compositions and structures. Three out of four compounds crystallized in non-centrosymmetric space groups and represent layered 2D polymeric puckered structures that being stacked on each other form 3D lattices. All new solids exhibit strong second-harmonic-generation (SHG effect; based on YAG 1064 nm tests with detection of 532 nm photons), and a rare ***photosalient effect*** when crystals physically move in the laser beam. Single crystals’ electrical conductivity of the four new synthesized compounds was measured, and the results showed their semiconductor behavior. Values of band gaps of these new solids were determined using diffusion reflectance spectroscopy in the visible region. Aspects of new solids’ practical usefulness are discussed.

## 1. Introduction

In the late ’60 and early ’70 new types of seemingly simple inorganic compounds, such as stoichiometric niobates and titanates, quickly emerged as ferroelectic, piezoelectric and light modulating materials [1,2,3]. The latter property was found to be especially valuable for practical uses and now is widely used in mobile telephones and doubling laser frequency applications in military and industry [4,5,6]. The modulation of light is the key characteristic of non-linear optical materials (NLOMs). The origin of such interesting and useful property is crystallization of the above inorganic solids in acentric and polar space groups due to intrinsic distortions of the transition metal-oxygen MO_6_ environment. These distortions initiate from rather considerable mismatch in sizes of highly oxidized small transition metals centers compared with O^2−^ ions forming MO_6_ octahedral “cages”. As the result, oxidized small metals ions of Ti^4+^, Zr^4+^, Nb^5+^, Ta^5+^ are loosely bound to some of the surrounding oxygen ions causing the spread of M-O distances over a large range, often being up to 10–30% of the averaged value. Thus, in these structures the transition metal is also displaced off the center of equatorial plane in MO_6_ octahedrons, which leads to intrinsic polarization of the crystal [7]. Geometrical distortions are causing some tilting of coordination polyhedrons and their twisting with respect to each other that also contributes to origination of spontaneous polarization in the crystal. In the past all research in the field of these new materials was focused on a rapidly grown variety of stoichiometric phases including their doping with other transition metal ions. Resilience to photons flux and stability at high temperatures of these compounds and phases was the second most practically valuable property.

Until recent years Mo and W oxides were not part of the effort mainly because of their propensity to form non-stoichiometric oxides and salts due to easy attainable less positive oxidation states +5 and +4. As the result of such reduction, often in poorly controlled process, mixed valence phases of different composition typically were formed. Nevertheless, mixed valence solids, such as oxides based on molybdenum blues and tungsten bronzes, opened a new era in inorganic and then materials chemistry after the pioneering work of Arne Magneli [8,9]. His discoveries of unusual structures and the mixed valence nature of these new phases inspired explosive growth of interest to these new oxides-based phases [10,11,12,13,14]. These new non-stoichiometric systems turned out to be primarily of great importance in electronics as conducting/semiconducting materials [15,16,17,18,19,20,21,22] and quite recently in catalysis [23,24,25,26,27,28], and nano-technology [29,30,31]. One of the most exciting applications of a variety of metal oxides is area of non-linear optical materials [32,33,34,35,36]. In this new field of practical importance, the design and preparation of thermally robust NLOMs, based on noncentrosymmetric inorganic compounds and phases, is critical. The visionary ideas and work of Halasayamani and his co-workers to introduce elements in oxidation states with lone pairs, which may provide thought distortion in formed crystal lattices, turned out to be fruitful [32]. Thus, elements such as Te, Se, Pb, Sb and Bi have been successfully used for making noncentrosymmetric solids based on molybdates and tungstates that demonstrate the second harmonic generation effect (SHG) [37,38,39,40,41,42]. It should be especially noted that it is not conventional doping process since these main group elements are not isomorphous substitution of transition metal ions in the structure, but actually get incorporated in the crystal lattice and in that way modify it. As said earlier, acentric materials also possess to various degree a spectrum of other useful properties, such as piezoelectricity, pyroelectricity and ferroelecticity [43]. Another interesting new area of potential application of novel mixed transition metals oxides was recently found as non-metallic materials for electrodes in fuel cells [44].

Despite some considerable efforts in the past, many of these interesting systems were not sufficiently investigated. We continued studies in this area of solid-state chemistry and prepared several new *homometallic* (Mo) and *heterometallic* (Mo/W, Nb/W) systems also containing pnictogens P and As as the lattice modifying elements. To the best of our knowledge, both elements have not been previously successfully used for this purpose. With this approach we intended to achieve two goals: (a) to prepare new mixed valence oxide-based bronzes that will possess electrical conductivity due to presence of charge carriers, and (b) to synthesize acentric solids that may demonstrate NLOM properties. In this work we report our findings of isolation of four new compounds, which underwent subsequent investigation, and their evaluation of possibility for their practical applications.

## 2. Results and Discussion

### 2.1. Preparation of Compounds

**Figure 1:**
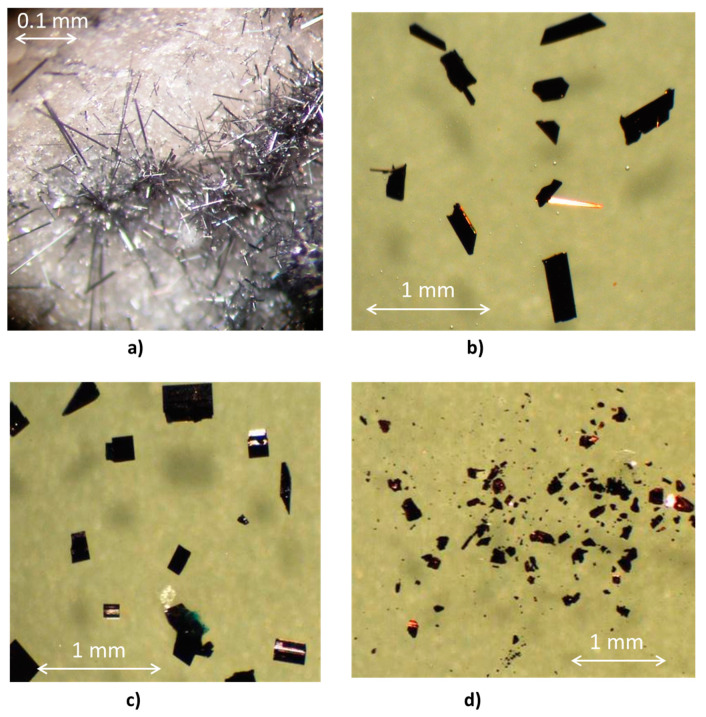
Actual microscope photographs (40×) of crystalline compound **1** in the opened quartz tube (**a**), and crystals of extracted from ampoules compounds **2**–**4** in an immersion oil for selection for the XRD studies (**b**–**d**).

The approach of thoroughly heating mixed powders of metal oxides and pnictogen oxides led to the successful obtaining of new solid-state phases in quartz ampoules (Supplementary Materials, Figure S1). These new solids represent oxide bronzes containing mixed valence *homometallic* and, as the result of isomorphous substitution, *heterometallic* solid solutions of suboxides category [45]. The introduction of As(III) atoms with stereo-active lone pair indeed helped in creating distortion in the crystal lattice that shaped rather unusual new structures. Synthesized compounds possess an attractive shape and color, being shiny luster crystalline materials (Figure 1), and being heat stable, demonstrate useful properties of non-linear optical systems. We have to mention that the preparation of this type of compounds is not optimized at this stage, since the yields of sublimed crystalline materials were generally lower than expected, and, after the opening of the ampoules, we also observed well-defined crystals of the initial As_2_O_3,_ as well as MoO_3_, and monoclinic and orthorhombic polymorphs of Mo_4_O_11_ which were initially not present in the reaction mixture.

The main objective of the current synthetic approach was to prove a concept of making non-centrosymmetric solids based on non-metal oxides which possess lone pair, and that objective was largely achieved. Formulas of all four new compounds prepared in this study, with abbreviations for their proper citing, are presented in Table 1. Full discussion of their structures and composition is given later in the text.

### 2.2. Second Harmonic Generation (SHG)

**Figure 2:**
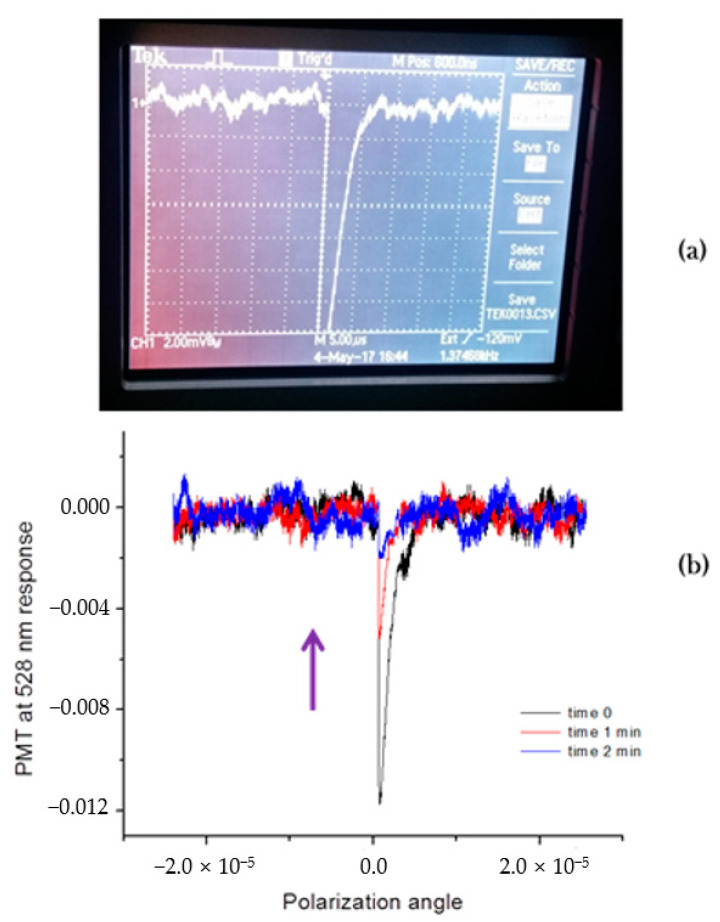
Investigation of interaction of the YAG beam with single crystals of mixed metal oxide solids: oscilloscope screen shot from irradiated specimen of compound **1** showing strong, even truncated signal SHG effect at 290 K (**a**), and intensity decay of the SHG signal at 532 nm for a crystal of compound **3** (**b**).

**Figure 3:**
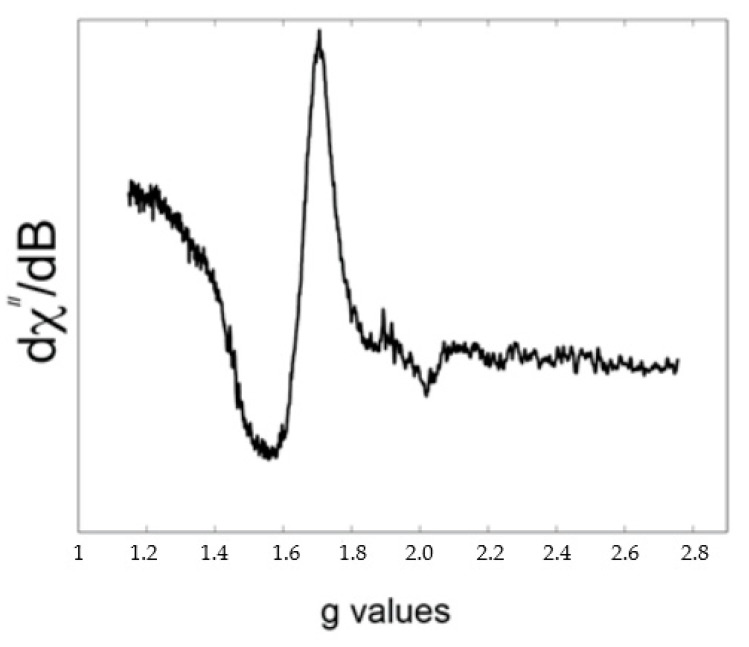
EPR spectrum of grey-blue needle of compound **1** at 80 K showing signal of Nb(IV) centers.

The generation of photons of double energy (twice the smaller wavelength) from the carrier laser source is a manifestation of a non-linear optical phenomenon associated with crystals that do not possess a center of symmetry. We used the Yttrium-Aluminum-Garnet (YAG) laser, with 1064 nm radiation, with a short bandwidth filter, allowing the detection of only photons with 532 nm wavelength in our experiments (SM Figures S2 and S3). Optical alignment of small single crystals of studied compounds **1**–**4** proved a tedious procedure. A qualitative examination of the crystals in the presence of the SHG effect was carried out, using the appearance/presence of a signal on an oscilloscope. It turned out that three out of four of the samples investigated (compounds **1**, **3** and **4**) showed the SHG effect. This fact was immediately very helpful in the selection of proper (non-centrosymmetric) space groups for successful crystal structures solution and refinement (Figure 2a). We were able to record, partially, its circular polarization profile (Figure 2b; SM Figures S4 and S5) for single crystals of compound **1**. However, we observed unusual SHG signal behavior in samples of **1**, **3** and **4**—its intensity decreased with time of exposure to the YAG laser beam (Figure 3). Numerous repetitions and careful observations evidenced the physical movement of crystals away from the laser beam. This new phenomenon was originally observed in crystals of other inorganic compounds (Werner-type complexes). This was called the ***photosalient effect*** [46,47,48] and was categorized based on a type of crystals’ motion as displayed in SM 4 Chart S1. Our studied crystals exhibited mode 4, which is simple motion in one direction. Initially, we thought about crystals’ response to oxidation of As(III), Mo(V), W(V) species in an intense laser beam, in the open air. Placement of crystals into flat capillaries made of borosilicate glass from 5 mm NMR tubes under vacuum did not alleviate the effect: crystals still were moving away from the laser beam. Our compounds are all dark colored and absorb visible light. Thus, rapid heat delivered by the laser beam creates uneven crystal expansion which leads to the formation of reactive force propelling it away from the heat source. Indeed, studies of temperature variations of the unit cell constants evidence unidirectional rapid expansion for compound **4**, for example (SM 5, Figure S6). This is the explanation for the “type four” ***photosalient effect*** observed in studied new mixed metal oxides. Thus, unit cell constants *b* and *c* in this orthorhombic crystal do not change much with temperature, while the shortest dimension *a* does change considerably. This is the distance between layers, or puckered plates, in the structure (see discussion below), which turned out to be sensitive to temperature changes.

### 2.3. EPR Spectra

The electron spin resonance technique (ESP, or EPR) has been used to confirm the presence of less positive oxidation states of metal centers in the new solids obtained due to the presence of an unpaired electron. Reduction of metal centers during chemical reaction in the initial diamagnetic oxides (N_2_O_5_, MoO_3_ or WO_3_) by powders of the same, or different metals, leads to the formation of paramagnetic centers available for detection by the EPR method. Principal spectroscopic features for studied transition metals are summarized in SM 6, Table S1. It should be noted that the EPR spectra of compounds **1**–**4** are all different. Thus, in the case of the compound **1,** a broad signal with *g* = 1.643, which is typical for coupled isotropic Nb(IV) ions, was recorded (Figure 3). The observed spectrum is in line with other data for magnetically coupled Nb(IV) centers in the EPR spectra [49,50,51].

**Figure 4:**
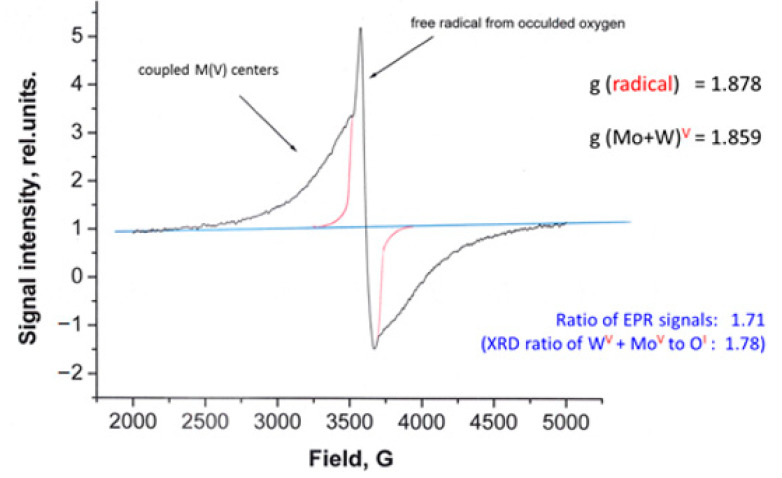
The EPR spectrum of compound **2** at 80 K (black trace). The radical component in spectroscopic envelope (red), signal from coupled pentavalent metals, and the baseline (blue), accompanied with their g-factors.

The EPR spectrum of compound **2** unexpectedly showed two signals (Figure 4): one sharp and narrow, the other featureless and broad. The sharp singlet clearly evidenced the presence of the free radical, and when a crystallographic solution of compound **2** became available, it was confirmed to be an oxygen atom, trapped inside the crystal lattice (Figures 8 and 9). The second broad signal belongs to the reduced Mo(V) and W(V) species, which undergo magnetic coupling due to their close proximity in the crystal lattice. This coupling was previously established for several mixed valence Mo-oxides in magnetochemical studies [52]. Deconvolution of the EPR spectrum finds the ratio between the two signals to be 1.71 (Figure 4). Interestingly, the same ratio, between the refined site occupancy factors of O centers and the sum of Mo(V) and W(V) centers obtained from the crystal structure of **2,** was found to be 1.78, which is within 4% error.

The EPR spectrum of compound **3** consists of a broad signal of overlapped and coupled Mo(V) and W(V) centers (SM 7, Figure S7). Compound **4** exhibits two different sites in its EPR spectrum (SM 8, Figure S8) that correspond to two crystallographically different Mo^V^ centers (confirmed by the X-ray analysis: see discussion later on). The spectrum has rhombic symmetry, and all three g-factors were determined as being in the range of 1.994 to 2.040 (SM 8, Figure S8). The presence of broad signals in the EPR spectra of **1**–**3** clearly evidenced coupling between reduced metal centers due to their close spatial proximity in crystal lattices of these compounds [53].

The most remarkable feature of EPR spectroscopy of all the studied compounds, **1**–**4**, is that signals of unpaired electrons on metal centers disappeared when samples underwent heating during thermal analysis TG/DSC studies in a pure nitrogen atmosphere, when it was used as a gas carrier (SM 9, Figure S9). This fact can be attributed to the reduction of nitrogen by pentavalent (Mo, W), or tetravalent Nb centers. Interestingly, a similar process takes place in nature during the nitrogen fixation on bacteria living on the roots of some plants [54] where gaseous N_2_ is reduced to hydrazine/hydroxylamine. Transition metals used in this work easily undergo reduction (SM 10, Table S2).

### 2.4. Thermal Analysis

Compounds **3** and **4** after anaerobic heating under Ar generated nice shiny bronze-color prisms that turned out to be monoclinic MoO_2_. Traces of the weight loss for other studied compounds (**1** and **2**) showed evolution of oxygen upon heating and formation of more complex non-identified products along their decomposition path.

### 2.5. Vibrational Spectra

Both IR- and micro-Raman spectra were recorded from crystalline samples of **1**–**4** solely for the purpose of confirmation of the presence of [NbO_6_], [MoO_6_], [WO_6_] species. Spectra were also recorded for starting compounds As_2_O_3_, P_2_O_5_, Nb_2_O_5_, MoO_3_ and WO_3_ for comparison with the obtained new phases. Data for compounds **1**–**4** presented in Table 2 with actual Raman spectra of compounds presented in SM 11, 12; Figures S10–S13. Observed vibrations in spectra of new compounds match well with those reported in earlier classic data by Wachs and co-workers for Nb-O [55,56], W-O [57] and Mo-O [58].

More specifically, in vibrational spectra of compound **1** we can assign NbO_6_ vibrations at 149 cm^−1^ to octahedra as a whole [59], and higher frequency stretching Nb-O vibrations at 284 cm^−1^ to one that contains reduced Nb^4+^ centers [60]. (Table 2; SM 11, Figure S10). The major Raman band at 936 cm^−1^ well corresponds to that found in other Nb-based layered oxides [56] which agrees with the range of 910–920 cm^−1^ bands assigned to Nb=O vibrations reported in the literature [37]. Moreover, the absence of a major Raman band at ~620–630 cm^−1^ indicates that compound **1** has no perovskite structure, which was confirmed by XRD studies, which will be discussed later. At the same time, we have not observed Nb-O vibrations in 580–590 cm^−1^ region [37] in our spectra (SM 11, Figure S10). We assigned vibrations at 850 and 838 cm^−1^ to the asymmetric and symmetric stretching mode of the Nb-O-Nb linkage (Table 2) [56,61]. A rich and complex spectroscopic envelope in the Raman spectra of **1** in 700–1000 cm^−1^ range contains also multiple W-O and W-O-W, W=O vibrations [62,63,64]. We were unable to identify other expected, but lower intensity, bands, corresponding to vibrations of As-O and P-O fragments in both Raman and IR-spectra of **1,** due to their overlap with the above more intense and abundant M-O (M = Nb, W) vibrations.

In vibrational spectra of compound **2** we were able to identify Mo=O, W=O (at 978 cm^−1^), Mo-O-Mo (985 cm^−1^), W-O-W (at ~790 cm^−1^) and Mo-O (984 cm^−1^), W-O (~443 cm^−1^) vibrations (Table 2; SM 11, Figure S11) according to literature assignments [57]. The phosphate group is manifested as two intense bands at 1090 and 1061 cm^−1^.

The IR- and Raman spectra of compound **3** and **4** are quite similar (SM 12, Figures S12 and S13; Table S2) which implies correspondence in structure which was confirmed by the X-ray analysis (see discussion below). We assigned a Raman band at 983 cm^−1^ to terminal Mo-O vibration, while bands at 827 and 833 cm^−1^ were thought to be bridging O-Mo-O vibrations [65]. We did not observe the expected vibrations at 109, 215, 322, 550, 602 and 685 cm^−1^ for As(III)-O moieties [66] in the Raman spectrum, most likely because of much lower As(III) content in the structures of **3** and **4,** leading to small intensities of those peaks that were overshadowed with more abundant and intense Mo-O vibrations.

### 2.6. Electronic Spectra

**Figure 5:**
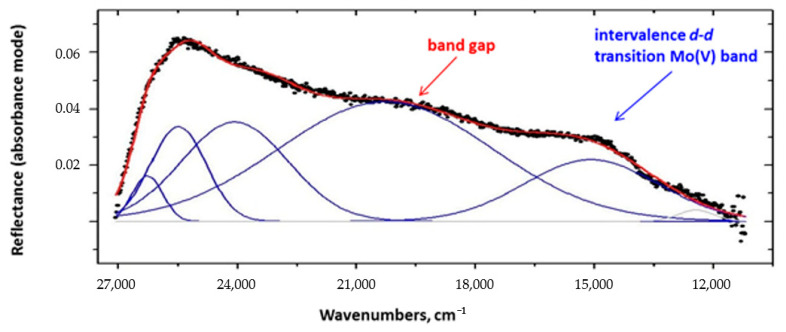
Electronic diffuse reflectance spectrum of fine powder of compound **2** (black dots) and its full line shape analysis (blue traces) and the best fit to data (red trace).

UV-visible spectra from ground crystals of compounds **1**–**4** were recorded in solid-state (diffusion reflectance mode, DRS) to observe *d-d* transitions characteristic for lower oxidation states of transition metals. Thus, Nb(IV), Mo(V) and W(V) species possess blue color, and their presence confirms the formation of sites with reduced metal centers, and, therefore, the formation of mixed valence species. This intervalence charge transfer band is typically present for Mo(V) species in the range of 13,000–16,000 cm^−1^ [67,68]. The transition metals used in this study have fairly low redox potentials (SM 10, Table S2), and technically there was a possibility of the reduction of transition metals to a less positive state 4+ (Mo, W) or 3+ (Nb), but in the experimental conditions used, their formation is unlikely. Recorded electronic spectra were used to calculate the band gap in compounds **1**–**4**. A typical example of reflectance spectra of compound **4,** and its deconvolution, is present in Figure 5, where an assignment for an intervalence charge transfer band and the lowest transition used for the band gap calculation is shown as well. Details of the line-shape analysis can be found in SM 13, Figure S14. The band gaps are calculated from the electronic spectra as follows: for **2** = 2.51 eV, for **3** = 3.37 eV and for **4** = 2.29 eV. The obtained values are in good agreement with data of single crystals electrical conductivity for these compounds: higher conductance corresponds to a lower band gap in these, which appear to be semiconducting solids (Table 4, see below Section 2.7.6: Single crystal conductivity.).

### 2.7. Structures of Mixed Metal Oxides

#### 2.7.1. General Considerations

Prior to initiation of this work we realized that structural studies of mixed valent metal oxides represent great challenge because of the following reasons: mixed valence of metal sites in *homometallic* systems that generates issues with charge balance, presence of isomorphously substituted *heterometallic* centers creating solid solutions, difficulties with proper X-ray absorption correction in formed dense, heavy metal oxides acting as “beam stoppers”, and lastly, strong propensity for twinning and formation of multi-domain species. Nevertheless, despite these obstacles we attempted preparation of these new solid phases to evaluate possibility for a formation of new structures and related to its properties. Below we present a brief summary of terms used in the ensued discussion.

Glossary for used in this portion of discussion abbreviations:

ASU = asymmetric unit of the crystal structure;

GOF = goodness of fit of data to the model;

EXYZ = instruction (card) used in treatment of disorder [positional or substitutional] where atoms of interest are assigned the same place in the lattice;

EADP = instruction (card) used in treatment of disorder with poorly defined thermal parameters of one of the components;

SOF = site occupancy factor with 11 being for an atom in general position with fully occupied site;

RES = refined structure output file containing important numerical values of the structure;

INS = instruction file necessary for the structure refinement;

NPD = non-positive definite—indication that the amount and shape of electron density is not sufficient for identifying an atom in that specific place in the lattice;

RLAT = reciprocal lattice viewer represents a software tool allowing to visualize in color selected (or all) reflections presented in reciprocal lattice proved to be very useful for analyzing multicomponent, twinned specimen and modulated structures;

BASF = batch scaling factor—the number indicating the fraction of one component of the lattice with respect to the other; has a usable range from 0.95 to 0.05;

ORTEP = stands for the program development site “Oak-Ridge Thermal Ellipsoids Parameters” and represents plotting of atoms according to magnitude of their thermal motion parameters; conventionally is chosen to be at 50% probability level.

SADABS = program that allows accurate determination of a crystal absorption using the least-squares procedure for modeling an empirical transmission surface as sampled by multiple symmetry-equivalent and/or azimuth rotation-equivalent intensity measurements.

GROW = command used by software to use symmetry operations for a give space group to generate view of the unit cell content from the ASU.

PLATON = powerful software package often used for the verification of space group, search for missed/additional symmetry: https://www.platonsoft.nl/platon/ (accessed on 27 January 2021).

Before attempting a successful crystal structures solution and refinement, additional auxiliary information must be collected and processed, as presented above. Thus, the formation of non-centrosymmetric crystal lattices was established using SHG experiments. The EPR and DRS electronic spectroscopy confirmed the presence of reduced metal centers Nb(IV) in **1**, Mo(V) and W(V) in **2**–**4**. The latter was necessary for the determination of actual composition of new solids, and to assure proper charge balance in crystal lattices. In most cases, these reduced oxidation states metal centers were statistically distributed throughout the crystal. At the initial stages of refinement, a *homometallic* mode was applied, that is, all Nb, Mo, W atoms only in the structure. Residual electron density which was either too high, or insufficient, indicated the necessity of *heterometallic* refinement which is typical of isomorphous substitution in minerals and in solid solutions. Thus, it was necessary to place two different metal ions in the same site. We inserted EXYZ, EADP cards into the INS files, tying total site occupancies to *1*, and introducing an extra free-variable parameter. This procedure has to be done for all sites where isomorphous substitution of different metals could take place. Criteria for a successful modeling of the structure were the lowest values of R1 (wR2), GOF, residual electron density and atoms shifts when refinement converged. However, for sites with different oxidation states of the *same metal*, tedious analysis of oxygen atoms site occupancy factors (SOFs) surrounding a metal center has to be performed, at one site at a time, to assure stable refinement and avoid structure collapse. Analysis of SOFs carried out suggested the most probable place for the reduced transition metals centers. The presence of both mixed oxidation states and different transition metals in the same structure, created a situation of solid solutions, and gave us unrepresented challenge in the determination of crystal structures of new solids. On top of the issues outlined here all the crystalline specimens selected for studies, although regularly shaped and appearing beautiful externally, turned out to be of poor intrinsic quality with mozaicity being greater than 0.6°.

In all the structures presented herein, the environments of transition metal centers are significantly distorted, similarly to their parenting metal oxides MoO_3_, WO_3_ and Nb_2_O_5_ [32,69]. Thus, our observations are in line with numerous previous reports on highly distorted O6 octahedrons of Mo(V/VI), W(V/VI) and Nb(IV/V). These distortions originate from rather considerable mismatch in sizes of highly oxidized small in size transition metals centers compared with O^2−^ ions forming MO_6_ octahedron “cages” to hold the former. As the result, Mo(V/VI), W(V/VI) and Nb(IV/V) are shifting from the center of octahedrons, causing the spread of M-O distances over a large range, often being up to 20–30% of the averaged value. Moreover, very often, the transition metal center is off the plane in MO_6_ octahedrons [70]. These distortions lead to the tilt between corner- or edge-sharing MO_6_ octahedrons in crystal structures. Tilt angles between those are varied over the range of 5 to 30°. There is twisting of MO_6_ units with respect to each other, leading to angles between faces of adjacent octahedrons different from 90°. Moreover, it should be noted that the fraction of pnictogens in compounds **1**–**4** is small, as compared with transition metals Mo, W or Nb. Presence of As(III) atoms in structures of **3** and **4**, with the vast majority of atoms being Mo, does not affect the geometry of the metal center micro environments. However, the lone pair As atoms play a critical role, introducing further distortion in the lattice and being responsible for the adoption of non-centrosymmetric structures of **3** and **4**.

Below, we report crystal structures of compounds **1**–**4** in their final stage of refinement accounting for all the above particularities. In two cases there were successfully localized and refined different metal sites, but in two cases it was possible to establish structural motif and atoms connectivity. In latter cases, the best crystals selected for studies were multi-domain or modulated specimens that were difficult to model and refine. It should be specially noticed that all the presented structures are different from those for various molybdenum or tungsten mixed valence oxides, for which a special term ‘suboxides’ was created early on [45]. An expected peculiarity of structures of studied compounds was their high absorption coefficient. While the exact positions of heavy atoms have been unambiguously established, places for oxygen atoms have to be carefully refined. Poor correction leads to overly enlarged (or the opposite–flattened, “pancake”) thermal ellipsoids of oxygen atoms, which frequently led to the NPD status of some atoms. The best way for the determination of µ is when crystals face indexing from a series of photographs using the videomicroscope following the SADABS program [71]. However, due to the crystals’ small size in many cases, and their difficult habitus, such as thin plates, for instance, videoscaling was not applicable and a multi-scan method was used. Details of crystal and refinement data are presented in Table 3, while selected bond lengths and angles for structures of **1**–**4** can be found in multiple pages of Supporting Materials.

#### 2.7.2. Structure of **1**

**Figure 6:**
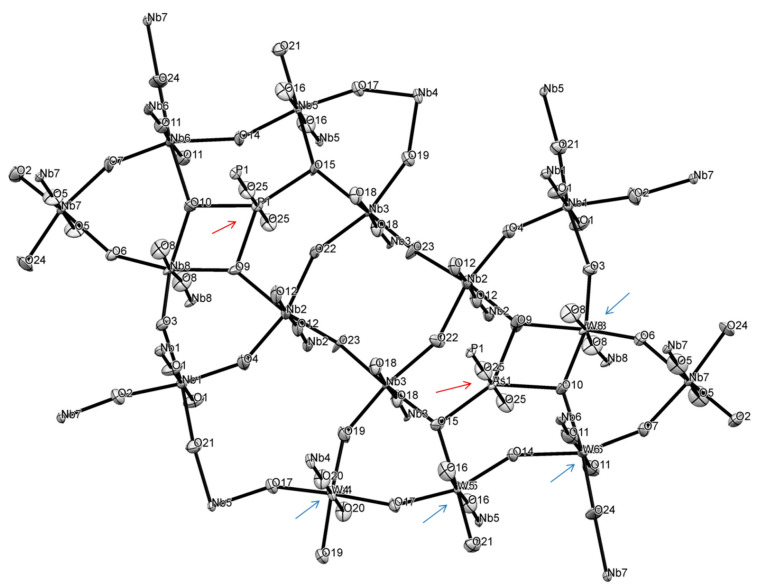
The ASU in the crystal structure of compound **1** in an ORTEP representation drawn at 50% thermal ellipsoids probability level. Shared between transition metals sites are indicated by blue arrows, while those for phictogens are shown by red arrows.

**Figure 7:**
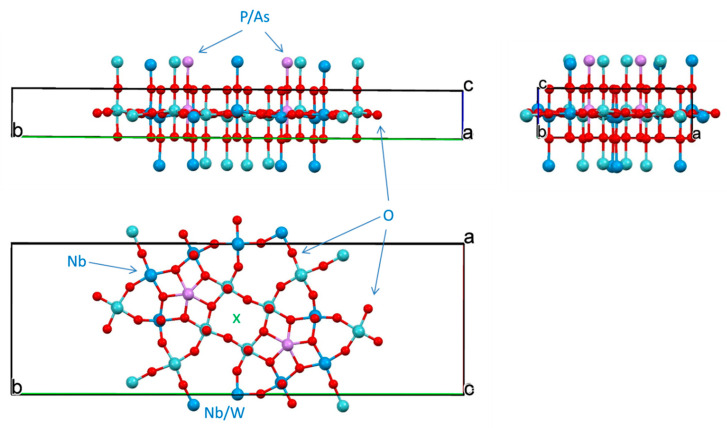
Three orthogonal projections of the ASU in the unit cell in the crystal structure of compound **1**: top—view along *a*, bottom left—along *c*, and right—view along *b* direction. The 2-fold rotational axis is marked as green X.

Because of the very small dimensions (microns-size crystal of a needle habitus) specimen selected for structural determination, faces indexing could not be used for the absorption correction procedure, and a multi-scan method was used [72]. Compound **1** formed incommensurate structure. An attempt at regular data collection, processing and structure solution was unsuccessful for several selected specimens. Analysis of precession images (SM 14, Figures S15 and S16) evidenced the presence of modulation, which was clearly observed as three clusters of reflections in the reciprocal lattice viewer (RLAT) (SM 15, Figures S17 and S18). For the structure solution we selected only one, the biggest domain. Compound **1** crystallizes in a non-centrosymmetric space group, as was evidenced by its rather strong SHG effect. Out of several plausible choices, the P2_1_2_1_2 space group was selected and led to a successful structure solution (Table 3). Nevertheless, the crystal specimen was twinned and refinement converged when the model was used as an inversion twin (BASF = 0.427). The structure incorporates both P and As atoms (as expected from the synthesis), reduced Nb(IV) and W(V) centers. The asymmetric unit of the structure and labeling scheme is shown in Figure 6 with the unit cell content displayed in Figure 7, and polyhedral representation of the structure of compound **1** is depicted in SM 16, 17. Thus, final refinement converged for this structure of Nb/W oxide bronze of the “P_4_Nb_30_O_85_” type with chemical formula (P/As)_4_(Nb/W)_30_O_83.08_, which indicates the presence of Nb(IV) and W(V) centers to balance charge. Because of the much easier reduction of W(VI) to W(V) we assumed that it is all the pentavalent state in this compound. It is not possible, however, to identify sites for reduced metal cations as well as relationship between oxidized and reduced species for transition metals. The bond lengths Nb-O in the structure of **1** are in the range of 1.783 to 2.165 Å on the same vertex in distorted NbO_6_ octahedrons with O-Nb-O angles there less than 180° (SM 16, 17). This is rather similar to those in earlier studied niobium oxides [73,74,75]. Because of sharing of the transition metal site bonds W-O bonds have the same range and are also very similar to numerous to previously found in tungsten oxides [76,77]. The situation with O-M-O angles for both metals is analogous.

The ASU of this compound has interesting features, such as pentagonal star shape units containing pnictogen atoms inside and 4-, 6- and 8-membered cyclic fragments (Figure 7) all interconnected with bridging oxygen atoms. Thus, in the ASU there are 16 oxygen atoms between two transition metals (Nb and/or W) with 4 atoms O3, O4, O6 and O7 being unique, while the rest are multiplied through C2 axis and two-fold screw axis in this space group. There are 5 atoms, all in the star fragment, that act as bridges between 3 transition metals: O9, O10, O14, O15 and O22. Thus, in the pentagonal star Nb2-O22-Nb3-O15-Nb5(W5)-O14-Nb6(W6)-O10-Nb8(W8), which represents ten-membered metallo-cycle, there are five practically planar [MO_2_Pn] rhombs (Pn = pnictogen atoms: P and or As). These pnictogens were refined to have 0.512 (for P1) and 0.488 (for As1) site occupancies. There are 4 niobium atoms in the structure (Nb1, Nb2, Nb3 and Nb7) that fully occupy positions with SOF = 1, while Nb4, Nb5, Nb6 and Nb8 share their sites with tungsten atoms W4, W5, W6 and W7. The latter have the following SOFs: W4 = 0.183, W5 = 0.122, W6 = 0.020 and W8 = 0.325 with SOFs of their Nb counterparts (Nb4 = 0.817, Nb5 = 0.882, Nb6 = 0.980 and Nb8 = 0.676) being added together equal to one for each site. The SOFs for oxygen atoms in the core of the structure O9, O10, O14, O15 and O22 that are bridged between 3 metal centers were not refined. The SOFs for the rest of the oxygen sites were refined.

The central 8-membered cycle is not planar, adopts a boat-shape and has 37.20° dihedral angle between two Nb3-O22-Nb2 planes (SM 18, Figure S21). All three 6-membered rings in the structure are non-planar with values of dihedral angles being 34.57° (with Nb3), 20.86° (with Nb7) and 25.24° (with Nb2) (SM 19, Figure S22). The pentagonal star shape is formed by 3 in-plane shorter bonds and 2 longer bonds with 2 additional axial bonds (SM 20, Figure S23). This fragment adopts a bowl structure where the distance between the mean plane of transition metals and P1/As1 center is 0.423 Å, while between the mean plane of 5 oxygen atoms and pnictogens is 0.223 Å. Both As and P atoms are in +5 oxidation state. Details of geometry of such unusual formation are shown in SM 21, Figure S24. The environment of As1/P1 atoms can be well approximated as a distorted pentagonal bipyramid, where As1/P1 distances to O14 (2.195 Å) and to O22 (2.193 Å) are ~0.15 Å longer than their bonds to O9, O10 and O15 (SM 22, Figure S25).

Both individual and averaged Nb-O distances appeared to be in line with the same observed for other oxides of this metal (SM 23, Figure S26 and SM 24, Figure S27) [73,74,75,78]. Comparative structures of selected distorted NbO_6_ octahedrons from literature data are displayed in SM 25, Figure S28 after showing details of geometry for Nb centers in **1**. Similarly, geometries of selected distorted WO_6_ octahedrons are presented in SM 26, Figure S29 for comparison with those discussed in the paper. Somewhat similar structure motif with pentagonal channels occupied by metal cations has been observed before in other structures of bronzoids [79,80] and other oxides in general [69,78]. Moreover, in the structure of **1** there are two channels running along *c*-direction with the smallest distances across them equal 3.536 Å (O23---O23′) and 3.880 Å (O4---O17).

**Figure 8:**
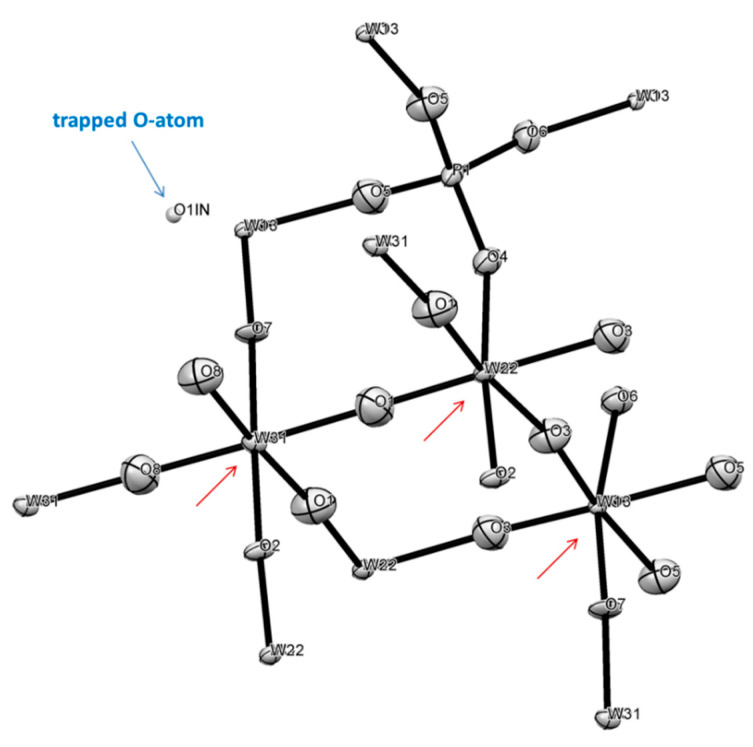
The ASU in the crystal structure of compound **2**, P_4_Mo^VI^_4.17_W^V^_I5.17_W^V^_2.56_O^II^_44_, O^I^_1.44_ in an ORTEP representation drawn at 50% thermal ellipsoids probability level. Red arrow indicate mixed metal sites.

**Figure 9:**
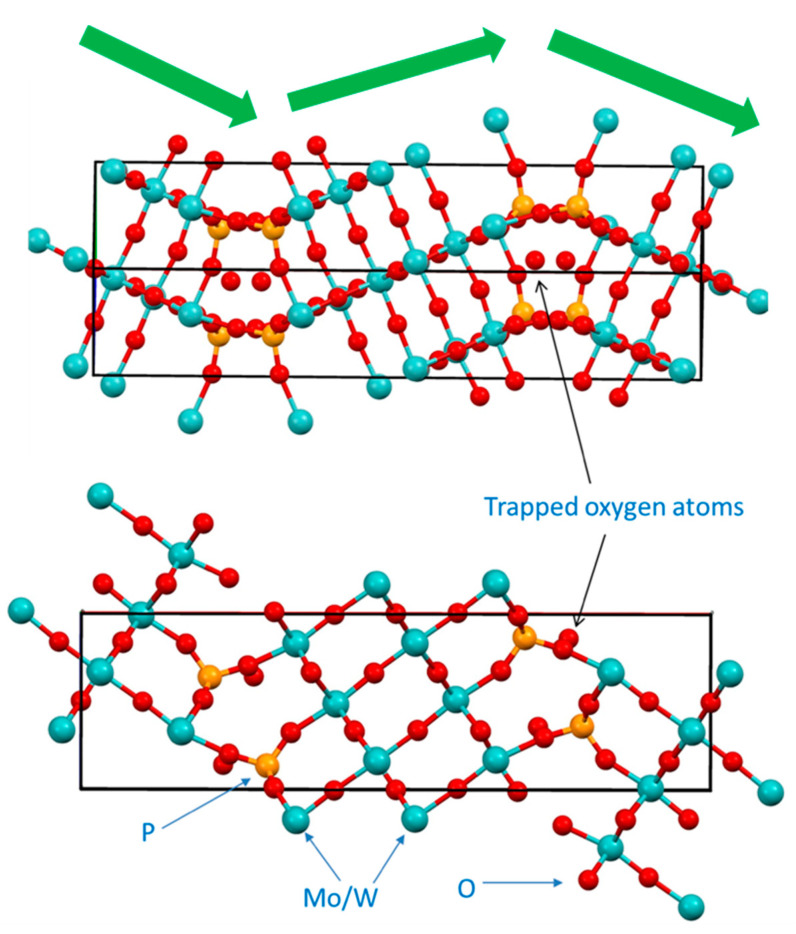
Organization of crystal structure of **2**: top panel—view along [1 1 0] direction, bottom panel—view along *b*-direction. Green arrows show zigzag motif of plates in the structure.

Shown in Figure 8 and Figure 9, ASU in the structure of **1** represents a thick large plate. It is connected with adjacent plates above and below in the crystal lattice, with the help of nine bridging oxygen atoms: O1, O5, O8, O11, O12, O16, O18, O20 and O25 forming a layered structure along *c*-direction with unequal distances between puckered plates: the average M-O distance between upper plate is 2.121 Å, lower plate 2.160 Å, while the same average distance inside the plate is 1.983 Å (SM 27, Chart S2). This feature was the base for the PLATON classification of the overall structure motif of **1** as 2D polymer. There is corner-sharing and edge-sharing type of lattice organization. The latter is seen at pentagonal bipyramid occupied by pnictogens atoms (SM 17, Figure S20). Inside one layer (plate) adjacent MO_6_ (M = Nb, W) heavily distorted octahedrons are well aligned. When analogous upper and lower plates are added to the base plate, transition metal atoms of W and Nb in the base plate adopt considerably distorted octahedral structures geometries which are presented in SM 23, 24. Similar severely distorted polyhedra of d^0^ centers were previously reported in numerous publications [32,69]. The structure of compound **1** is unique and its motif differs from any other known structure of Nb-oxides and at the same time does not resemble any of the W-oxides.

#### 2.7.3. Structure of **2**

For a needle habitus crystal of this compound, which was selected for crystallographic characterization, it was possible to conduct face indexing for numerical absorption correction (SM 28, Figure S30). Analysis of precession images from 0kl, k0l and kl0 planes clearly ruled out modulation in the crystal lattice, and the specimen was found to be true single crystal. This compound is a Mo/W phosphate bronze of “P_4_Mo_12_O_44_” type with chemical formula P_4_(Mo/W)_12_O_45.44_, which indicates the presence of Mo(V) and W(V) centers to balance charge. The structure resembles that of orthorhombic Mo_4_O_11_, but has different unit cell parameters, density and symmetry: the structure of **2** is centrosymmetric in Pmna (#64) with density of 5.25 g/cm^3^, while the former is in Pna2_1_ (#33) with density 4.17 g/cm^3^ [81]. Moreover, instead of the Mo atom in tetra-coordinated environment we have the P atom. The absence of the SHG signal from single crystals of compound **2** clearly ruled out non-centrosymmetric lattices. The first interesting finding here was the absence of arsenic in the structure (Figure 8), albeit it was originally present in the ampoule. Most likely, As_2_O_3_ was sublimed out of the reaction mixture earlier, and then its incorporation into the crystal lattice would occur. The second discovery was finding that all transition metals sites are shared between Mo and W which was used as a reducing agent. The degree of isomorphous substitution is different. The structure converged and was refined at the following SOFs for sites occupied by transition metals: Mo1 = 0.477 shared with W3 = 0.523, Mo2 = 0.348 shared with W2 = 0.652 and Mo3 = 0.162 shared with W = 0.838.

The polyhedral representation of crystal structure of **2** is presented in SM 29, Figure S31; SM 30, Figure S32, while geometry around metal centers represents distorted octahedrons and is detailed in SM 31, Figure S33; SM 32, Figure S34. The average metals-O distances are: at Mo1/W3 site 1.907 Å, Mo2/W2 site 1.915 Å, and Mo3/W1 site 1.900 Å. In all the centers there is distorted octahedral environment of metal ions with the least distorted being at the Mo1/W3 site. The geometry of the P(V) center is usual for the phosphate group.

An EPR spectrum evidenced the presence of reduced states +5 of transition metals in compound **2** (Figure 4). A broad signal at room temperature and 80 K strongly suggests coupling of unpaired d-electrons of W and Mo centers. Based on significantly lower redox potential of W, as compared to Mo, it is most likely that the former metal is reduced to a greater extent. Different oxidation states of these metals have slightly different sizes [82].

The lowest averaged M-O bond in the structure of compound **2** for Mo3/W1 pair may suggest that this center contains predominantly +6 metal ions. For the same reason, the greatest averaged M-O bond in the structure is for the Mo2/W2 site, which is also the most geometrically distorted (SM 31, 32), and it may suggest that there are predominantly +5 metal centers. However, the X-ray analysis cannot provide distinction between the scattering power of Mo(V) vs Mo(VI) and the same for W centers, and at this point, it is difficult to make a sound statement regarding places for reduced metals presence. The overall structure of compound **2** can be described as zigzag 2D polymer comprised of thick plates (Figure 9) that overlay each other by additional coordination of bridging O-atoms to form a 3D carcass.

The most pronounced feature of this structure is the presence of the oxygen atom of inclusion (Figure 8 and Figure 9) which was confirmed by the results of the EPR spectroscopy presented above (Figure 5). A relatively broad line width of that EPR signal (~250 G) is evidence that the electron of trapped oxygen atom O^1−^ experiences some weak interactions with neighboring reduced metal centers having Mo(V)/W(V) oxidation state with one unpaired electron. The spectrum of confined oxygen atoms observed in our work is consistent with recent similar findings [83]. The shortest distance between O1IN and W2(Mo2) center is 3.241 Å, while the distance between two O1IN centers is 4.385 Å. EPR signals disappear when a sample of compound **2** is heated under N_2_ atmosphere (during TG/DSC studies) which suggests its reductive nature. However, at this stage it is difficult to pinpoint what has happened upon heating, and species were obtained upon such reduction since no analysis of the purging gas was performed.

#### 2.7.4. Structure of **3**

For the thin blue plate habitus crystal of this compound it was possible to conduct face indexing for numerical absorption correction. The analysis of precession images from 0kl, k0l and kl0 planes evidenced absence of modulation in the crystal lattice. Compound crystallizes in non-centrosymmetric, polar Pma2 space group (Table 3). However, the best specimen selected for studies was found to be twinned and refinement converged when the model was used as an inversion twin (BASF = 0.167).

Similarly to previously described compounds **1** and **2**, this compound also represents mixed metal, mixed valence bronze based on Mo-oxide nets. Because of the way of preparation of this compound, there is no P in the lattice, and pnictogen element As in oxidation state +3 with active 4s^2^ lone pair. However, all metal sites are shared between Mo and W atoms. Polyhedral representation of crystal structure of **3** is presented in SM 33, Figure S35 and SM 34, Figure S36, while geometry around metal centers represents distorted octahedrons and detailed in Figures SM 35–37. The MoO_6_ (M = Mo, W) corner-shared octahedrons are tilted with respect to each other both inside one layer and between layers. Inside one (the ASU) layer tilting angles between (Mo/W)1, (Mo/W)2, (Mo/W4) and (Mo/W)5 are less than 1° making them practically lined up. The most tilted are octahedrons in the 8-membered metallocycle (Mo/W)2-(Mo/W)4-(Mo/W)3 with those angles being 2.49°, 4.89° and 7.23° for the (Mo/W)2---(Mo/W)3 pair. Individual octahedrons are also twisted with respect to each other. Thus, twist angle between them in one layer of the ASU are the following: (Mo/W)1---(Mo/W)5 = 20.43°, (Mo/W)1---(Mo/W)2 = 19.97°, (Mo/W)2---(Mo/W)3 = 26.02°, (Mo/W)2---(Mo/W)4 = 34.72°, (Mo/W)3---(Mo/W)4 = 45.22°, (Mo/W)4---(Mo/W)5 = 21.23°. Similar distortions were observed in the structure of MoO_3_ (SM 38, Figure S40).

**Figure 10:**
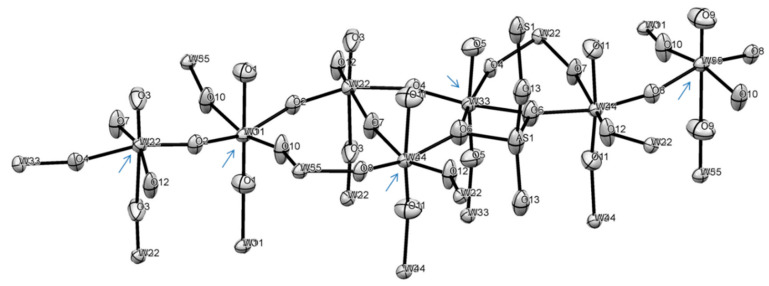
The ASU in the crystal structure of compound **3.** Arrows show mixed-metals sites.

The ASU in the structure is shown in Figure 10 and is somewhat resembles that for compound **1** in a sense of being a thick puckered plate comprised of 8-, 6- and 4-membered metallo-cycles. The 8-membered ring is twisted (dihedral angle 28.03°; SM 39, Figure S41) contrary to that in the structure of **1** described above. The 6-membered ring is also twisted with dihedral angle 12.16°, while 4-membered As1-O6-W3/Mo3-O6 is completely planar. Atoms As1, W3/Mo3 and W5/Mo5 are located on the mirror plane, while W1/Mo1 atoms in the middle of the unit cell are on 2-fold roto-inversion (S_2_) axis (Figure S11). All O-atoms in the structure, except O6 connected to As1 atoms, act as bridges between two metal centers. In the core of the net in the ASU, metal centers are laying on two planes which are 0.652 Å apart (SM 39, Figure S41). The four-membered ring containing As(III) atom is planar. The geometry at the pnictogen atom is, actually, one of the rare finds and certainly is a peculiarity of the crystal structure of compound **3**. It best described as seesaw, and details are presented in SM 40, Figure S43. A place for the 4s^2^ lone pair is clearly inferred in an open cleft between O13-As1-O13′.

**Figure 11:**
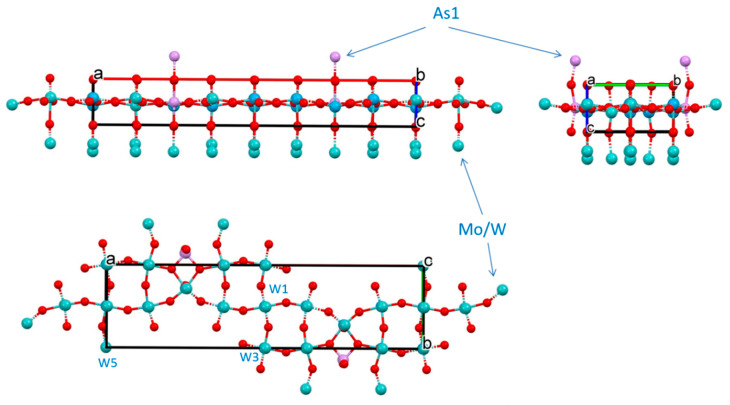
Three orthogonal projections—views along *b*, *c* and *a*—of the unit cell in the structure of compound **3.**

The core ASU of the structure of compound **3**, represents 2D polymeric flat large plate (Figure 11) that is connected to the same units above and below it with the formation of 3D carcass by means of bridging oxygen atoms. With the addition of those O-atoms from adjacent plates, the coordination environment of transition metals represents highly distorted octahedrons (SM 35–37), which is rather typical for oxides of molybdenum [69]. Contrary to the structure of compound **1**, no significant bond lengths difference between plates stacked along c were observed here.

In the structures of **1**, **2** and **3,** partitioning between different oxidation states of Nb, W and Mo is difficult to establish at this stage, and a much higher quality X-ray dataset (which, in turn, depends greatly on a quality of the crystal) is necessary for this task. However, the combined contribution of individual metal centers into the overall charge balance in chemical composition is sound.

#### 2.7.5. Structure of **4**

**Figure 12:**
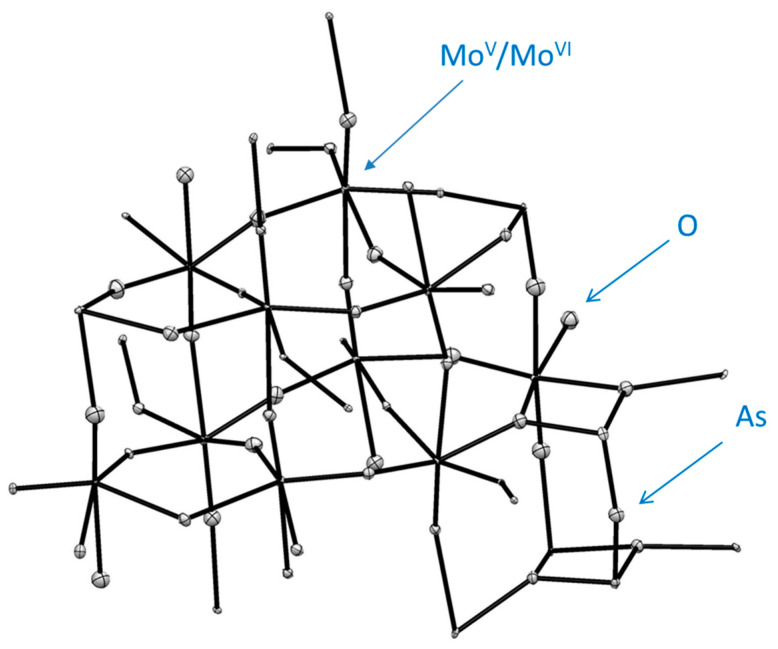
The ASU in the crystal structure of compound **4,** As_2_Mo^V^_4_Mo^VI^_6_O_31._

**Figure 13:**
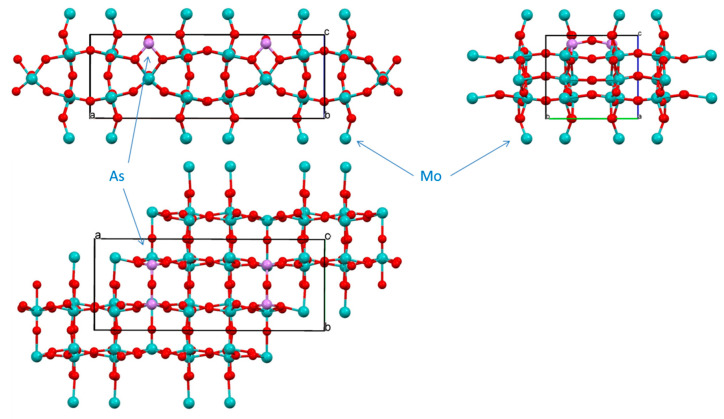
Three orthogonal projections of the unit cell in the structure of compound **4:** views along *a*, *b* (top right) and *c*-direction (bottom) respectively. The arrow indicates the direction of the polar vector in this non-centrosymmetric structure.

For thin red-bronze plate habitus crystal of this compound it was not possible to conduct faces indexing for numerical absorption correction. Analysis of precession images from 0kl, k0l and kl0 planes demonstrated the absence of modulation in the crystal lattice. This stoichiometric compound crystallizes also in the non-centrosymmetric, polar Pma2 space group (Table 3). The overall structure is similar to that for compound **3** above, with the exception of the absent W in Mo-occupied sites. It is a much more highly distorted structure, however. The reduction of Mo^+6^ was performed using Mo metal powder and the resulting compound contained only mixed valence transition metal—molybdenum. The formula for the compound **4** is As_2_Mo_10_O_31_, or with identification of oxidation states it is As_2_Mo^V^_4_Mo^VI^_6_O_31_ (Table 1 and Table 3). The ASU in the structure is shown in Figure 12, while the unit cell content is presented in Figure 13. This compound forms elegant 3D framework contrary to the above 2D-polymeric layered phases **1**–**3**. Polyhedral representation of structure of **4** can be seen in SM 41, 42; Figures S44 and S45, while details of geometry at individual Mo-centers are presented in SM 43, Figure S46. There is exclusively corner-sharing lattice buildup. The MoO_6_ corner-shared octahedrons are tilted with respect to each other. The most tilting occurs inside the layer, while between layers, octahedrons lined up almost collinearly along *a*-direction (Figure 13). Some representative tilt angles inside the layer are as follows: Mo1---Mo4 = 6.82°, Mo2---Mo3 = 5.78°, Mo3---Mo6 = 10.54°, Mo4---Mo5 = 11.43°. The same tilt angles in adjacent octahedrons between layers are, for example, Mo1---Mo2 = 1.61°, Mo3---Mo4 = 1.17° and Mo5---Mo6 = 0.97°. Individual octahedrons are also twisted with respect to each other (SM 43). Those angles are 49.41° for Mo1---Mo5, 25.15° for Mo1---Mo4, 27° for Mo4---Mo5, 18.84° for Mo2---Mo3, 45.22° for Mo2---Mo6, 24.14° for Mo3---Mo6 and joined via O5 25.36° angle between symmetry related Mo1---Mo1.

In the structure of **4** there are also 8-, 6- and 4-membered metallo-cycles (Figure 13). There three eight-membered cycles in this structure. One is Mo1-O4-Mo4-O13-Mo3-O14-Mo2-O5, and is severely twisted with the dihedral angle equal to 36.43°—much larger than that for the compound **3**. The second ring is Mo4-O13-Mo3-O12-Mo4*-O13*-Mo3*-O12* and is bent at 16.89°. The third 8-membered ring is Mo3-O11-Mo6-O1-Mo5-O17-Mo4-O12 and is also bent with the value of the dihedral angle being 26.11°. Somewhat similar cyclic arrangements involving heavy metals and oxygen atoms were also found in the structure of sodium-barium niobate [84]. The six-membered ring Mo3-O14-Mo2-O8-Mo6-O11 adopts a chair conformation and the dihedral angle between the two selected planes is 19.02°. In this structure, the atom of As1 takes a special position, being on the mirror plane, while Mo5, Mo6, O1, O9 and O10 are occupying both the mirror plane and the gliding plane.

**Figure 14:**
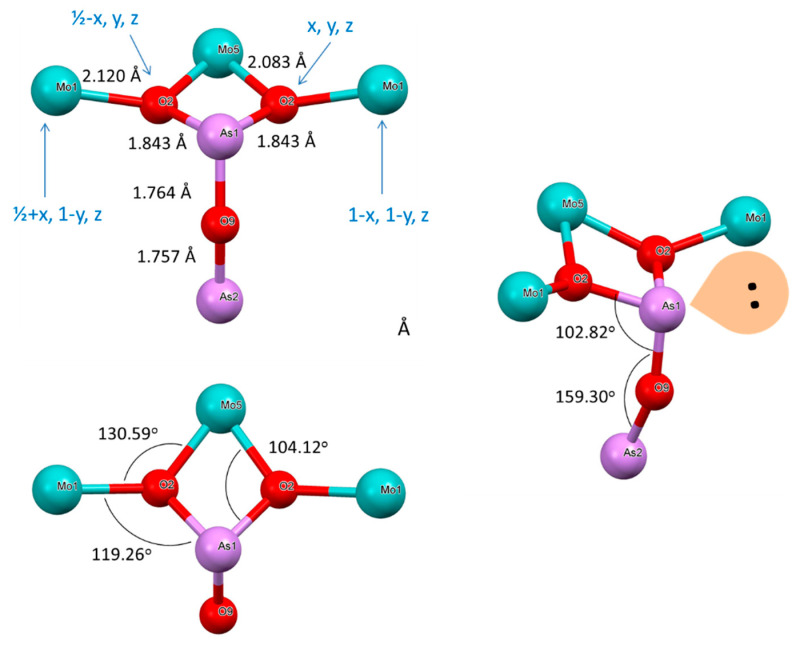
Details of geometry around As1 atom in the structure of **4**. Symmetry codes are shown in blue.

The four-membered ring involving pnictogen—As1-O2-Mo5-O2 is not planar and the dihedral angle equals 13.07°. The geometry around the Arsenic atom, which has stereo-active lone pair, is shown in (Figure 14). It is best described as a trigonal pyramid.

#### 2.7.6. Single Crystals Electrical Conductivity Studies

One of the objectives of the investigation was to evaluate the ability of new compounds to act as electric conductors. Details of experimental setups and formulas for calculations are given in SM 44, Figure S47; SM 45, text 48. Since these new compounds were obtained at high temperatures, there is good potential for their use in electronic devices, and even in photovoltaic cells, when this particular aspect of conductivity will be further investigated. At first, we needed to assess whether compounds **1**–**4** demonstrate conductivity at all. Studies of powdery materials represent a significant experimental challenge, because of the need for high pressure and high vacuum equipment to eliminate air gaps between individual crystallites [85]. Another issue is the attachment of electrodes for measurements which are highly dependent on the physical stability of pellets obtained in such a way. Single crystal measurements in such a situation represent the most reliable method of compounds’ electrical conductivity evaluation, although this has its own difficulties. The most noticeable is the actual crystal size, which requires all operations to be carried out under the microscope. In this situation, the most pressing problem is proper attachment of the electrode using conducting glues. We had tried several kinds: colloidal sliver in paste, gold paste, and graphite paste. The most suitable and reproducible data were obtained using the last glue. As a dielectric base, we used microscope borosilicate glass cover slips, and all manipulations with single crystals of compounds **1**–**4** were carried out using thin copper wires, thin glass fibers, or Hampton Instrument Mo-needles, used for work during crystallographic research. All measurements were carried out within several minutes, and electrical parameters were recorded in time-dependent fashion. The time dependence was expected, because of the very small crystal size and the fast depletion of charges upon applied bias. Moreover, the direction of current was switched to opposite, to prove invariance of crystals of **1**–**4** to applied bias.

**Figure 15:**
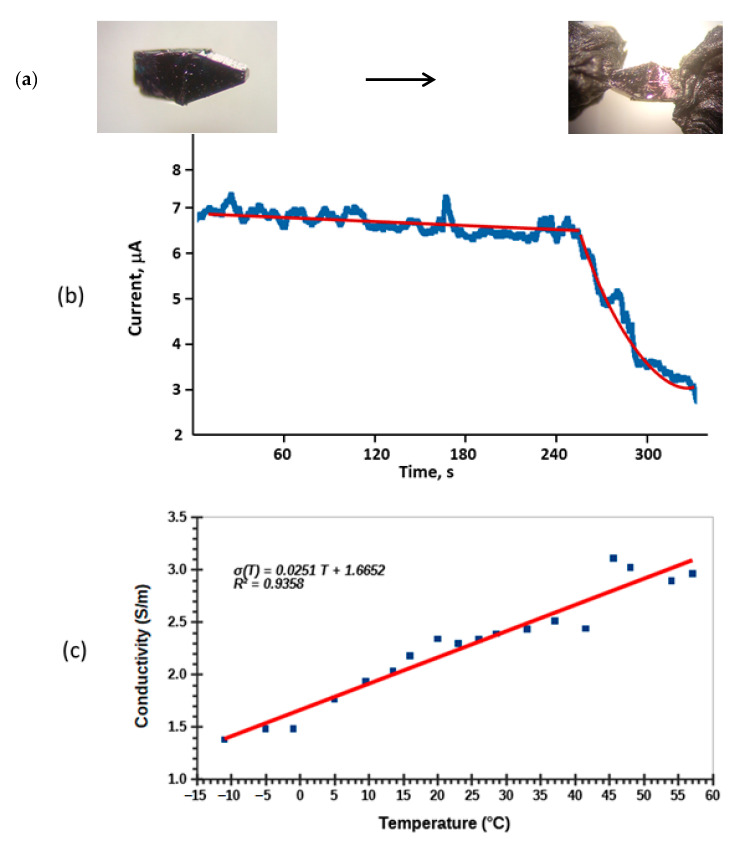
Single crystal of compound **4** used for electrical conductivity studies before and after being prepared (**a**), time dependence of current for crystal of compound **2** and its bifunctional fit (**b**), and temperature dependence of conductivity for compound **3** depicting its semiconductor behavior (**c**).

The compounds appeared to be semiconductors, with a very variable conductivity range from sample to sample, as was shown by variable temperature studies in a modest temperature range between −15 and +65 °C (Figure 15). At this stage of our investigations into these new solids, we were interested in proof of their belonging to the family of semiconductors. In order to investigate *ohmic* behavior, different current/voltage loads were applied. In order to properly calculate compounds’ electrical conductivity, accurate crystals’ dimensions have to be measured. For that purpose, a special cover slip, with a grid used for cell work in biological experiments, was used (SM 31). The results of measurements are summarized in Table 2, while the most representative images of single crystals used for measurements, voltage/time profiles, and temperature dependence, are presented in Figure 15.No dramatic changes in conductivity were observed when the applied bias was reversed. Compound **1** was tentatively found to be dielectric, albeit the results may be inconclusive, without further studies of considerably larger specimens, due to the very small size of its crystals. Single crystals of compound **2** recovered from the same quartz ampoule had two different morphologies: plates and needles (Figure 1). They displayed considerably different electrical conductivity (Table 4). We relate this phenomenon to different degrees of reduction of metal centers in both types, albeit their unit cell parameters were very close.

Crystals of compounds **2**–**4** exhibited a pronounced temperature dependence that classified them as semiconductors, with compound **4** being the most conductive. Doping of Mo-bronzes with W has recently been done, and showed useful successive charge density wave (CDW) transitions [86]. Because of the presence of lower oxidation state metal centers (reduced species) we assumed that the observed conductivity was due to movement of electrons. Investigations of the Hall Effect (or Seebeck effect) on such small objects were prohibitively difficult experimentally and considered unrealistic to carry out at this time.

Another unforeseen problem that we found was the gradient of different oxidation states throughout the crystal, as well as the gradients of different metals centers, which resulted in slightly different electrical conductivity between crystals of the same compound.

## 3. Experimental Part

### 3.1. Materials and Preparations

All metals and non-metals oxides used in this work were obtained from commercial vendors (Aldrich (St. Louis, MO, USA), Mallinckrodt (Staines-upon-Thames, UK), Strem Chemicals (Newburyport, MA, USA), and used without further purification. Elemental Mo, W and Nb as fine powders were of high purity grade. Silica ampoules, 16 mm OD, were obtained for the synthesis of compounds from ChemGlass Co (Vineland, NJ, USA).

The general design of making of new phases is depicted in Table 5. The preparation of compounds was done in quartz ampoules to which thoroughly ground (using agate mortar and pestle) mixtures of ingredients were loaded, in amounts not exceeding 2 g per vessel. Ampoules were vacuumed at +40 °C for an hour, and then sealed using a high temperature blow torch. Ampoules were then placed in tubular furnaces that had small temperature gradients, and heated to ~950 °C for 10 days, with slow cooling at a rate ~10°/h when heating devices were turned off. The furnaces temperature gradient was estimated to be ~40–60 °C between the center and the tube end. In the cooler parts of the ampoules clearly visible beautiful crystalline products of variable colors were clearly visible, while at the bottom of the ampoules, we observed dark-colored powdery residue (SM 1, Figure S1). Opening the ampoules afforded both types of products. Only sublimed crystalline specimens were used for investigations, since the bottom residues did not contain pure compound, and represented mixtures of variable compositions.

More specifically, for the preparation of compound **1,** (P/As)_4_(Nb/W)_30_O_83.08_, fine powders of WO_3_, P_2_O_5_, As_2_O_3_, Nb_2_O_5_ at molar ratios 5:3:2:5 respectively, and 150 mg of metallic Nb powder as a reducing agent, were used. Compound **1** represents very thin, micron-size blue-grey needles, which were obtained in ~62% of the yield. Actual composition was determined by single crystal X-ray analysis, and found to be As_1.94_P_2.06_Nb_27.77_W_2.23_O_83.08_.

For the preparation of compound **2**, P_4_(Mo/W)_12_O_45.44_, we used fine powders of WO_3_, P_2_O_5_, As_2_O_3_, MoO_5_ at molar ratios 4:3.5:1.5:4 and 100 mg of metallic W powder. Fragile crystals of compound **2** were found to adopt two shapes, needles and plates, both having a distinctive bronze sheen. The actual composition of compound **2** was determined by X-ray analysis, and found to be P_4_Mo^VI^_4.17_W^VI^_5.27_W^V^_2.56_O^II^_44_, O^I^_1.44_, which contained some amount of trapped oxygen.

Compound **3** was obtained from the reaction mixture containing WO_3_, As_2_O_3_, MoO_5_ at molar ratios 1:1.5:3 and 150 mg of metallic Mo. Beautiful, fragile blue prisms of **3** had a uniform shape, and a slight bronze shine, obtained in ~28% of the yield. The actual composition of compound **3** was determined to be As_2_(Mo/W)_14_O_44_, according to data of single crystal analysis.

Compound **4** was obtained from the reaction mixture of As_2_O_3_ and MoO_3_ (1.5:5) which also contained 100 mg of metallic Mo powder. Dark red-purple prisms of **4**, with a pronounced bronze sheen, were collected from the quartz tube in *ca* 44% of the yield, leaving a considerable amount of brown/black powder behind. The actual composition of compound **4** was found to be As_2_Mo^V^_4_Mo^VI^_6_O_31_, according to the data of its single crystal analysis and necessity to balance charge.

After recovery, all the above crystalline compounds were stored for study in a desiccator charged with H_2_SO_4_(c).

#### Safety Note

Preparations of these mixed metals/mixed oxides involve the use of arsenic in the form of its As_2_O_3_ oxide, which is very toxic. Special care should be taken in handling this compound, the products of its reactions, and waste. All work must be carried out under the ventilation hood, wearing gloves and a face mask, to avoid unintended inhalation of fine dust particles containing As, during the reaction mixture samples preparation, which includes grinding. Phosphorous pentoxide, P_2_O_5_, is highly moisture-sensitive, reacts violently with water, and requires special attention in preparation and waste handling.

### 3.2. Methods of Characterization

#### 3.2.1. Optical Microscopy

The crystals’ selection and overall analysis of visible morphology, and the description of the obtained materials in studied oxide systems were conducted using the Motic microscope at 40× magnification. Digital photographs were taken using a digital camera of 3.2 Mp resolution. The selection and analysis of single crystals for X-ray analysis were carried out using the Meiji stereo microscope equipped with a rotating polarizer and videocamera.

#### 3.2.2. SHG Studies

The experimental setup is present in Appendix A. This non-linear optical effect was observed as registration of flow of 532 nm wavelength photons from the laser source NIR YAG, carrying 1064 nm radiation passing through a single crystal of examined compound. Whenever possible, circular polarization measurements were performed as well. Detection of the SHG signal was done with help of a Tektronix oscilloscope. A pure sample of optically active crystalline quartz (SiO_2_, trigonal P3_2_21 space group #154) was used for the instrument calibration.

#### 3.2.3. EPR Spectroscopy

A Bruker EMXplus X-band EPR spectrometer, equipped with a cryostat system operating at 296 and 80 K with the field sweep from 200 to 4000 G. The sensitivity of instruments was checked by a recording spectrum of solid Al_2_(SO_4_)_3_ containing 1% of Cr^3+^ in it. Samples of crystals were investigated in quartz capillaries.

#### 3.2.4. Micro-Raman Spectroscopy

Spectra were recorded at room temperatures using a Horiba Lab Ram HR 800 Micro-Raman Complex with laser illumination at 785, 532, 430 nm. Spectra were recorded in the range of shifts 50–1200 cm^−1^.

#### 3.2.5. UV-Visible Spectroscopy

Spectra of diffuse reflectance (SDR) were recorded at room temperature on a Cary 100 Bio spectrophotometer in the range of 200–800 nm, equipped with an integrating sphere from LabSphere (North Sutton, NH, USA). This unit was calibrated using MgO as a white standard. The baseline was recorded using a White Millipore HA 0.45 µm, 20 mm diameter filter, attached to a holder with a transparent Scotch tape. The solid compounds **1**–**4** were ground in an agate mortar, and attached as fine powders to the Millipore filter membrane with clear Scotch tape. The spectra were recorded in the % reflectance mode, but for conventional viewing and analyses, are presented in the absorbance mode. The full line shape analyses (spectroscopic envelope deconvolution) were carried out with help of Origin-6 software.

#### 3.2.6. Thermal Analysis

Traces of samples’ weight loss and heat flow were recorded with the help of Q-600 TG/DSC analyzer from TA Instruments (Delaware, DE, USA) in the range of 30–1300 °C. Two types of gas carriers were used: UHP grade argon, and air, both at 100 ± 1 mL/min flow rate and 10°/min heating rate. The brand new alumina crucible was calcined prior to each experiment using the propane torch. Data were processed and graphed using the TA Universal Analysis software package.

#### 3.2.7. X-ray Diffraction Studies

Single crystal analyses of samples of compounds were carried out using APEX2 diffractometers: one was equipped with filtered and monochromated Mo-radiation, while the other was at the synchrotron facility at UC-Berkeley for very small and thin crystal samples, at the Advanced Light Source (λ = 0.7749 Å monochromated through Si [1 1 1] crystal). Suitable single crystals were inspected, selected, and handled in NVH parathon oil, with subsequent mounting on a thin glass fiber, or placed into the CryoLoop and then attached to the copper pin positioned on the goniometer head of a diffractometer equipped with a CCD area detector. All data sets were measured at several temperatures to investigate possible polymorphism. The intensities for the latter radiation were integrated from four series of 364 exposures, each covering 0.5° in ω, with the total data set being a sphere. The space group determination was done with the aid of XPREP software [72]. The absorption correction represented a challenging problem due to the very small crystals’ size and the practical difficulty of face-indexing using a videomicroscope. Thus, a multi-scan method was used most of the time, by the SADABS program that was included in the Bruker AXS software package [72,87,88]. All structures were solved by direct methods and refined by least squares on weighted *F^2^* values for all reflections, using the SHELXS-1013 (Sheldrick 2013, SHELXL-2013, https://www.iucr.org/resources/other-directories/software/shelxl2013 (accessed on 27 January 2021) Madison, WI, USA). The structures reported herein did not have apparent errors, and are well refined, with details presented during discussion of results. Crystal and refinement data, and selected bond lengths and angles, are listed in appropriate Tables in the text. Drawing the crystal structures and packing diagrams was done using the ORTEP 3v2 [89,90] and Mercury software packages [91]. Thermal ellipsoids in the presented figures are drawn at their 50% probability level. Powder XRD patterns were recorded on D80 Discover, Cu-tube (λ = 1.5406 Å), at 293 K. All four presented in this study crystal structures have been deposited at the CCDC site.

#### 3.2.8. Single Crystals Electrical Conductivity Measurements

A single crystal specimen selected for studies was retrieved from the sample container with a thin glass rod and placed on a borosilicate glass slide (Corning Incorporated 2948-75X25). The glass slide had a thin layer of fresh nail polish (SinfulColors Top Shine Couche Finition Brilliant, New York, NY, USA) to anchor the crystal into the surface. Crystals’ dimensions were measured under a microscope, using the grid of a blood cell counting chamber (grid 0.05 mm). The nail polish was used to glue the golden wires to ends of the crystal. A thin golden wire (diameter 0.2 mm; ALDRICH (St. Luois, MO, USA), was attached to each end of the crystal using PELCO Conductive Carbon Glue (Ted Pella, Inc. (Redding, CA, USA). The wiring was secured with narrow strips of Scotch tape and nail polish micro drops. Neither the nail polish, nor the tapes were allowed to contact the sample crystal. The samples were allowed to dry for about two hours before the conductivity measurements.

The conductivity measurements were carried out with RBD Instruments’ 9103 Auto-ranging Picoammeter in properly grounded with individual lead Faradaic cage. The picoammeter’s range was manually chosen according to the sample. Cypress Systems Omni-101 Microprocessor Controlled Potentiostat was used as the voltage source. Voltage used in the experiments was 2048 mV (direct current) unless otherwise stated in the notes. The filter was set to a value of 10 ms. Sensitivity was adjusted according to the observed current of the sample (100 nA/V or 1 µA/V). Background noise was recorded from a zero plate, which was made to mimic the test sample, but without a crystal between the carbon glue junctions. Background noise (0.004 nA) was subtracted from the observed sample currents during data processing. Each sample was measured twice. During the second test run, the polarity of the potentiostat was inverted. Measurements were carried out inside a Faraday cage (SM 44, Figure S47). An average current was calculated from the first 240 s of each experiment, unless otherwise stated in the notes.

## 4. Conclusions

The conducted studies can be summarized as follows:

- It was possible to prepare mixed metal, mixed valent solid phases using typical in solid-state chemistry high temperature procedures, although the process needs to be better designed to increase yield of crystalline phases.
- All prepared compounds represent metal oxides-based bronzes with only one compound (**4**) being a stoichiometric phase, while other three new phases are solid solutions with different degree of isomorphous substitution of transition metal cations.
- UV-visible and EPR spectroscopic methods confirmed reduction of d^0^ transition metals Mo, W and Nb to their lover oxidation states +5 and +4 respectively.
- Crystals of resulting bronzes are dichroic and possess shiny colors and three out of four compounds crystallized in chiral (compound **1**, P2_1_2_1_2) and polar (compounds **3** and **4**, Pam2_1_) non-centrosymmetric space groups, which was initially suggested by the observed strong SHG effect on their single crystals.
- Three new phases showed pronounced *type 4 **photosalient effect**—*a motion of a crystal in the laser beam—which is attributed to rapid thermal expansion of single crystals upon irradiation with the NIR YAG laser (1064 nm) generating heat.
- Combination of crystallography and auxiliary methods allowed determination of a complex content of the obtained phases.
- Crystals of all three new phases **1** and **2** form 2D layered crystal structures demonstrating large flat, thick or bent (**2**) plates of metal-oxygen nets that, being stacked, assemble into 3D framework. The structures of compounds **3** and **4** are different and represent elegant 3D network.
- Introduction of pnictogen element—As(III)—was a justifiable and useful approach to induce distortions in the crystal lattice of compounds that successfully lead to the formation of non-centrosymmetric structures with stereoactive 4s^2^ lone pairs on As-centers in compounds **3** and **4**.
- Electrical conductivity of single crystals of new phases has been measured and evidenced that three out of four demonstrate semiconductor behavior at its high end.

### Future Directions

The next step in investigation of these new solids will be determination of degrees of distortion of MO_6_ octahedrons by method outlined by Halasyamani et al. [69] which requires certain resources and skills that we currently do not have. There are three potential areas of research where our new compounds can be further studied. Non-centrosymmetric oxides exhibit NLO properties, accompanied with a ***photosalient effect*** which can be used as a motion sensor/actuator not requiring electric feed. Another interesting possibility is to further evaluate the usefulness of compounds **1**–**4** in electronics, since they possess semiconductor properties, but not based on classic intrinsic semiconductors such as Si or Ge. Lastly, we discovered by the EPR method, the ability of the new mixed valence oxides outlined above, to be reducing agents in the absence of air. That fact coupled with their insolubility in water and common organic solvents, which opens the way for the development of applications of compounds **1**–**4** as heterogeneous catalysts.

## Figures and Tables

**Table 1:** Numbering Scheme for the Obtained Compounds.

Phase/Compound	Formula	Actual Composition *
**1**	(P/As)_4_(Nb/W)_30_O_83.08_	As_1.94_P_2.06_Nb_27.77_W_2.23_O_83.08_
**2**	P_4_(Mo/W)_12_O_45.44_	P_4_Mo^VI^_4.17_W^VI^_5.27_W^V^_2.56_O^II^_44_, O^I^_1.44_
**3**	As_2_(Mo/W)_14_O_44_	As_2_Mo_10.65_W_3.35_O_44_
**4**	As_2_Mo_10_O_31_	As_2_Mo^V^_4_Mo^VI^_6_O_31_

* determined by single crystal X-ray analysis.

**Table 2:** Results of Studies of Compounds **1**–**4** Using Vibrational Spectroscopy Listing the Most Intense Bands (cm^−^^1^) in Spectra.

Compound	Bonds Present	IR-Spectra	Raman
**1**	Nb-O, P-O, As-O, W-O	790(sh), 850(st.br), 920(st)	936, 838, 789, 740, 376, 284, 149
**2**	P-O, Mo-O, W-O	697(w), 790(br), 1061, 1090(br.st)	978, 829, 820, 742, 442, 260, 174, 71
**3**	As-O, Mo-O, W-O	669(w), 722(w), 802(w), 880(w), 943(br), 1032(br), 1111(br), 1156(w), 1451(br), 1632(st)	984, 858, 833, 806(sh), 557, 368, 267
**4**	As-O, Mo-O	724(w), 803, 820(st), 874(w), 881(w), 950(br), 1030(st), 1056(st), 1109(st), 1161(st), 1457(st), 1640(st)	880, 870, 829, 742, 452, 302, 174

Abbreviations: sh—shoulder, w—weak, br—broad, st—strong.

**Table 3:** Crystal and Refinement Data for Synthesized Compounds.

Compound				
Parameter	1	2	3	4
Type	P_4_Nb_30_O_85_	P_4_Mo_12_O_44_	As_2_Mo_14_O_44_	As_2_Mo_10_O_31_
Composition	(P/As)_4_(Nb/W)_30_O_83.08_	P_4_(Mo/W)_12_O_45.44_	As_2_(Mo/W)_14_O_44_	same as above
Formula, ASU	As_1.94_P_2.06_Nb_27.77_W_2.23_O_83.08_	P_4_Mo^VI^_4.17_W^VI^_5.27_W^V^_2.56_O^II^_44_, O^I^_1.44_	As_2_Mo_10.65_W_3.35_O_44_	As_2_Mo^V^_4_Mo^VI^_6_O_31_
Crystal system	orthorhombic	orthorhombic	orthorhombic	orthorhombic
Space group	P2_1_2_1_2 (#18)	Pnma (#62)	Pma2 (#28)	Pma2 (#28)
Radiation	Mo, Kα	Mo, Kα	Mo, Kα	synchrotron, 0.7749 Å
T of dataset, K	250	250	100	150
Unit cell constants:				
*a*	12.2400(14)	23.659(3)	27.685(3)	20.1867(9)
*b*	36.672(4)	5.3107(7)	7.2424(7)	7.9878(7)
*c*	3.9436(5)	6.5866(8)	3.9413(7)	7.1909(6)
α	90	90	90	90
β	90	90	90	90
γ	90	90	90	90
Volume, Å^3^	1770.2(4)	827.59(18)	790.25(14)	1159.51(17)
Density, g/cm^3^	4.248	5.383	5.268	4.597
Z	1	8	4	2
µ(mm^−1^)	9.198	28.70	19.04	10.26
F(OOO)	2140	1175	1119	1468
Total reflections	17,769	8002	8658	19,766
Reflections used	3096	808	1631	2905
Number of parameters	308	77	147	200
R(int)	0.0498	0.0256	0.0340	0.0287
Flack parameter	0.43(6)	n/a	0.17(11)	0.080(6)
R1	0.0705	0.0312	0.0457	0.0320
wR2	0.1839	0.0650	0.1153	0.0893
GOF	1.057	1.140	1.213	1.387
Largest peak/hole, A^−3^	4.32/−4.23	2.10/−1.76	4.95/−2.31	1.693/−2.496
Structure dimensionality	2D	2D	3D	3D
Structure volume, A^3^	1365.7 (77.0%)	657.8 (79.5%)	634.8 (80.3%)	935.8 (80.72%)
CCDC	2,053,473	2,053,467	2,053,466	2,065,580

**Table 4:** Results of Single Crystals’ Electrical Conductivity Studies for Synthesized Compounds.

Compound	Crystal Size, mm	Conductivity	Ohmic Behavior Direction 1→ 2 (2→1)	T-Effect
**1**, needle	0.25 × 0.017 × 0.017	143 µS/m (83 µS/m)	insulator	n/a
**2**, plates	0.40 × 0.15 × 0.03	12.4 mS/m (6.5 mS/m)	yes	+
**2**, needles	0.25 × 0.05 × 0.02	620 mS/m (510 mS/m)	yes	+
**3**, prism	0.20 × 0.13 × 0.10	0.93 S/m (0.90 S/m)	no	+
**4**, block	0.20 × 0.14 × 0.11	1.23 S/m (1.23 S/m)	yes	+

**Table 5:** Synthetic Strategy Employed for the Preparation of New Phases.

Phase/Compound	Pnictogens	Transition Metals	Reducing Metal Powder
**1**	P and As	Nb and W (no Mo)	Nb
**2**	P	Mo and W (no Nb)	W
**3**	As	Mo and W (no Nb)	Mo
**4**	As	Mo (no Nb, no W)	Mo

**Figure A1:**
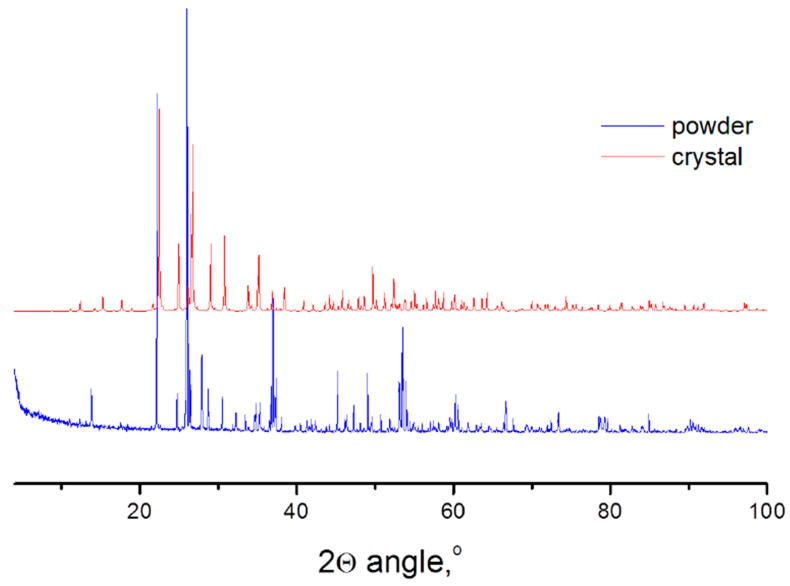
Powder XRD data for two types of material extracted from an open ampoule during preparation of compound 4.

## Data Availability

Crystallographic information files are available through the CCDC though assign submission numbers. Spectral information is available from corresponding author upon request.

## Supplementary Materials

The following associated content titled as Supporting Materials is available online, Figure SM 1, Figure S1: end point in synthesis of compounds in ampoules; SM 2, Figures S2 and S3: experimental setup for second harmonic generation measurements; SM 3, Figures S4, S5: SHG effect in sample of **1**; SM 4, Chart S1: ***photosalient effect***; SM 5, Figure S6: variable temperature lattice changes for **4** and explanation for ***photosalient effect***; SM 6, Table S1: EPR signatures of key transition metals isotopes; SM 7, Figure S7: the EPR spectrum of **3**; SM 8, Figure S8: analysis of the EPR spectrum of compound **4**; SM 9, Figure S9: EPR spectra of **4** before and after TG/DSC experiment; SM 10, Table S2: values of standard redox potentials of As, Nb, Mo and W; SM 11, Figures S10, S11: Raman spectra of compound **1** and **2**; SM 12, Figures S12, S13: Raman spectra of compounds **3** and **4**; SM 13, Figure S14: full line shape analysis of SDR of **2**; SM 14, Figures S15, S16: the XRD precession images for **1** showing lattice modulation; SM 15, Figures S17, S18: reciprocal lattice images for full dataset of reflections of **1**; SM 16, 17 Figures S19, S20: structure of **1** as polyhedrons; SM 18, Figure S21: analysis of planarity of 8-membered metallo-cycle in **1**; SM 19, Figure S22: analysis of planarity of 6-membered metallo-cycle in **1**; SM 20, Figure S23: geometry of As/P center in the structure of **1**; SM 21, Figure S24: angles in pentagonal star shape in the structure of **1**; SM 22, Figure S25: bonds in star-shaped pnictogens center in **1**; SM 23, 24, Figures S26, S27: geometries of metal centers in the structure of **1**; SM 25, Figure S28: literature data for Nb-O bonds in oxides; SM 26, Figure S29: literature data for W-O bonds in oxides; SM 27, Chart S2: organization of layered 2D structure of **1**; SM 28, Figure S30: indexed crystal faces of **2** by videomicroscope; SM 29, 30, Figures S31, S32: representation of the structure of **2** as polyhedrons; SM 31, 32, Figures S33, S34: geometry of metal centers in the structure of **2**; SM 33, 34, Figures 35, 36: structure of **3** in polyhedral representation; SM 35–37, Figures S37–S39: details of geometry of transition metals environment in the structure of **3**; SM 38, Figure S40: literature data for Mo-O bonds in oxides; SM 39, Figure S41: details of structure of M-O net in **3**; SM 40, Figures S42, S43: core structure details of **3** and geometry around of As atom; SM 41, 42, Figures S44, S45: polyhedrons representation of structure of **4**; SM 43, Figure S46: bond length around Mo-centers in the structure of **4**; SM 44, Figure S47: experimental setup for single crystals electrical conductivity measurements; SM 45, Text 1 with details of calculations of electrical conductivity. References [74,92,93,94] are cited in the Supplementary Materials.
