# Supplementary material for: New Solids in As-O-Mo, As(P)-O-Mo(W) and As(P)-O-Nb(W) Systems That Exhibit Nonlinear Optical Properties
Source: Molecules. 2021 Mar 9;26(5):1494. doi: 10.3390/molecules26051494 (PMC7967165; doi:10.3390/molecules26051494)
Supplement: Supplementary file 1 [file molecules-26-01494-s001.pdf]

Pages of Supplementary Data for Article

New solids in As-O-Mo, As(P)-O-Mo(W) and As(P)-O-Nb(W) systems that exhibit non-linear-optical properties

by

Nikolay Gerasimchuk[1*], Lauri Kivijarvi[1], Bruce Noll[2], Meriem Goudjil[3], Soma Khantra[4], Yu Ping[4], Miles Pearson[1], Frank Röminger[5]

* Correspondence: NNGerasimchuk@MissouriState.edu; Tel.: 1-(417)-836-5165

SM 1 **Figure 1**

Principal outcome of the synthesis of new mixed metal, mixed oxides solids carried out in a quartz ampoule. Powder residue was found inhomogeneous and was discarded. Crystals used for studies.

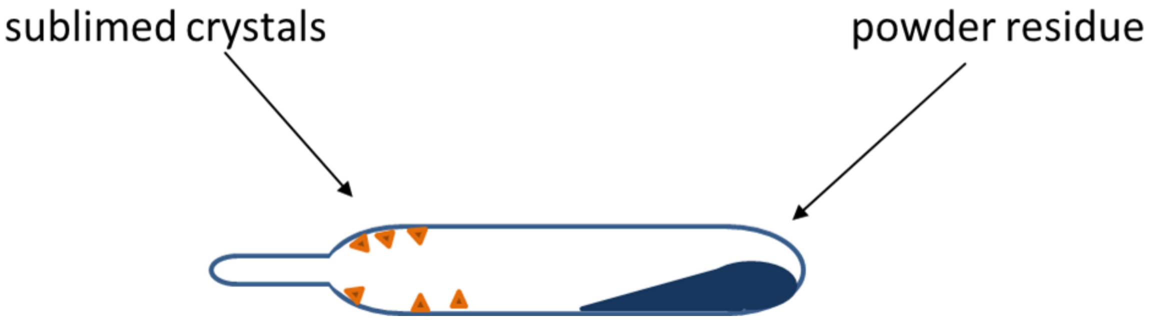

SM 2 **Figures 2,3**

Experimental setup of the second harmonic generation (SHG) measurements: **top** – block scheme; **bottom** – actual laboratory settings showing the videomicroscope for optical alignment of single crystals in the beam, with a red arrow.

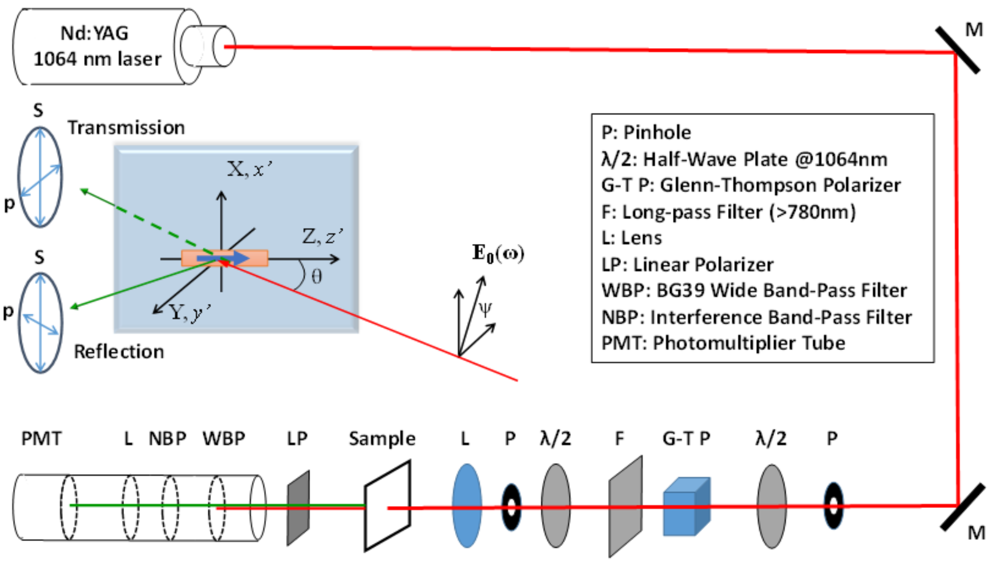

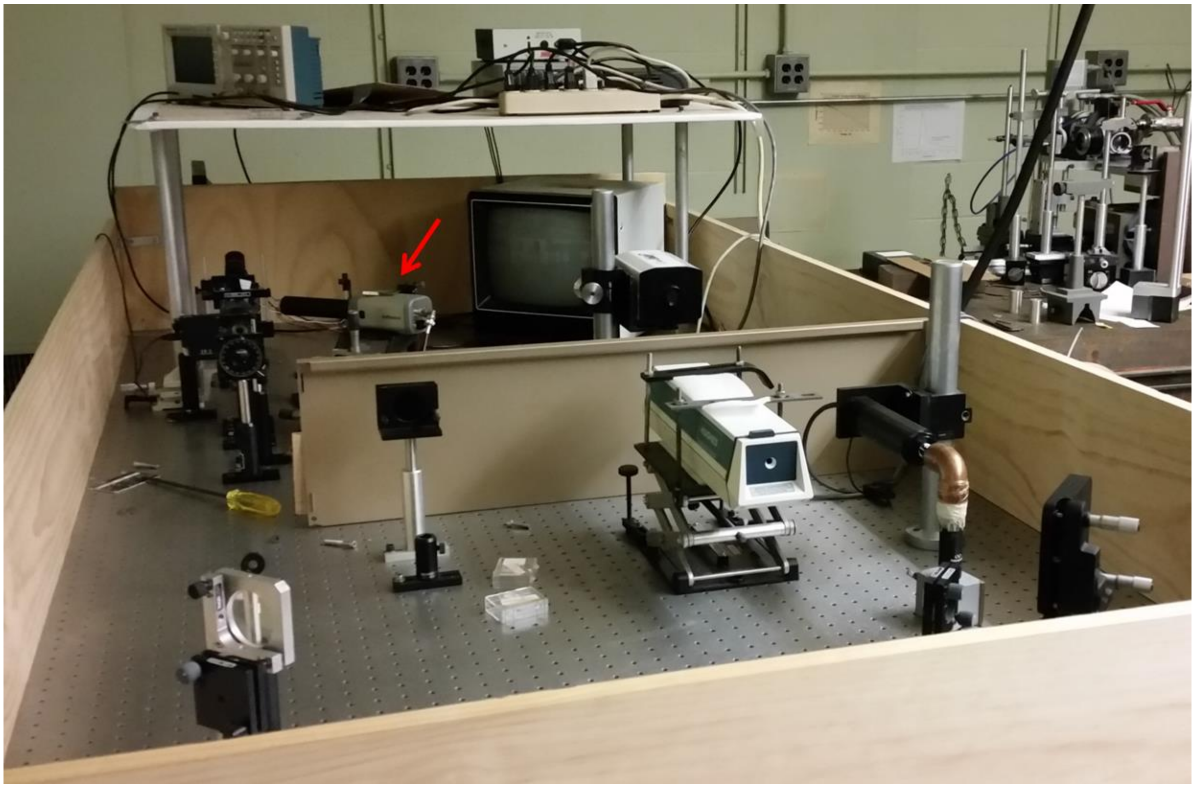

SM 3 **Figures 4,5**

Plotted data of observed SHG effect in rotating of sample **1** with its polarization measurements (**A**). Time evolution of the SHG signal at 532 nm from single crystals of sample **4** (**B**); the SHG signal becomes very small within 2-3 minutes where decrease of its intensity is indicated with arrows.

A

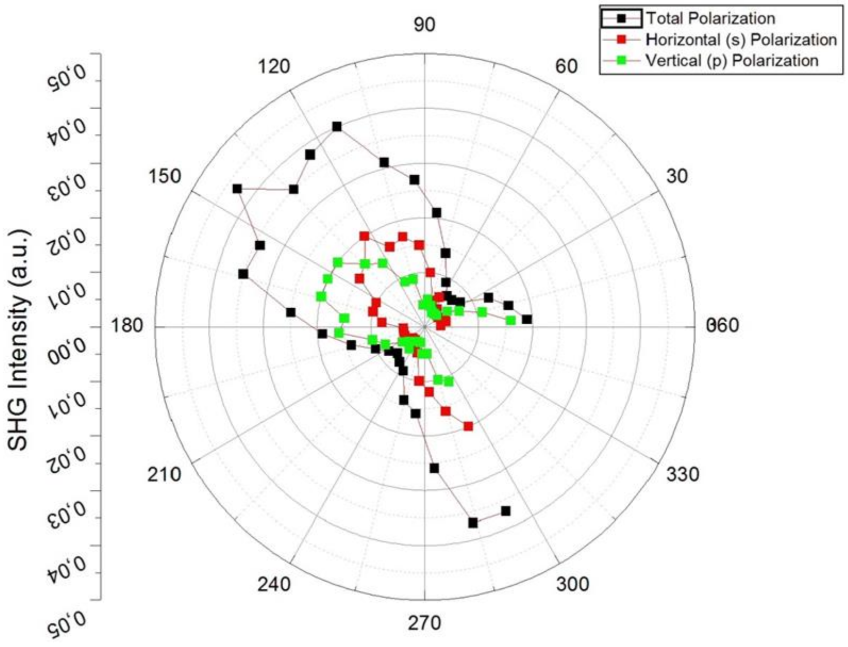

B

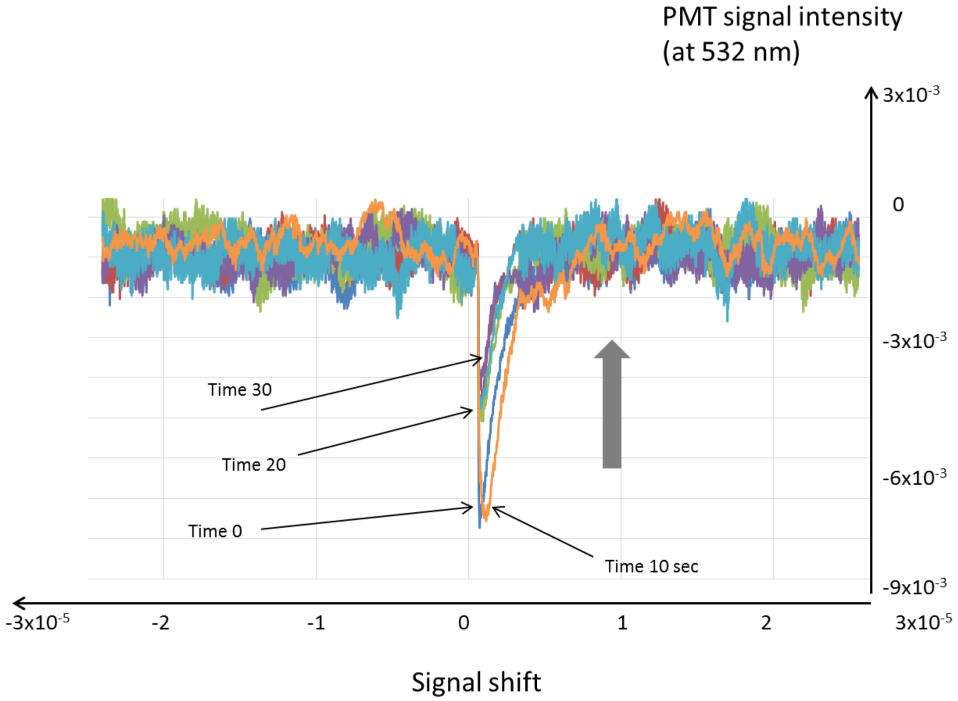

SM 4 **Chart 1**

We observed a ***photosalient effect*** in all studied single crystals of new non-centrosymmetric solids. It is a new phenomenon some details of which were first aired and classified in *Angewandte Chemie International Edition* (then in Photonics.com, June 2014), and in brief presented in diagram below.

It was first observed in crystals of Werner-type complex $[Co(NH_3)_3(NO_2)]Cl(NO_3)$, where single crystals underwent sudden jumps over distances 10^2 to 10^5 times their own size.

It was noted five ways in which crystals of a cobalt complex react to UV radiation:

- Some split into two or three pieces of similar size and zoom apart (**Mode 1**),
- some split into pieces of unequal sizes and move apart (**Mode 2**),
- some explode into several pieces (**Mode 3**),
- some move without any apparent splitting (**Mode 4**),
- some flip over several times, landing in the vicinity of their original position (**Mode 5**).

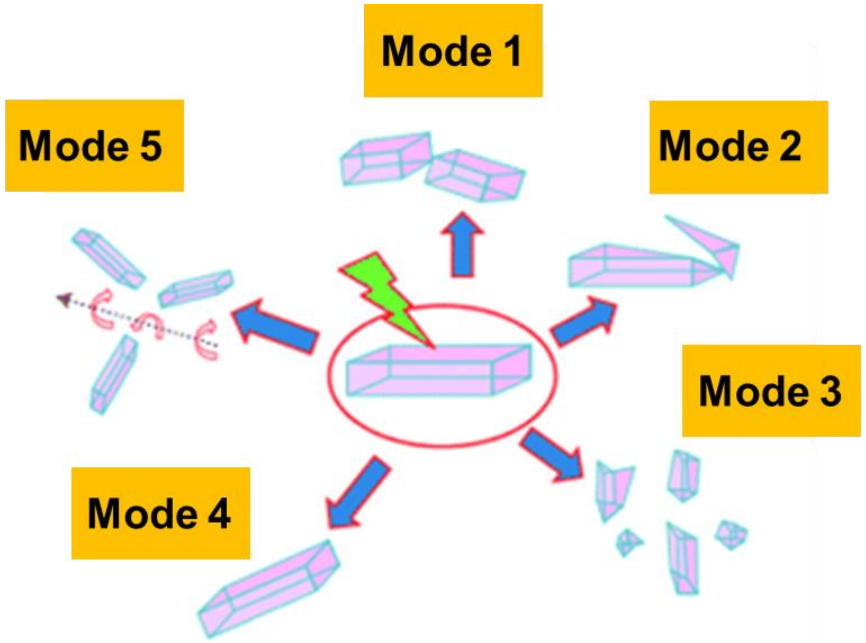

SM 5 **Figure 6**

The explanation for observed *mode 4 photosalient effect* in studied new mixed metal oxides. Compound **4** is used as an example.

Unit cell constants *b* and *c* in this orthorhombic cell are not changing much, while the shortest dimension *a* does change considerably. This is the distance between layers, or puckered plates, in the structure, which turned out to be sensitive to temperature.

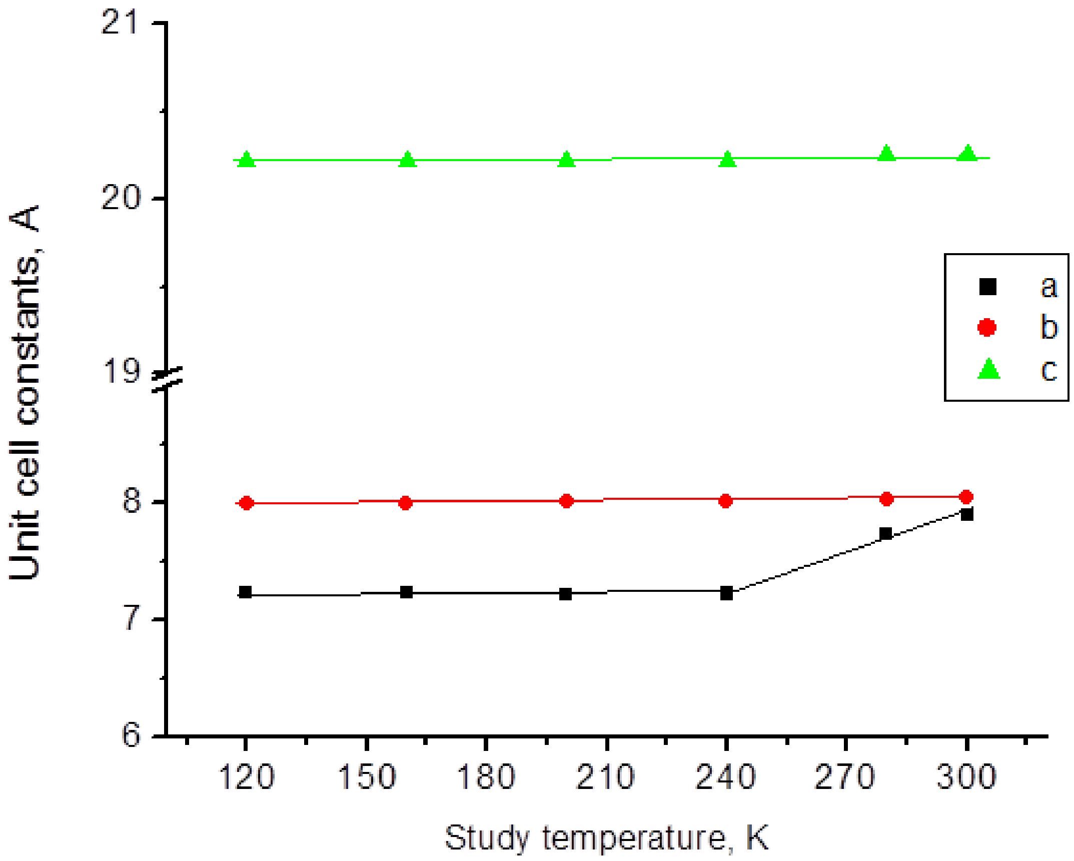

SM 6 **Table 1**

Key EPR active isotopes of the most common transition metals used in materials chemistry research and their EPR appearances. Used in this work elements are indicated with arrows.

Metal	Oxidation state	Isotopes	Spin (abundance)	# EPR lines
V	+4	51	7/2	8
Mn	+2	55	5/2	6
Fe	+3	54, 56, 57, 58	0, and 1/2 (~3%)	1 + 2 (1%)
Co	+2	59	7/2	8
Ni	+1, +3	58, 60, 61, 62, 64	0, and 3/2 (25%)	4
Cu	+2	63, 65	3/2	4
Mo*	+5	92, 94, ***95***, 96, ***97***, 98, 100	0, and 5/2 (25%)	1 + 6 (4%)
W**	+5	180, 182, ***183***, 184 186	0, and 1/2 (14%)	1 + 2 (7%)

*- isotropic spectrum consists of 7 signals;

**- isotropic spectrum consists of 3 signals

SM 7 **Figure 7**

Low temperature EPR spectrum of crystalline compound **3** showing overlapping signals. Shown coupled pentavalent molybdenum and tungsten centers.

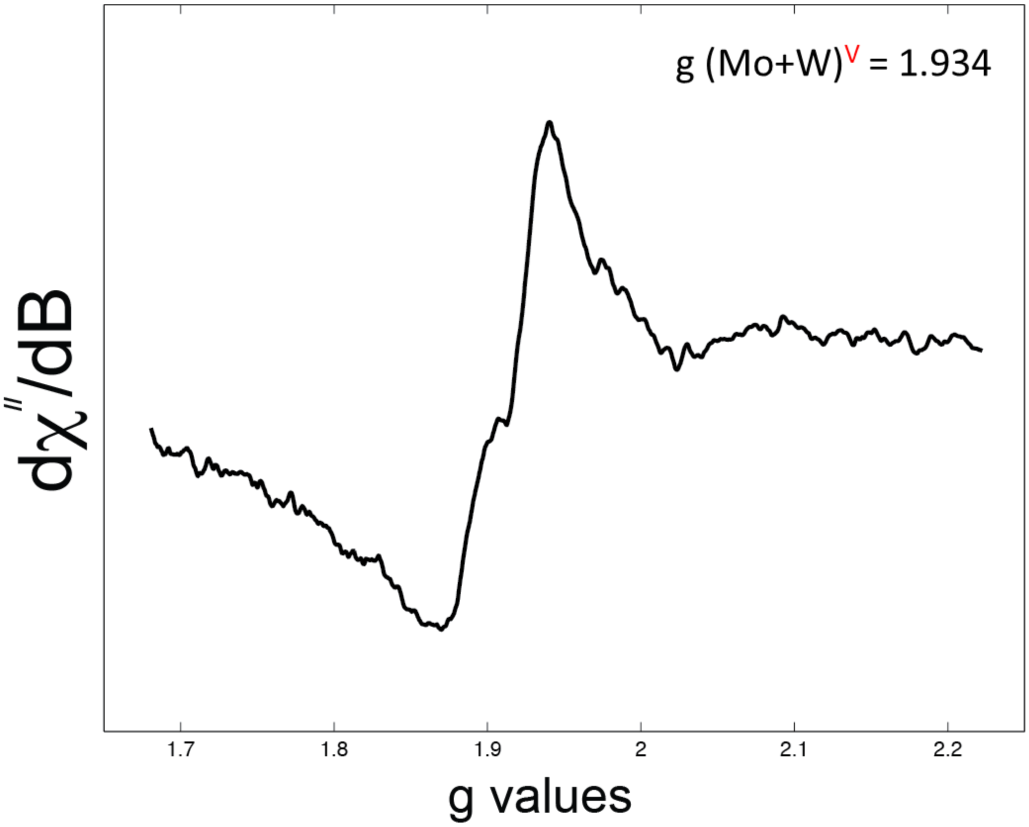

SM 8 Figure 8

Two different metal sites (marked as 1 and 2) in the structure of compound **4** (**a**), its EPR spectrum at 80K, and fitting (**b**), showing unresolved isotopic hyperfine coupling.

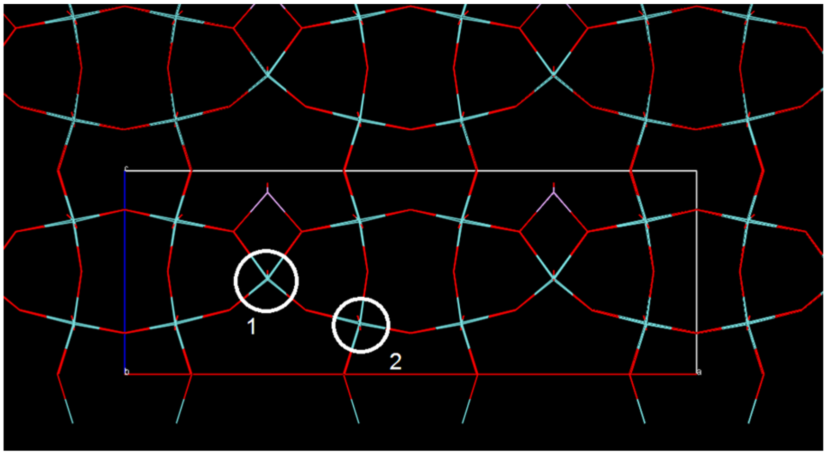

(a)

(b)

Site 1 (80%):
g_x = 1.955, g_y = 1.994, g_z = 2.022

Site 2 (20%):
g_x = 1.970, g_y = 1.975, g_z = 2.040

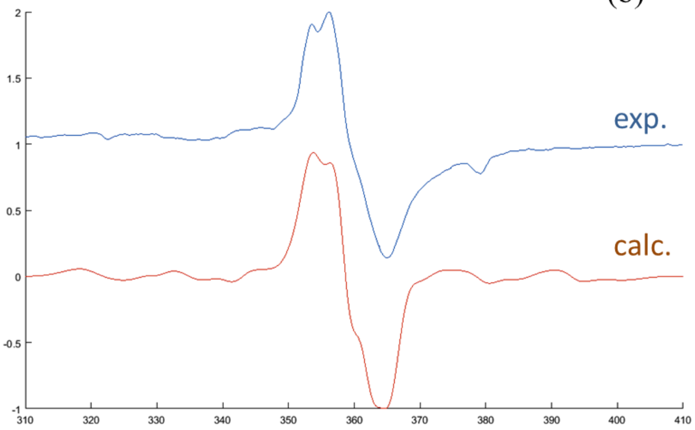

SM 9 **Figure 9**

Low temperature EPR spectra of crystalline compound **4** before and after recording its thermogramm under pure N_2 atmosphere. Blue spectrum contains unresolved hyperfine coupling of one unpaired electron of Mo(V) species with ^{95}Mo isotope nucleus.

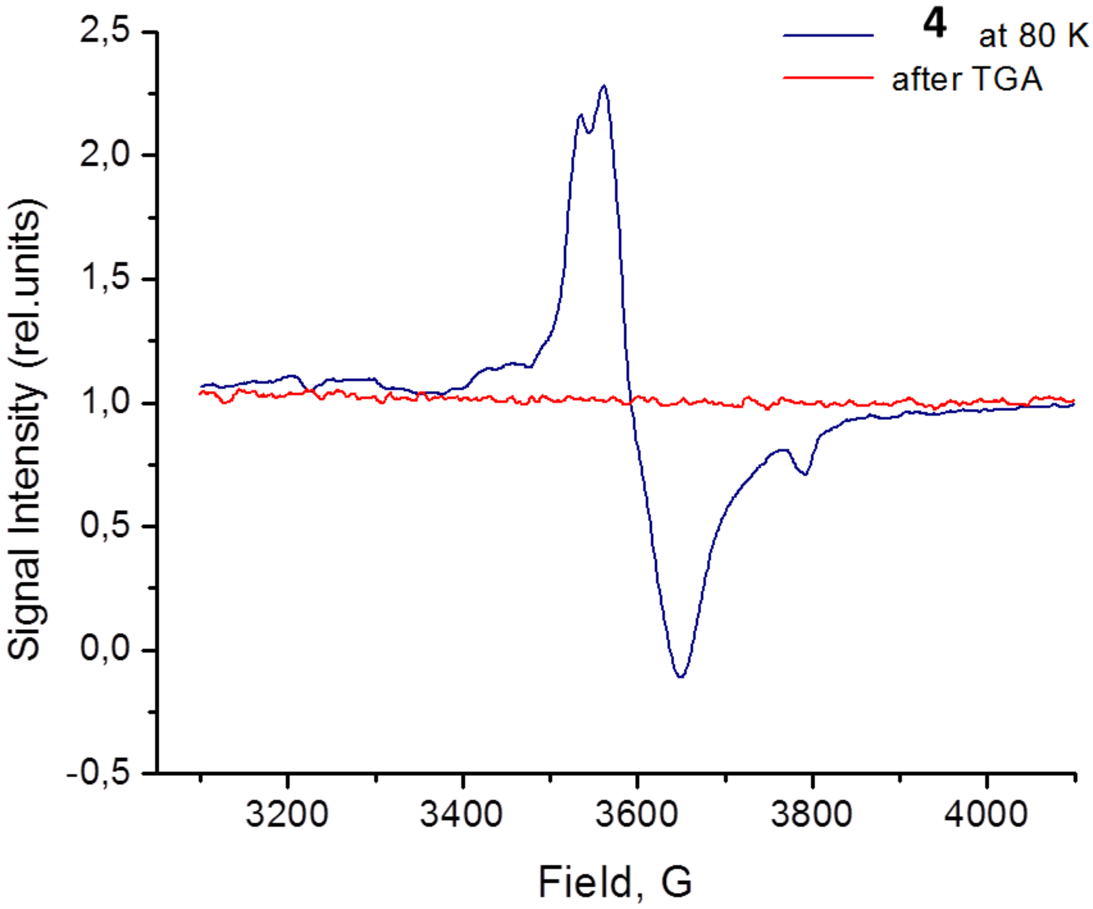

SM 10 **Table 2**

Table of standard redox potentials for elements involved in synthesis of mixed metal, mixed valence oxides.

Element	+6 to +5	+5 to +4	+6 to +4	+5 to +3	+3 to 0
**As**	n/a	n/a	n/a	+0.560 V *(ac)**	+0.240 V *(ac)*
				-0.67 V *(ba)***	-0.68 V *(ba)*
Nb	n/a	n/a	n/a	-0.10 V *(ac)*	-1.10 V *(ac)*
Mo	+0.50 V *(ac)*	+0.17 V *(ac)*	+0.646V *(ac)*	+0.085 *(ac)*	
	n/a	n/a	-0.780	n/a	
W	-0.029 *(ac)*	-0.031 *(ac)*	n/a	n/a	n/a
	n/a	n/a	-1.259 V *(ba)*	n/a	n/a

*- reported for solutions at acidic conditions (pH = 0)

**- reported for solutions at basic conditions (pH = 14)

All values from John Emsley “The Elements”, 2nd Ed., Clarendon Press-Oxford, 1991.

SM 11 Figures 10, 11

Micro-Raman spectra of crystals of new compounds showing usable area of M-O (M=Nb, Mo, W) and As(P)-O vibrations.

Compound 1:

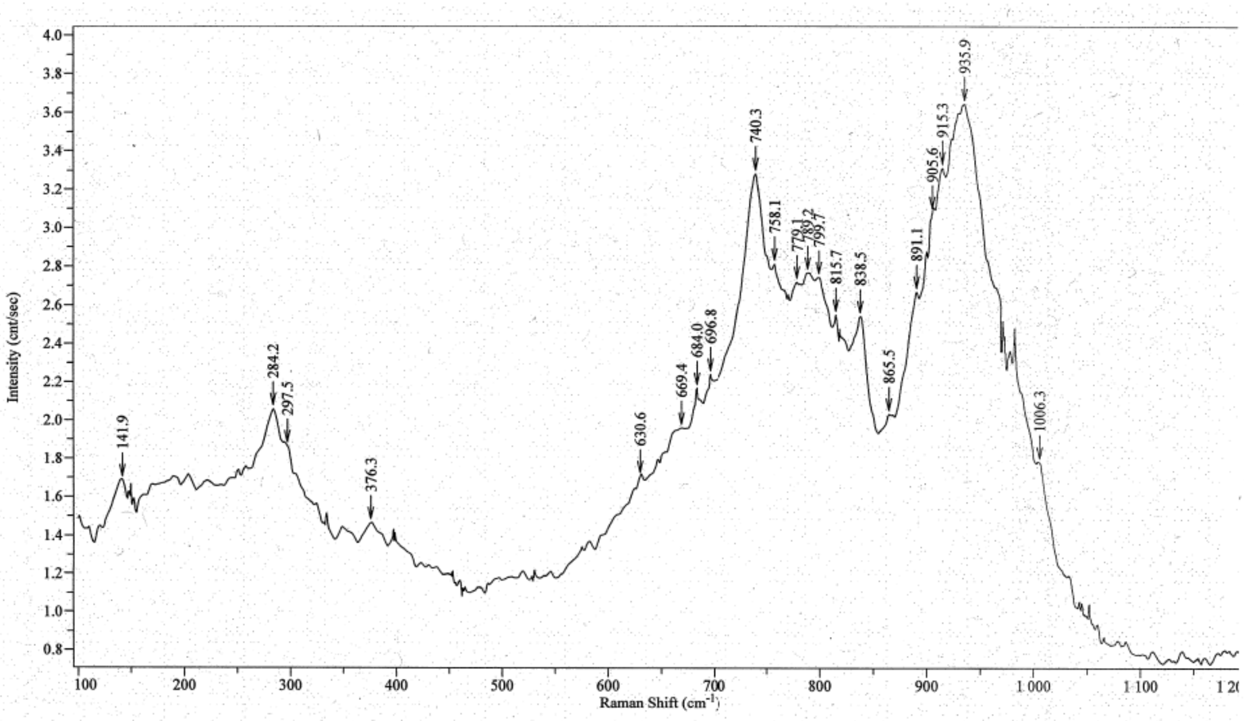

Compound 2:

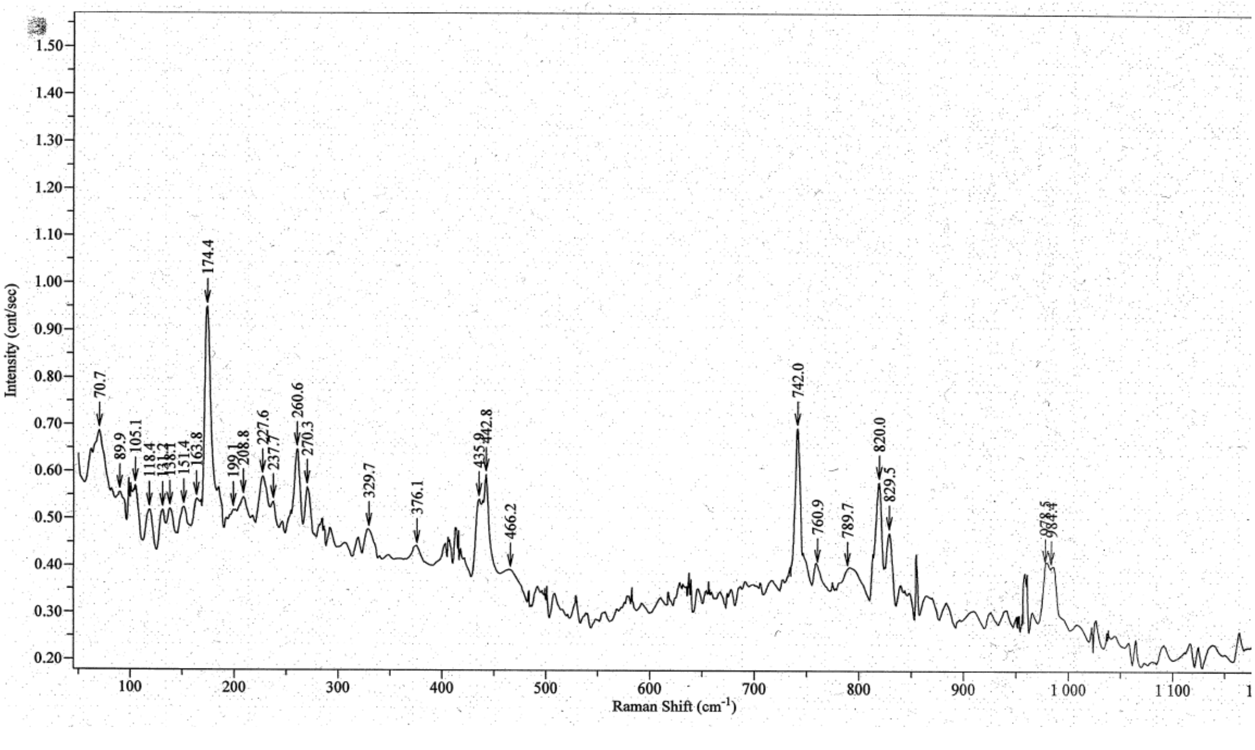

SM 12 **Figure 12, 13**

Micro-Raman spectra of crystals of new compounds showing usable area of M-O (M = Mo, W) vibrations.

Compound 3:

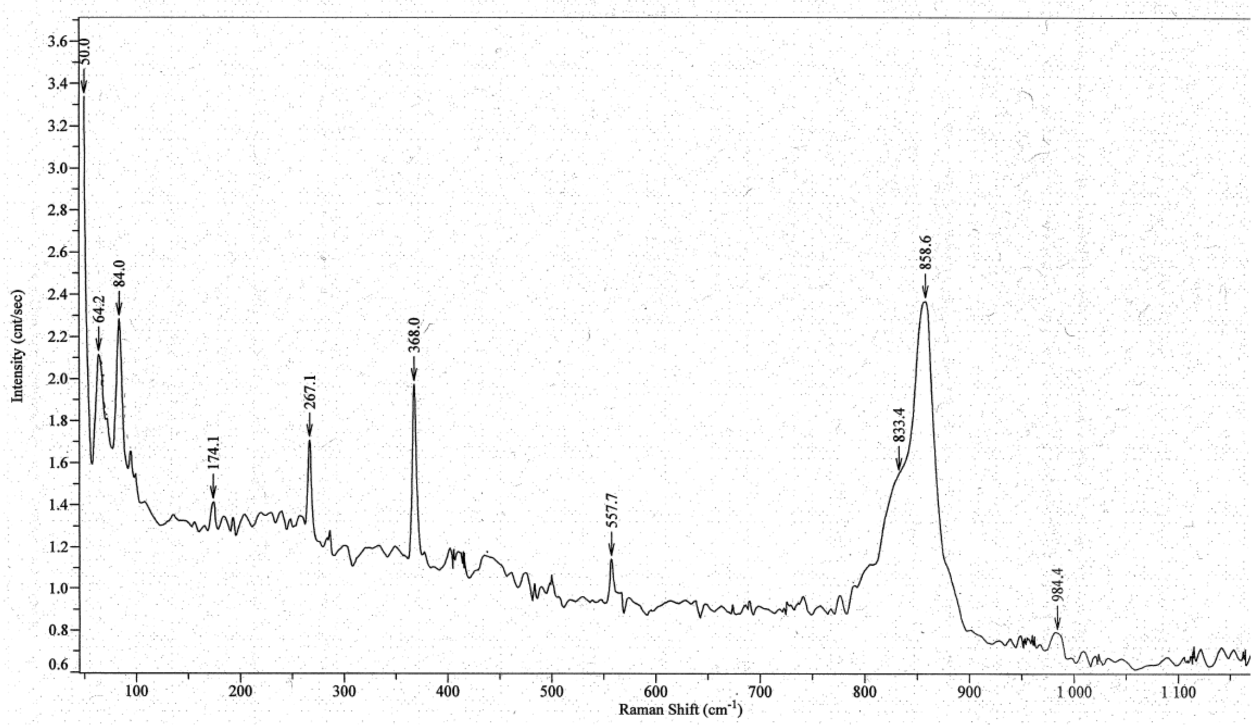

Compound 4:

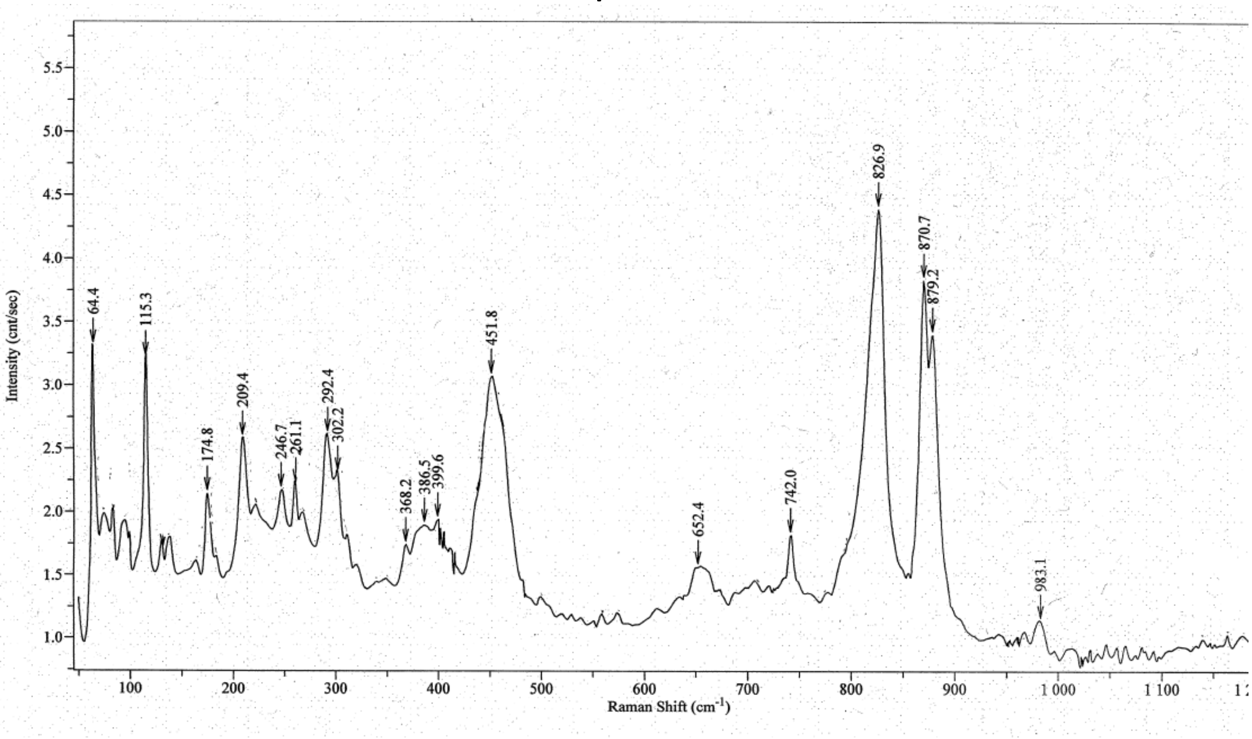

SM 13 Figure 14

Full line shape analysis for solid state reflectance spectrum of compound **2** showing deconvoluted peaks and overall fitting statistics. The minimum number of five Gaussian-type peaks is necessary to fit well experimental curve (black). Red trace is the sum of theoretical fit, while blue peaks represent individual components of spectral envelope.

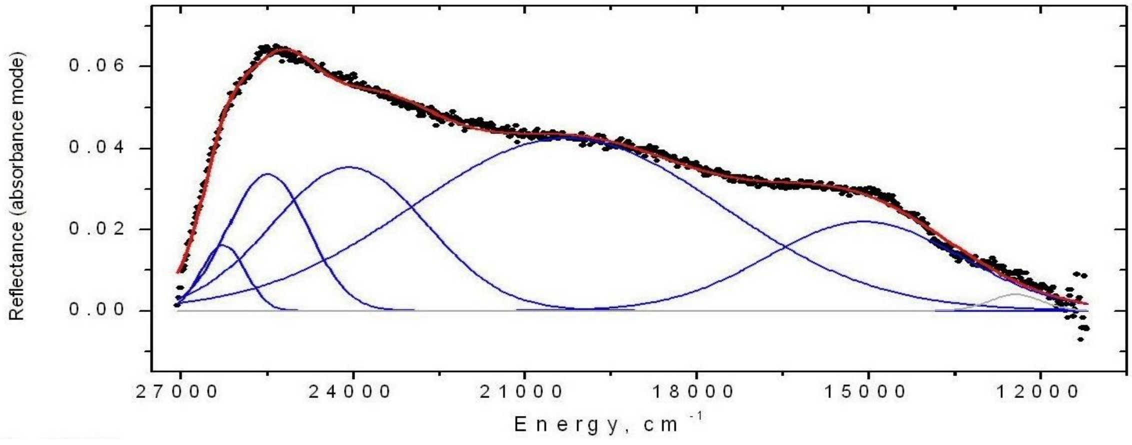

χ^2 = 1.92 10^{-6} COD = 0.993 number of used data points (from spectrum)= 761; degrees of freedom in the system = 746

Students' statistics = 0.00143 Coefficient of correlation R = 0.996

Fitting Results

Peak #	Peak Type	AreaFitT	FWHM	MaxHeight	CenterGrvty	AreaFitTP
1	Gaussian	15.16	897.7	0.016	26259.7	2.65
2	Gaussian	58.30	1643.1	0.034	25483.6	10.22
3	Gaussian	117.75	3170.4	0.035	24066.2	20.64
4	Gaussian	286.97	6374.5	0.043	20258.6	50.31
5	Gaussian	92.27	3998.2	0.022	15073.8	16.18
		570.44				

SM 14 **Figures 15, 16**

Precession images from single crystal diffraction studies of compound **1**: **top** – kl1 plane (14), **bottom** – h1l plane (15). Both clearly indicate aperiodic nature of studied specimen.

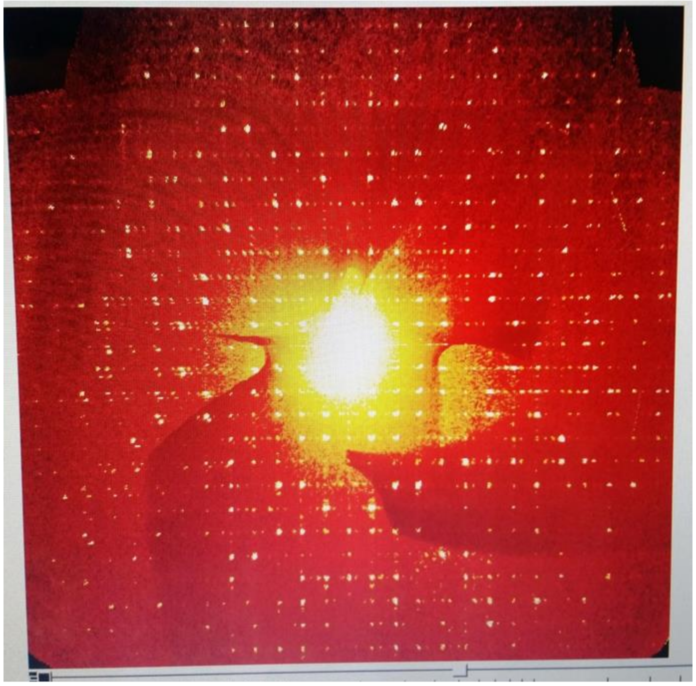

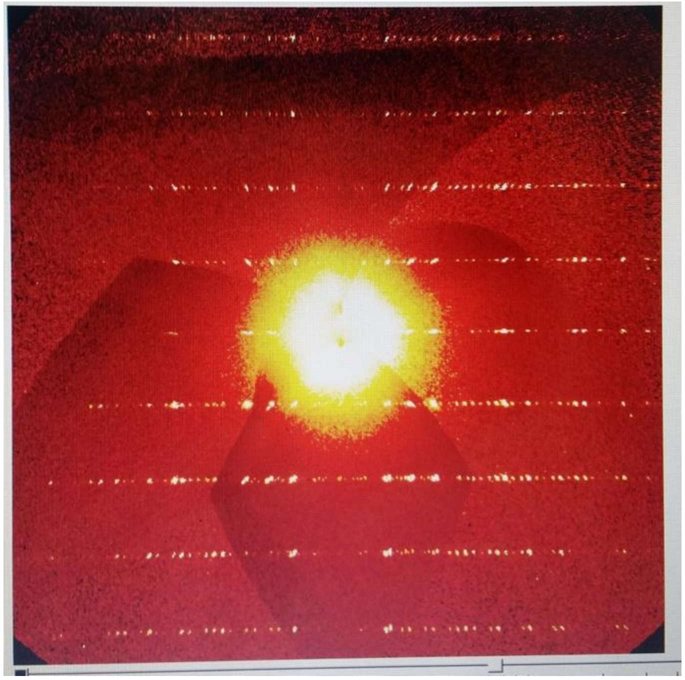

SM 15 **Figure 17, 18**

RLAT (reciprocal lattice view) image of all harvested reflections from 1464 frames recorded during single crystal diffraction experiment from a sample of compound **1**. For structure solution and refinement the biggest, central cluster of reflections has been selected (indicated by red circle).

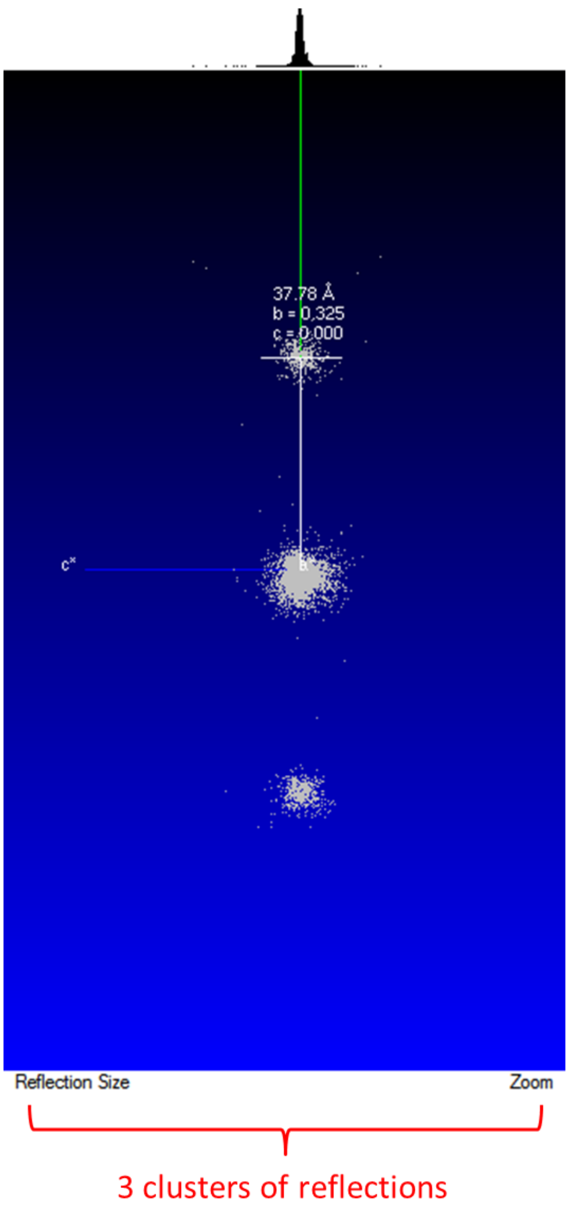

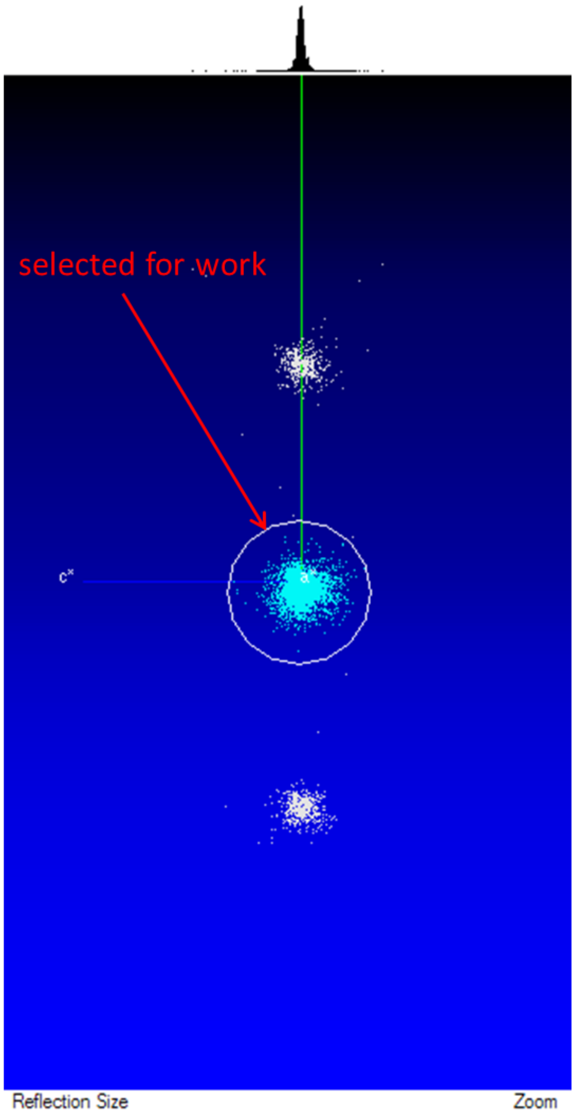

SM 16 **Figure 19**

Prospective view of the unit cell content using polyhedrons' representation of the crystal structure of compound **1**.

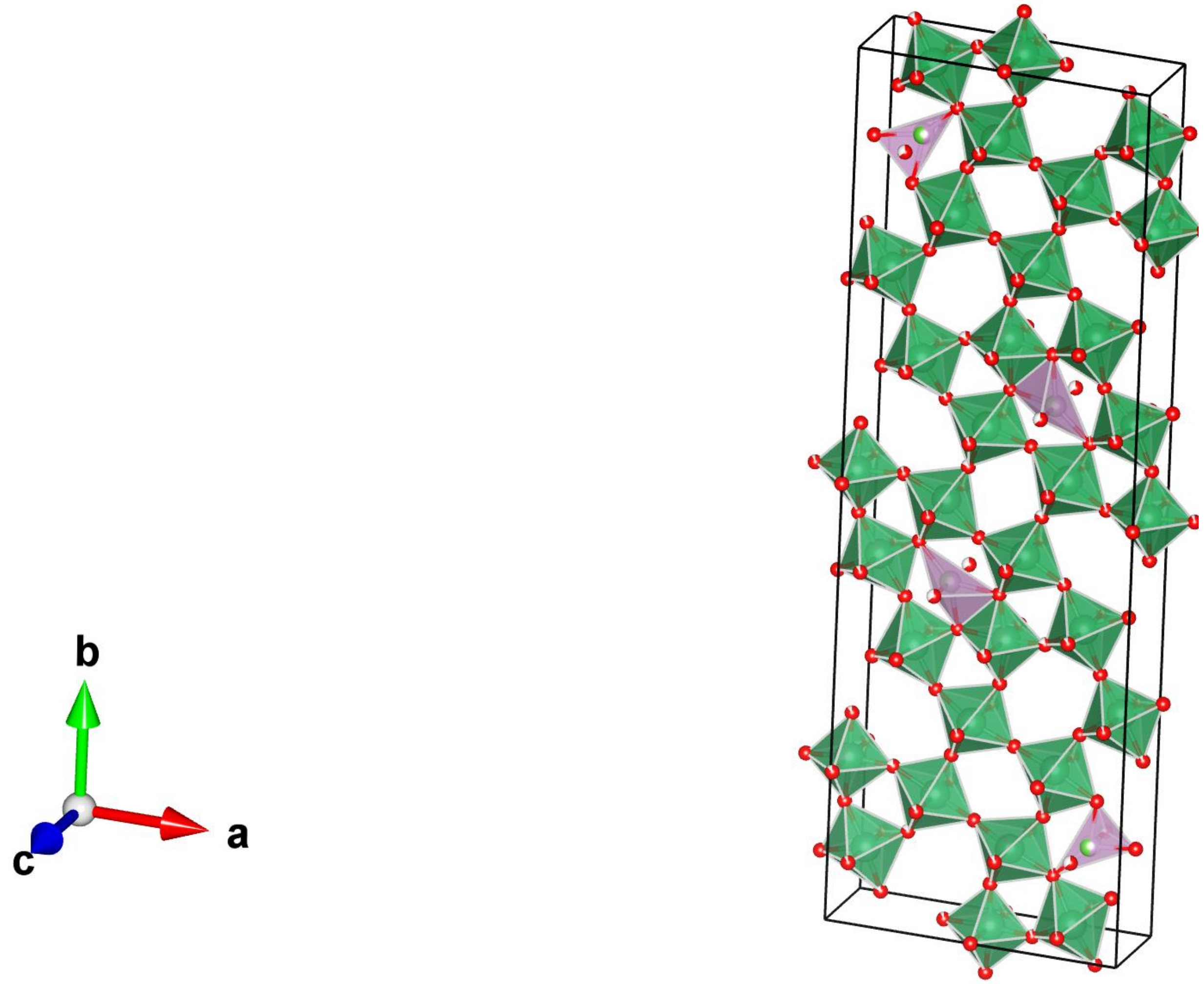

SM 17 Figure 20

Two orthogonal views of the GROW fragment in the structure of **1**. Green arrows show some corner-sharing octahedrons in the structure, while yellow arrows show edge-sharing places for junction.

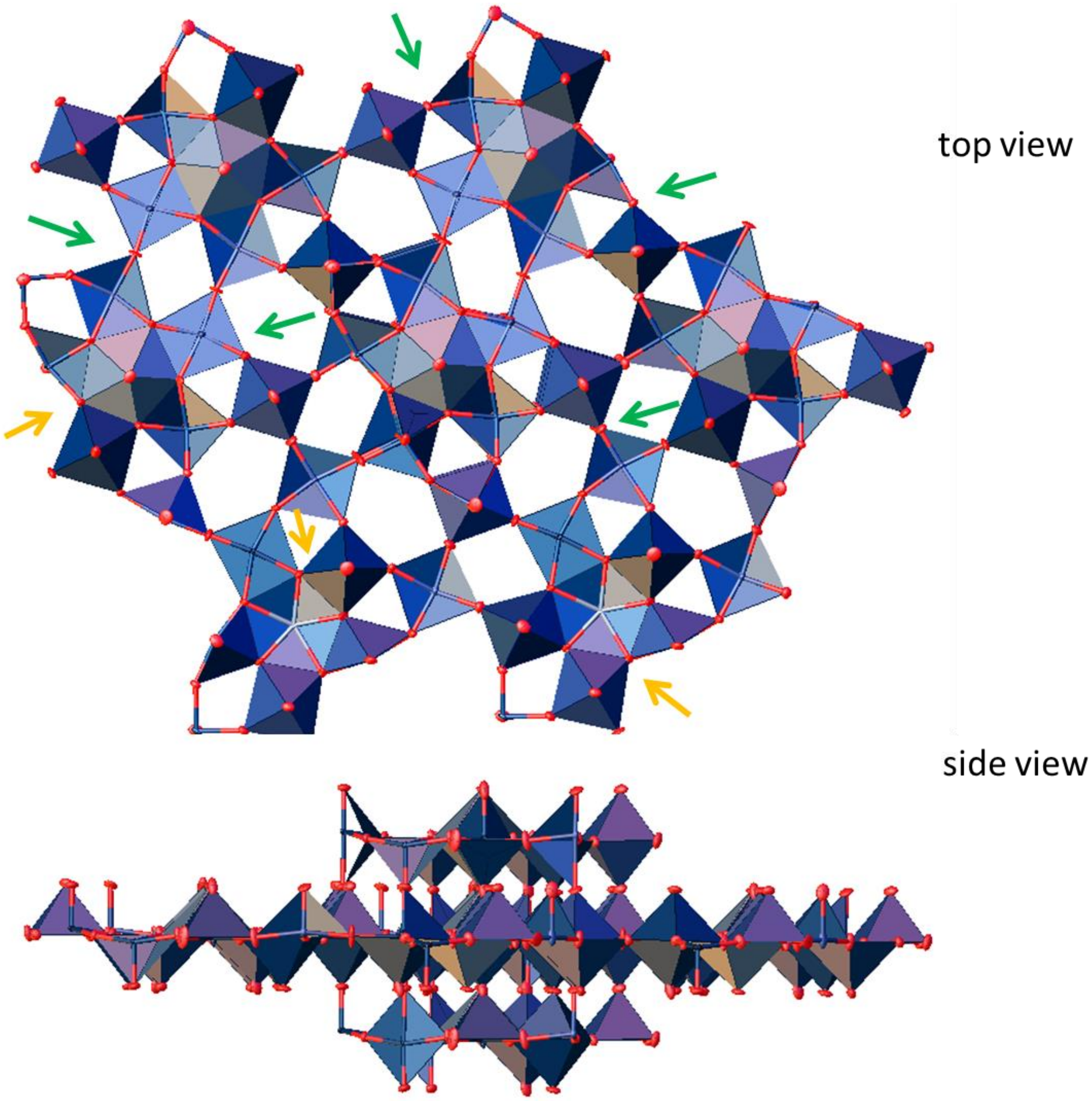

SM 18 **Figure 21**

Metallocycles in the structure of **1**: analysis of planarity of the core. A – top view, B – side view, C – dihedral angle in eight-membered cycle in the middle of the structure where 2-fold rotation axis is.

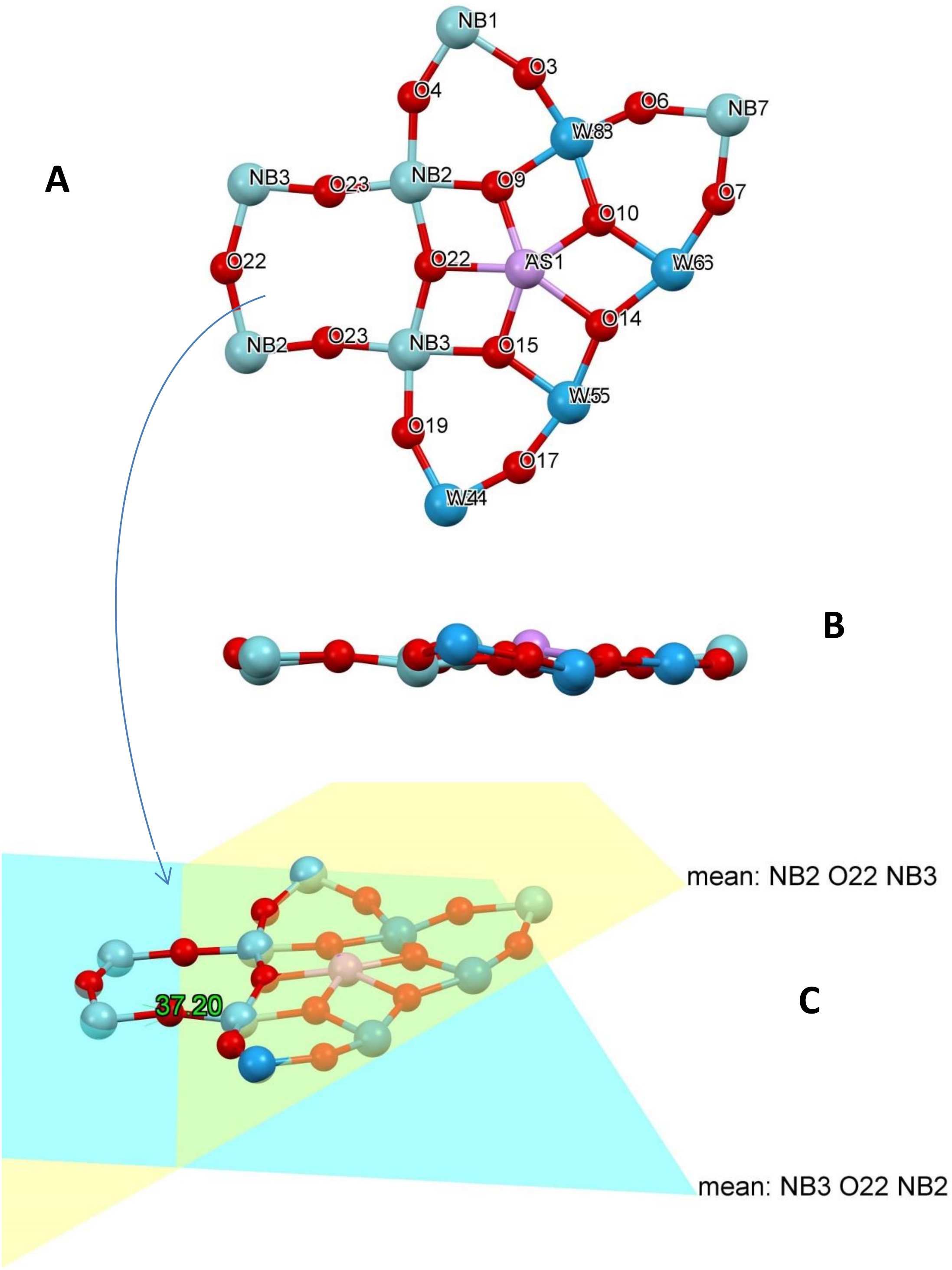

SM 19 Figure 22

Metallocycles in the structure of **1**: analysis of planarity of three six-membered cycles in the core.

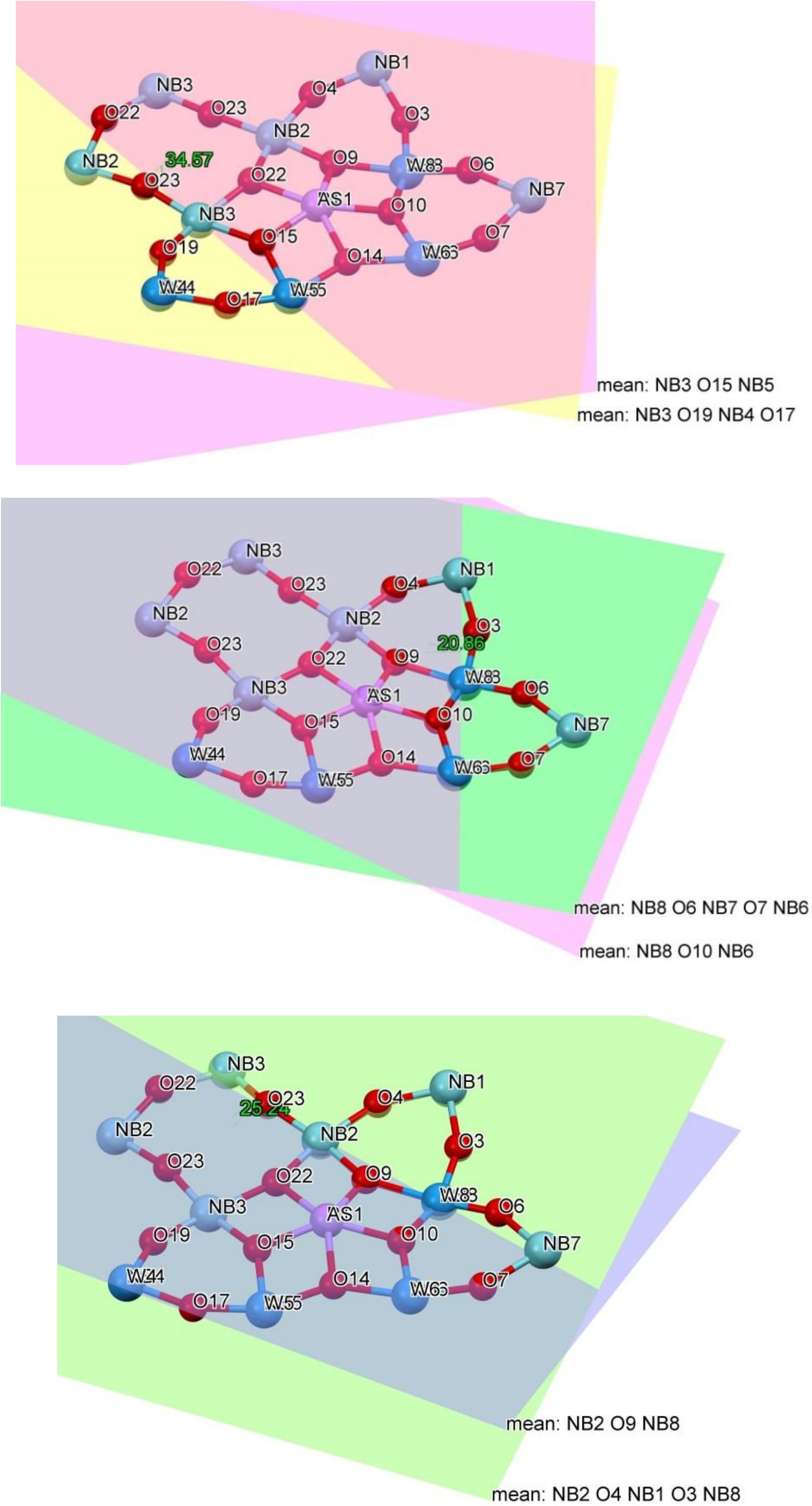

SM 20 **Figure 23**

Side (**A**) and top (**B**) views of pnictogens centers in the structure of **1** showing highly distorted trigonal bipyramidal geometry.

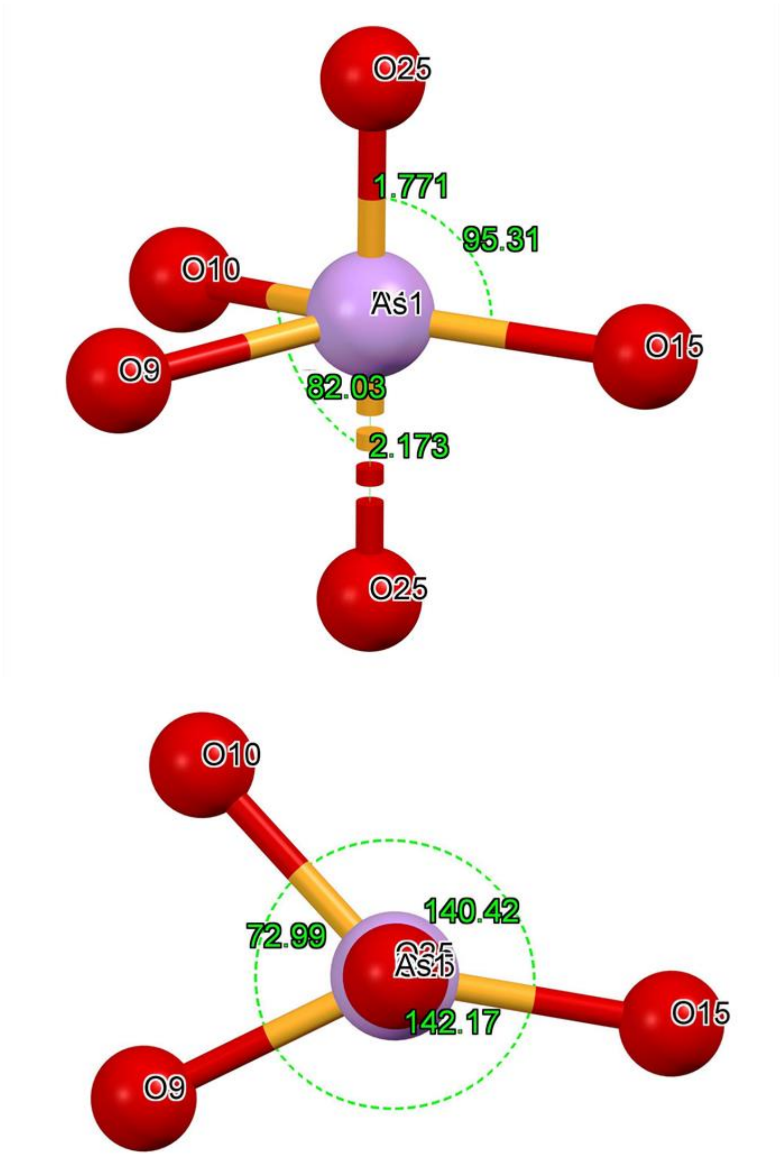

SM 21 **Figure 24**

Geometry of a pentagonal star shaped formation in the structure of **1** angles between pnictogen atoms As/P and oxygen atoms – side view **A** and top view **B**, and bond lengths C. Shared sites between transition metals and pnictogens are indicated with green arrows.

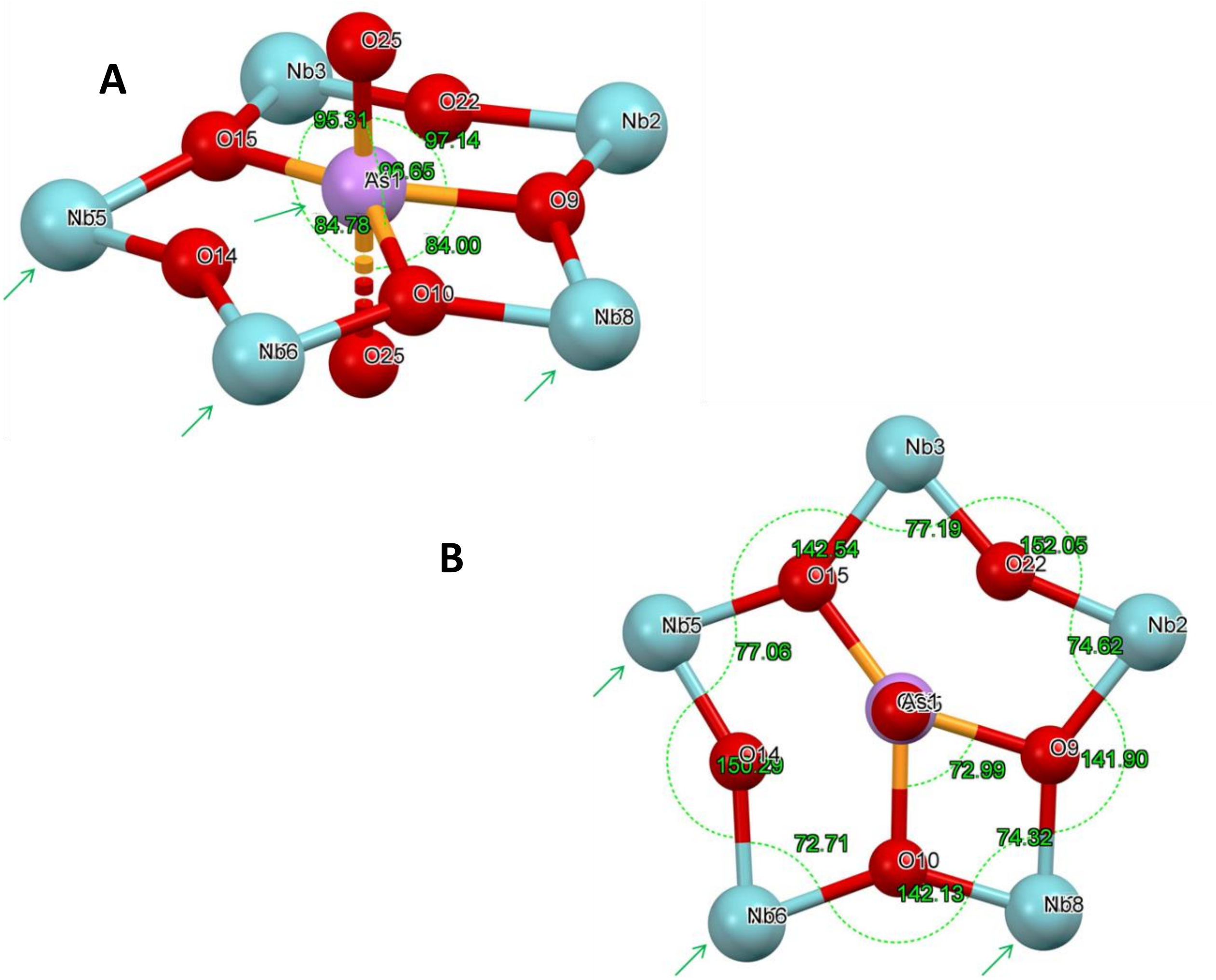

SM 22 **Figure 25**

Geometry of a pentagonal star shaped formation in the structure of **1** angles between pnictogen atoms showing bond lengths.

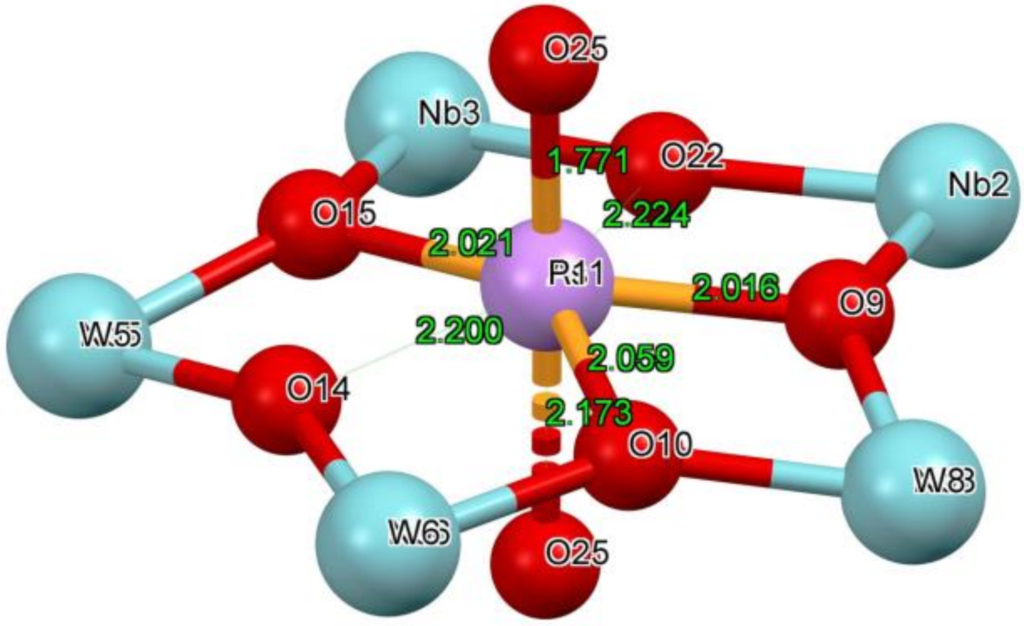

SM 23 Figure 26

Analysis of structure of **1**: values of principal bonds and angles at individual metal centers with SOF = 1, Nb1 and Nb2

top view

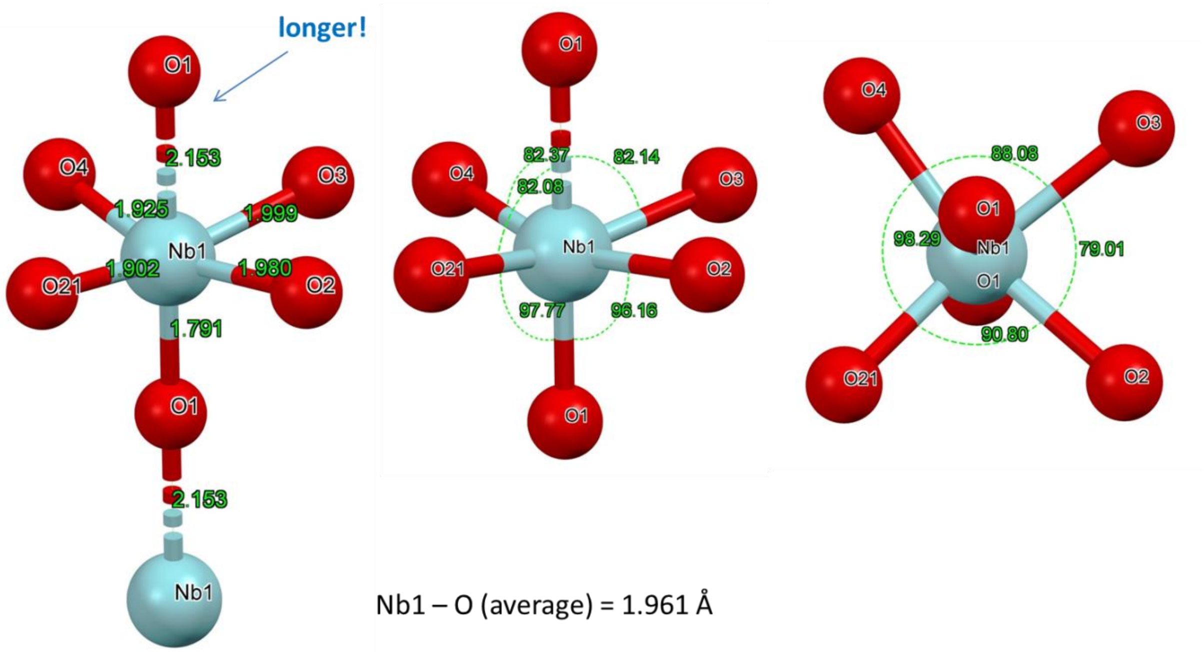

Nb1 – O (average) = 1.961 Å

top view

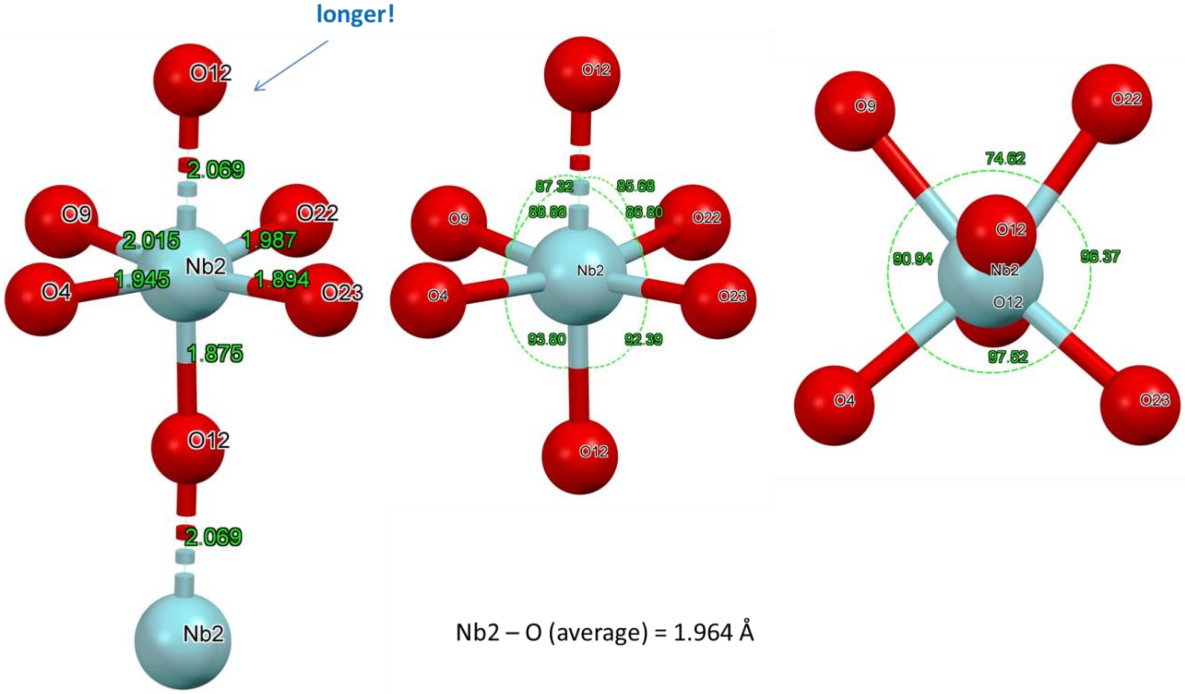

Nb2 – O (average) = 1.964 Å

SM 24 **Figure 27**

Analysis of structure of **1**: values of principal bonds and angles at individual metal centers with SOF = 1, Nb3, Nb7.

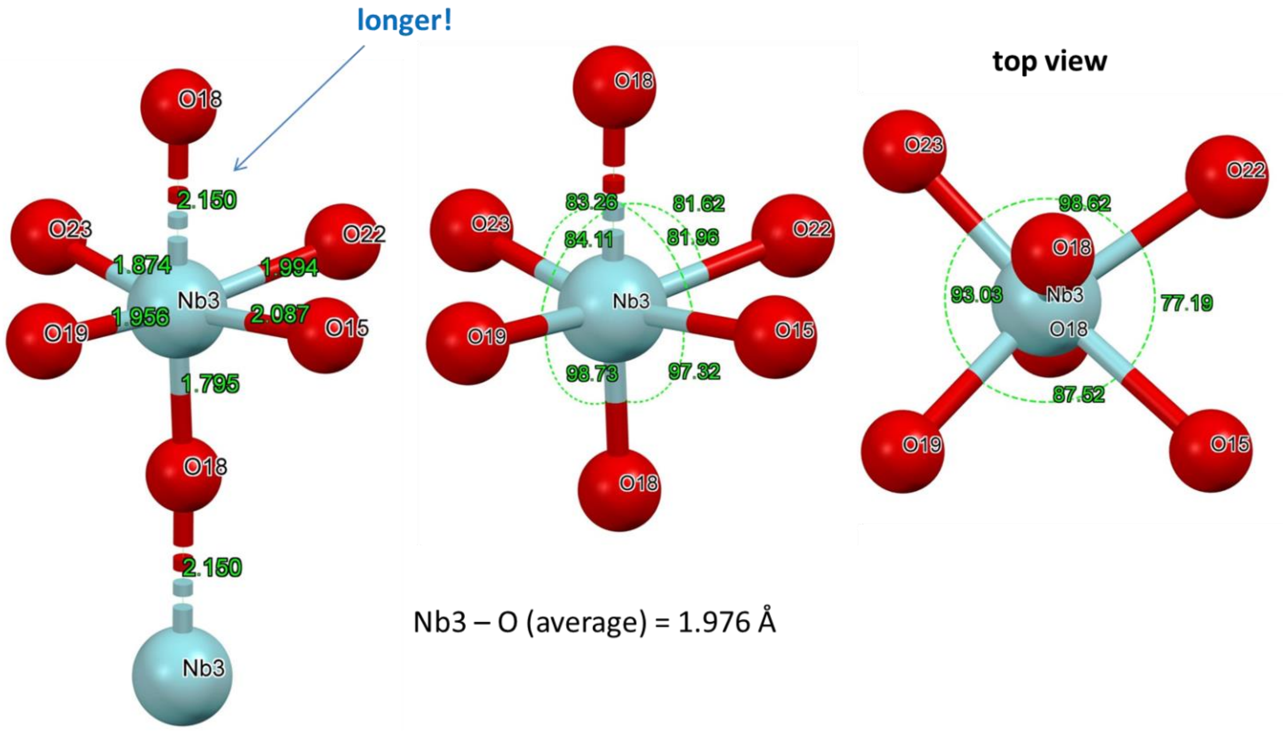

Nb3 – O (average) = 1.976 Å

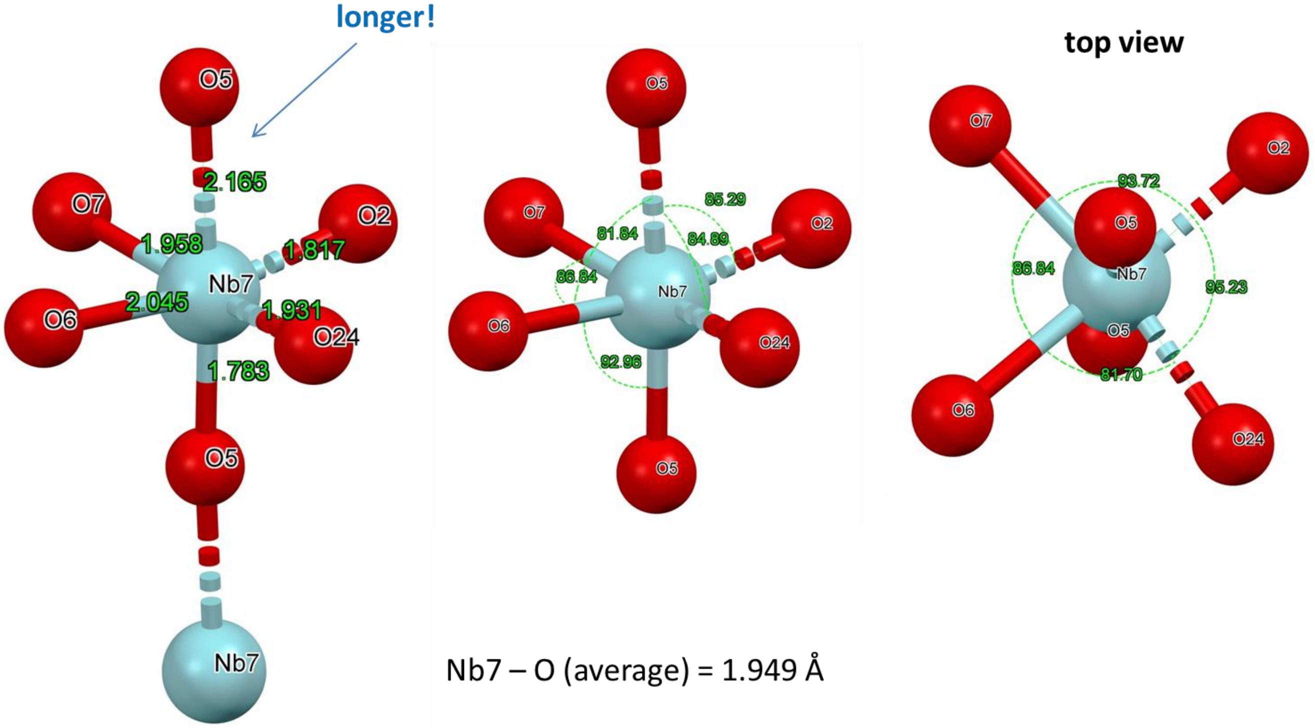

Nb7 – O (average) = 1.949 Å

SM 25 **Figure 28**

<u>Literature data analysis of polyhedrons distortions</u>: selected bond lengths and valence angle in the structure of N_2O_5 (monoclinic, P1) for comparison with the structure of **1**. Non-symmetry related metal centers Nb2 and Nb3 were chosen. A spread of Nb–O bond distances is ranging from 1.81–2.34 Å (variance of ~22.6% value). Same for angles: 73.61^o to 105.92^o representing ~30% value). Both data are from reference [1] (see the *<u>Reference Section</u>* at the end).

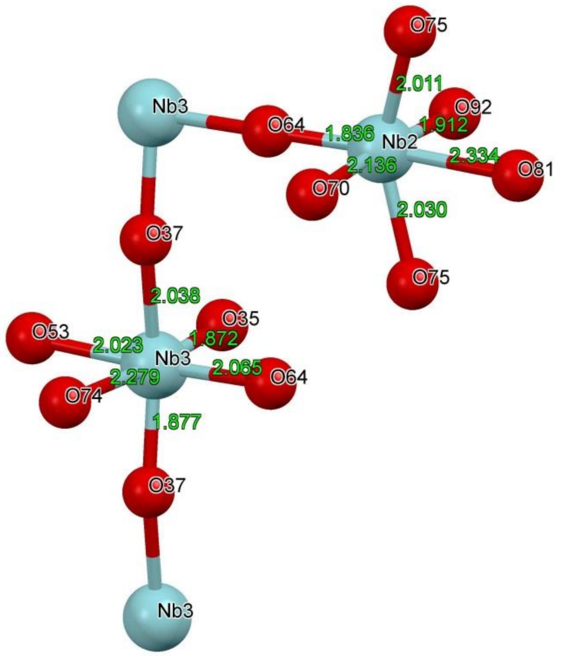

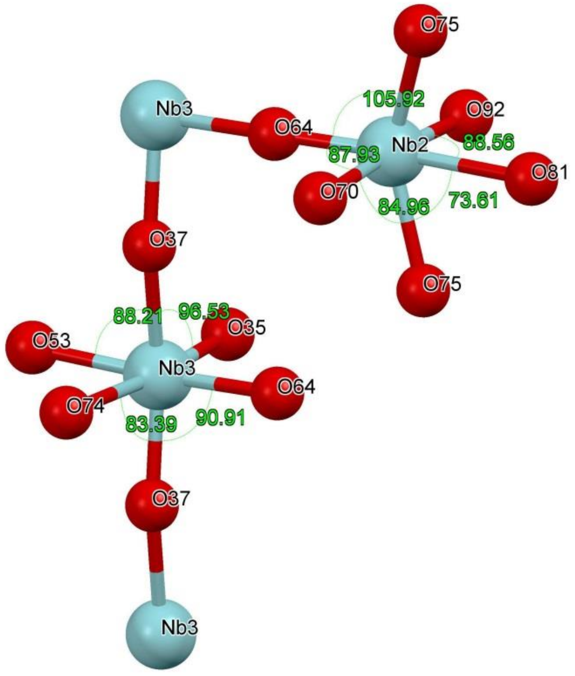

SM 26 Figure 29

<u>Literature data analysis of polyhedrons distortions</u>: selected bond lengths and valence angle in the structure of WO_3 (monoclinic, $P2_1/c$) for comparison with same parameters in the structure of **1**. Non-symmetry related metal centers W2 and W3 were chosen. A spread of W–O bond distances ranging from 1.841–2.065 Å, ~11% of value (from reference [2]). In another also monoclinic polymorph spread of W-O bonds is from 1.855 to 2.034 Å, ~8.8% of value. At the same time valence angles O-W-O range from 84.62^o to 95.37^o (~11.3% value) (from reference [3]) (see the Reference Section at the end).

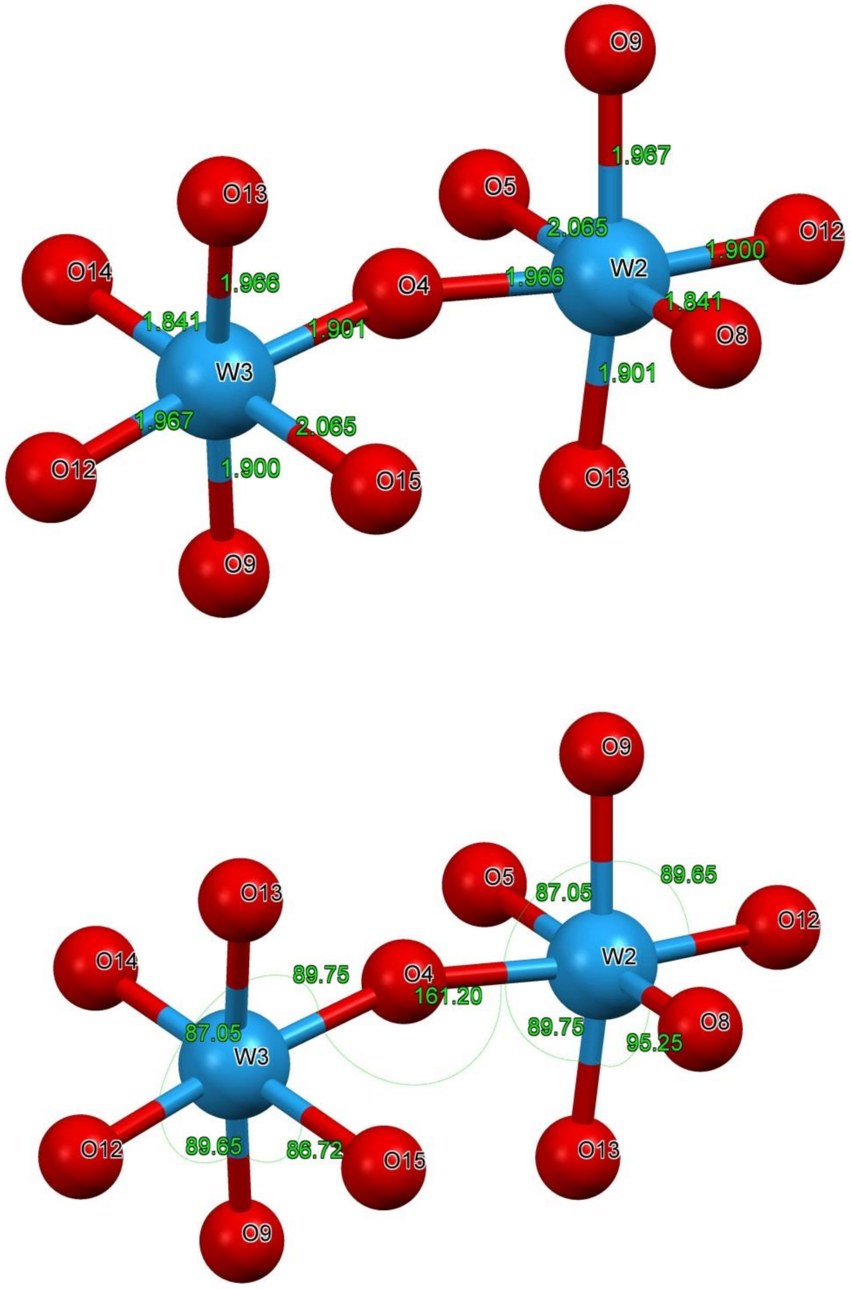

SM 27 **Chart 2**

Organization of 2D crystal lattice in the structure of compound **1**: thick plates are stacked on a top of other with different averaged E-O distances (E = As, P, Mo, W) in between forming layered structure. Each plate represents puckered 2D net.

The averaged distance inside the ASU net based on 17 data (blue plate), with upper pink plate based on 11 data, and lover brown plate based on 8 data as generated by the Mercury Crystallographic Viewing software.

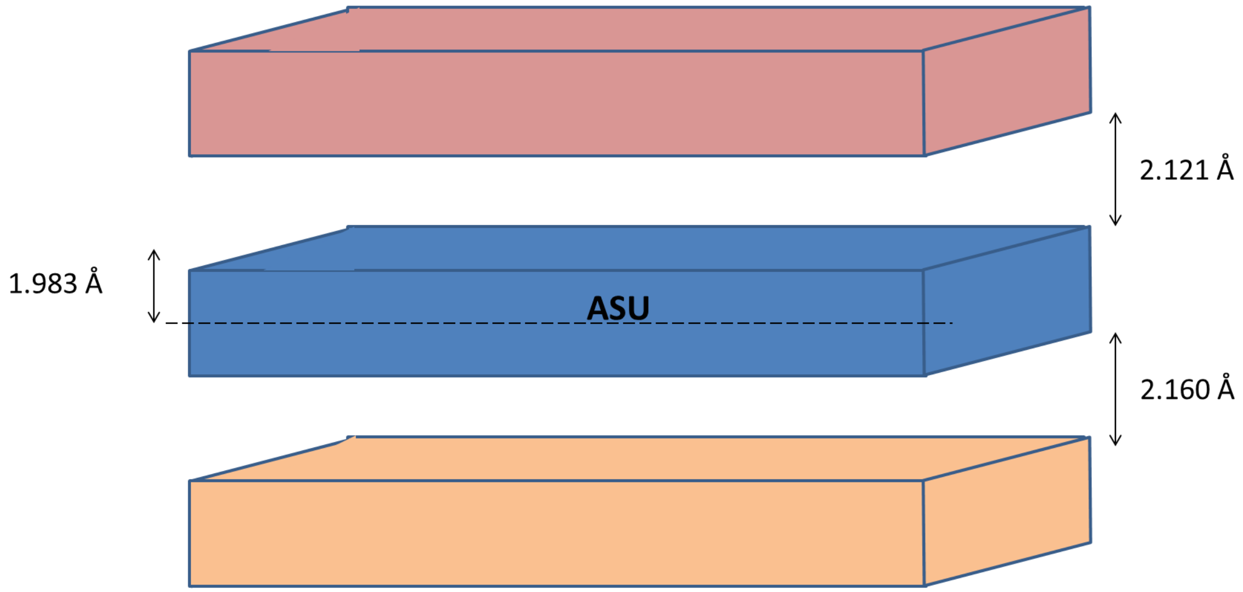

SM 28 **Figure 30**

The videomicroscope scaling of the needle-type specimen of compound **2** used for crystallographic studies. Indexing crystal faces led to accurate dimensions determinations that were used in SADABS procedure for absorption correction.

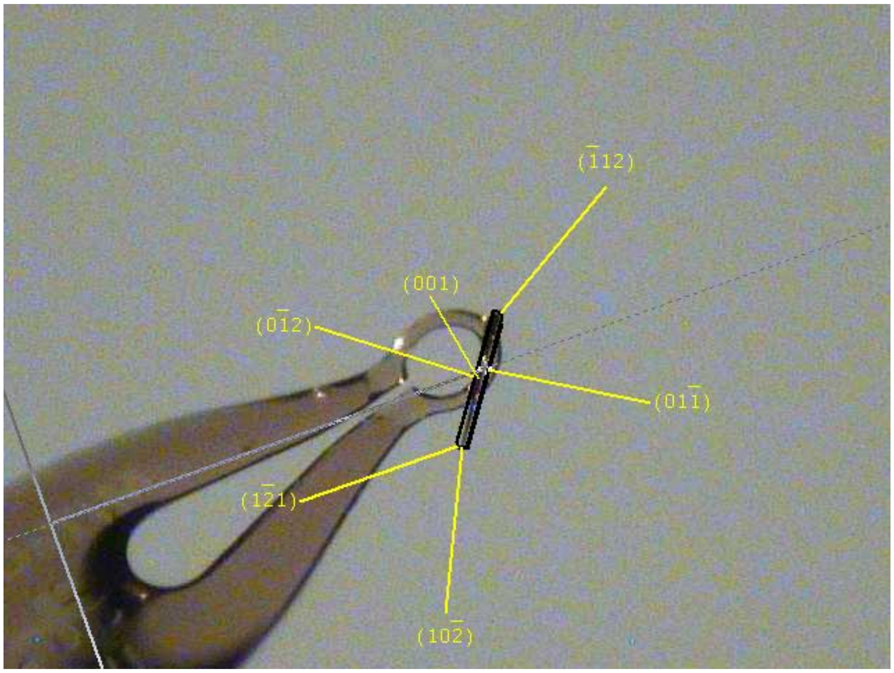

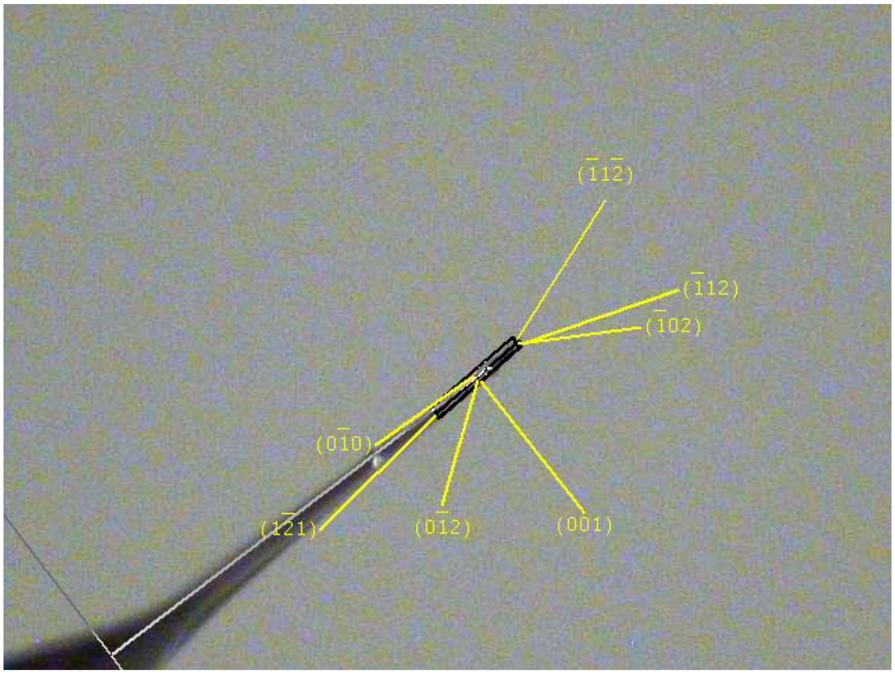

SM 29 **Figure 31**

Polyhedral representation of the structure of **2**: view along *b*-direction. Oxygen atoms of occlusion are indicated in red.

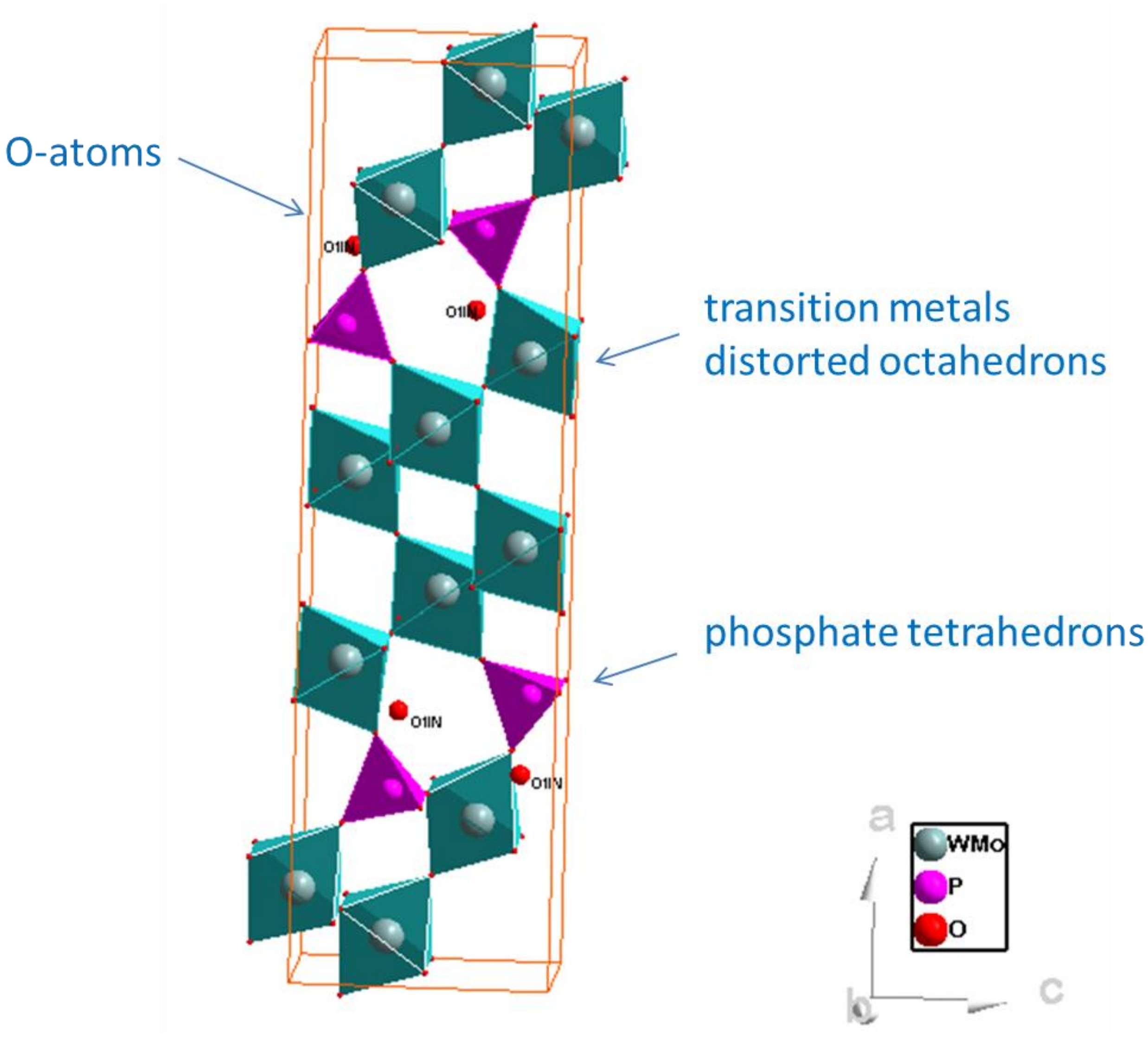

SM 30 **Figure 32**

Two orthogonal views of the GROW fragment in the structure of **2**. Corner-sharing octahedrons are clearly seen in the structure, while yellow arrows show corner-sharing tetrahedrons of PO_4-fragment for junction.

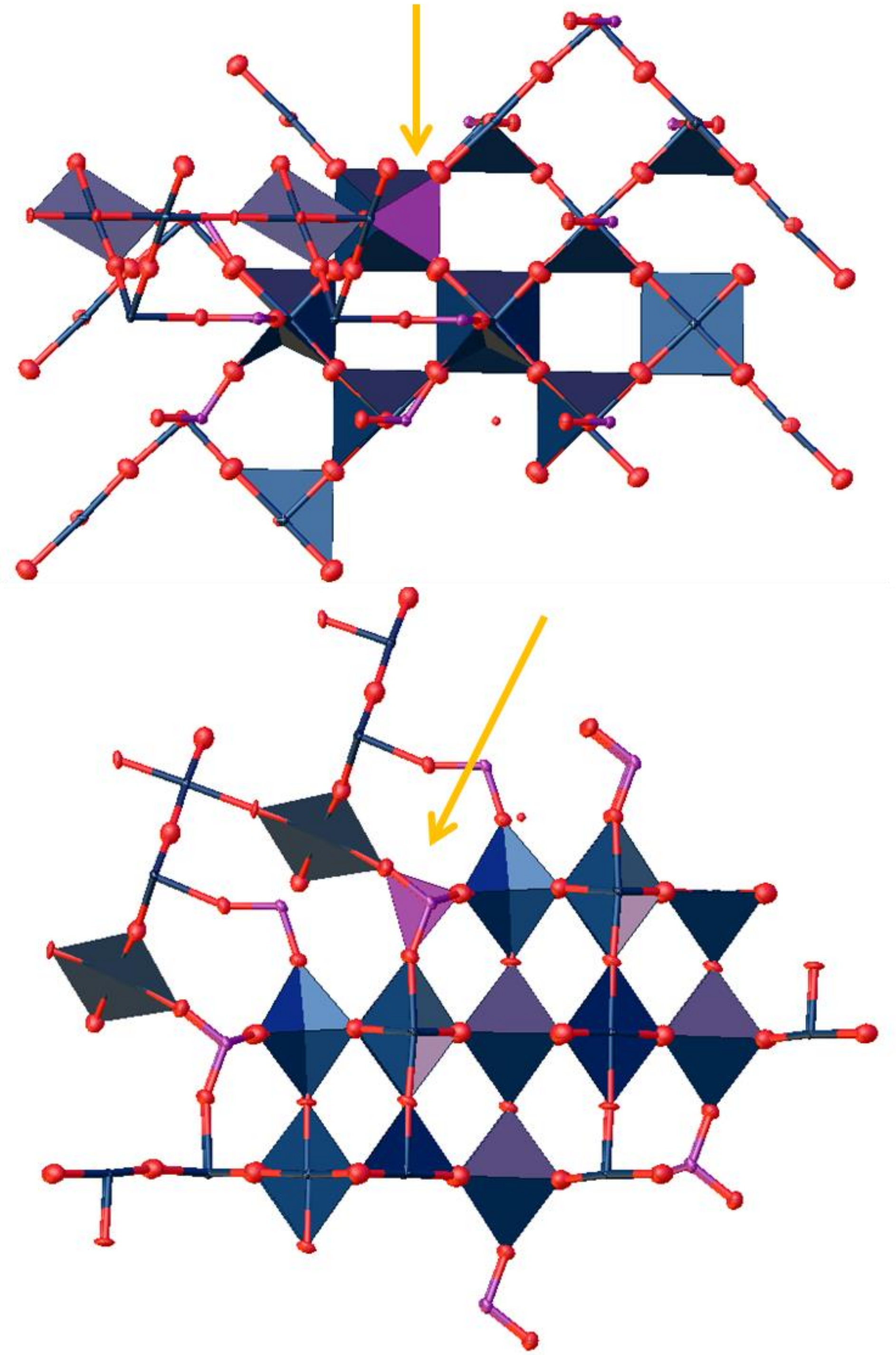

SM 31 **Figure 33**

Analysis of structure of **2**: values of principal bonds and angles at individual metal centers.

Mo/W – O bonds average = 1.900 Å

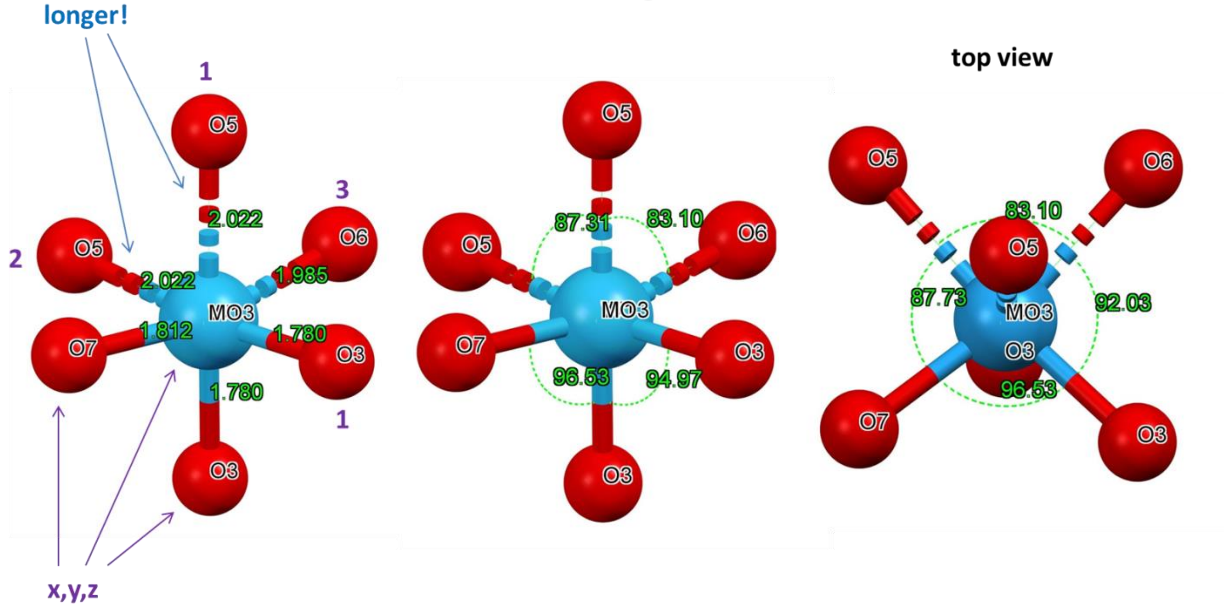

symmetry codes:
1 = ½-x, -y, -½ +z 2 = x, -½-y, -1+z 3 = ½-x, -y, -½+z

Mo2/W2 – O bonds average = 1.915 Å

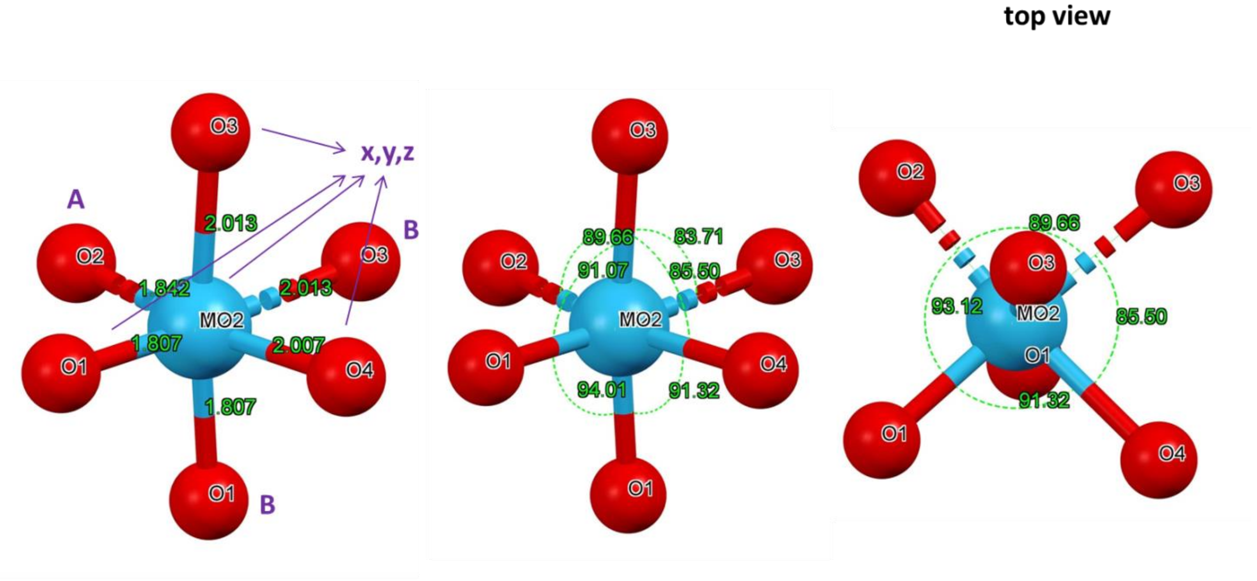

symmetry codes:
A = -x, -½ +y, -z B = x, -½-y, z

SM 32 **Figure 34**

Analysis of structure of **2**: values of principal bonds and angles at individual metal centers (continued).

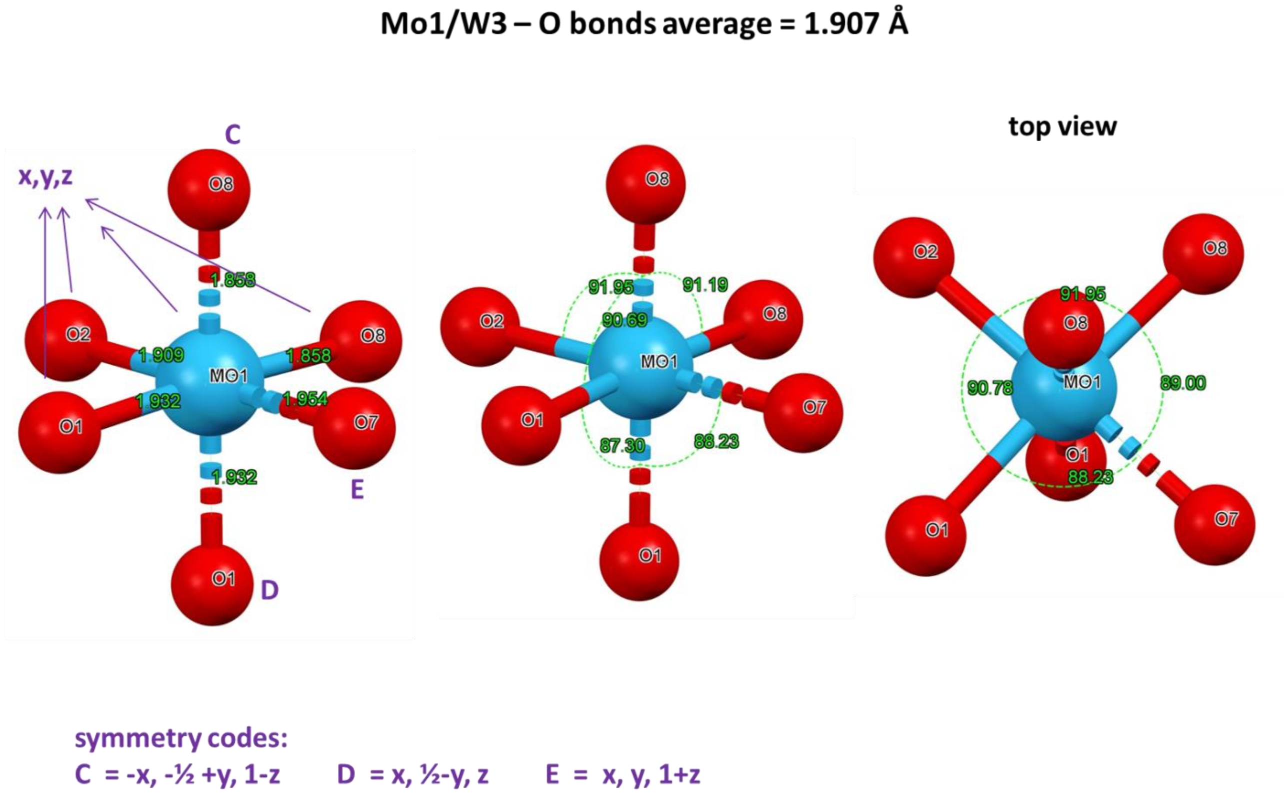

SM 33 **Figure 35**

The polyhedrons' representation of the crystal structure of compound **3**.

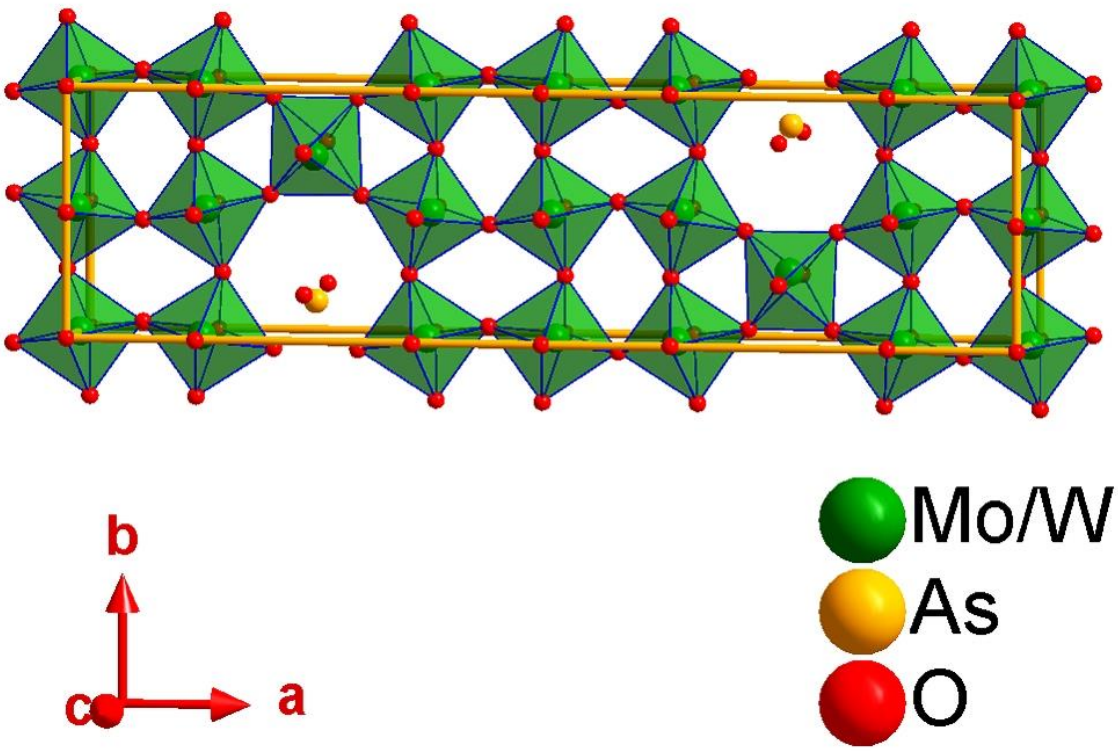

SM 34 **Figure 36**

Two orthogonal views of the GROW fragment in the structure of **3**. Corner-sharing octahedrons are clearly seen in the structure, while yellow arrows show edge-sharing As-containing tetrahedrons with MoO6 octahedrons.

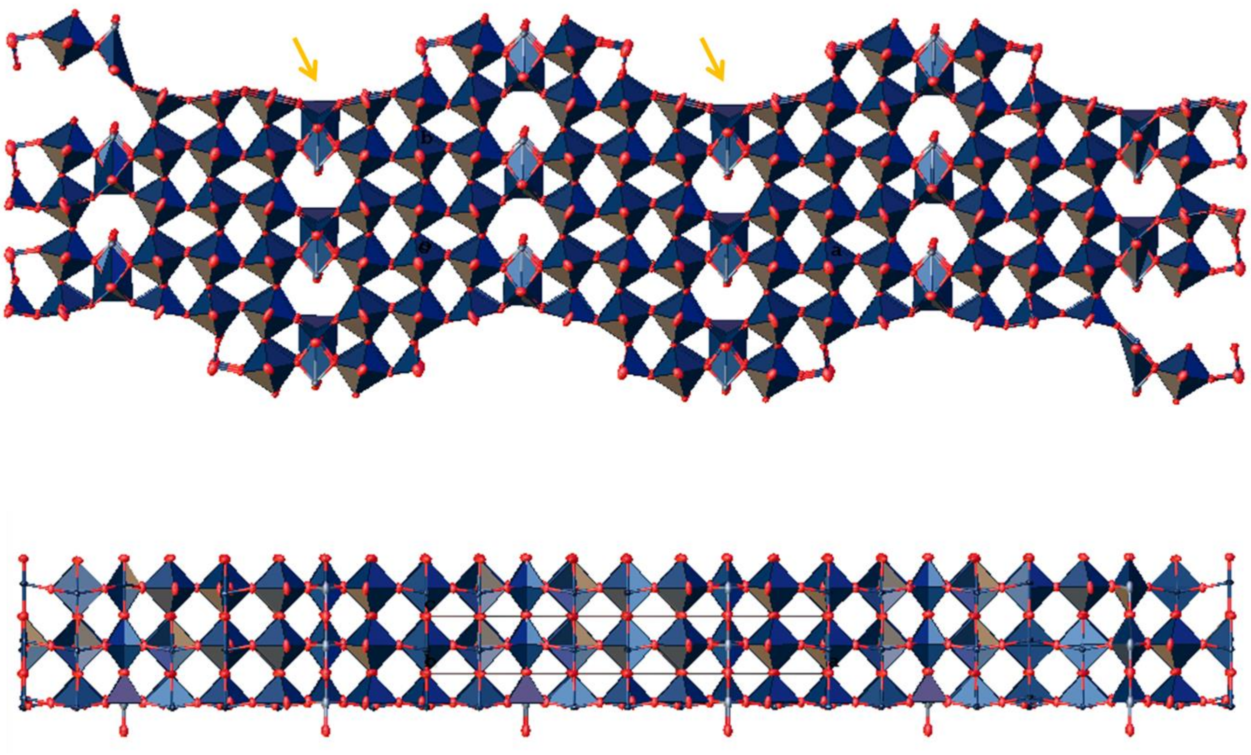

SM 35 **Figure 37**

Analysis of structure of **3**: values of principal bonds and angles at individual metal centers.

Mo1/W1 – O bonds average = 1.924 Å

top view

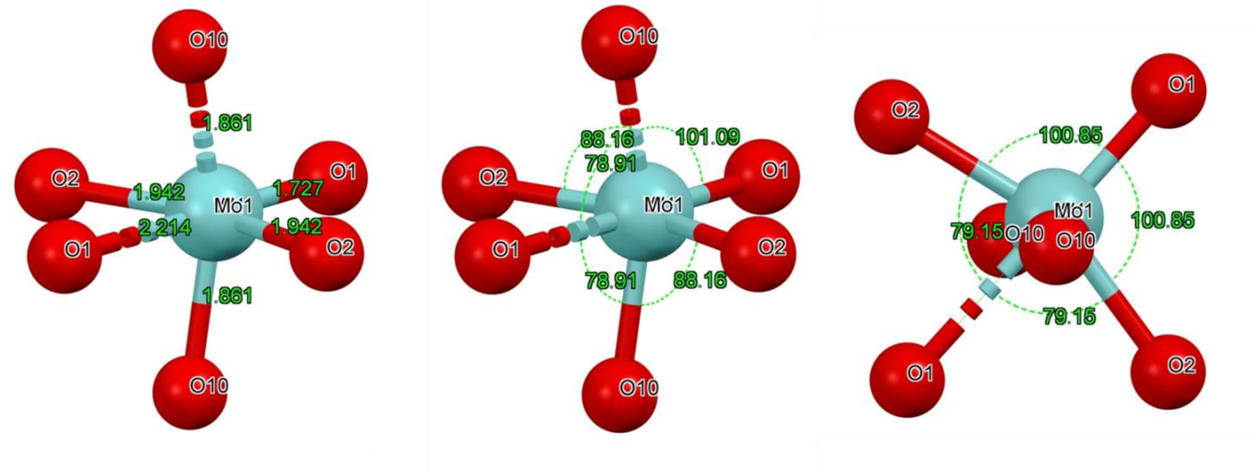

Mo2/W2 – O bonds average = 1.931 Å

top view

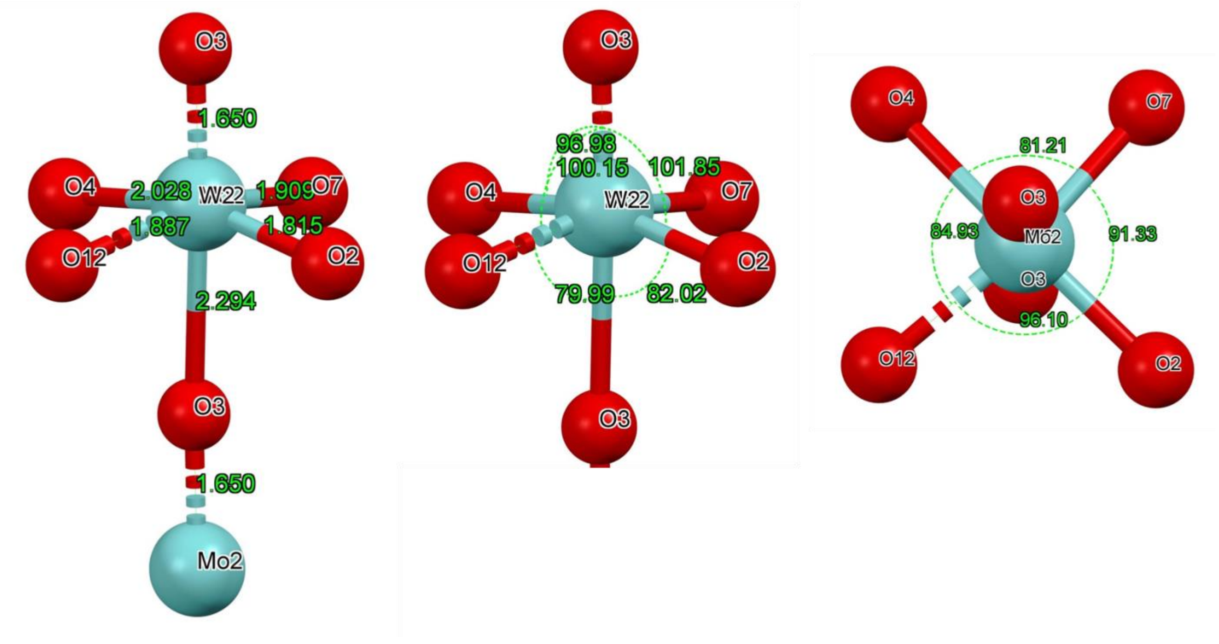

SM 36 **Figure 38**

Analysis of structure of **3**: values of principal bonds and angles at individual metal centers (continued).

Mo3/W3 – O bonds average = 1.956 Å

top view

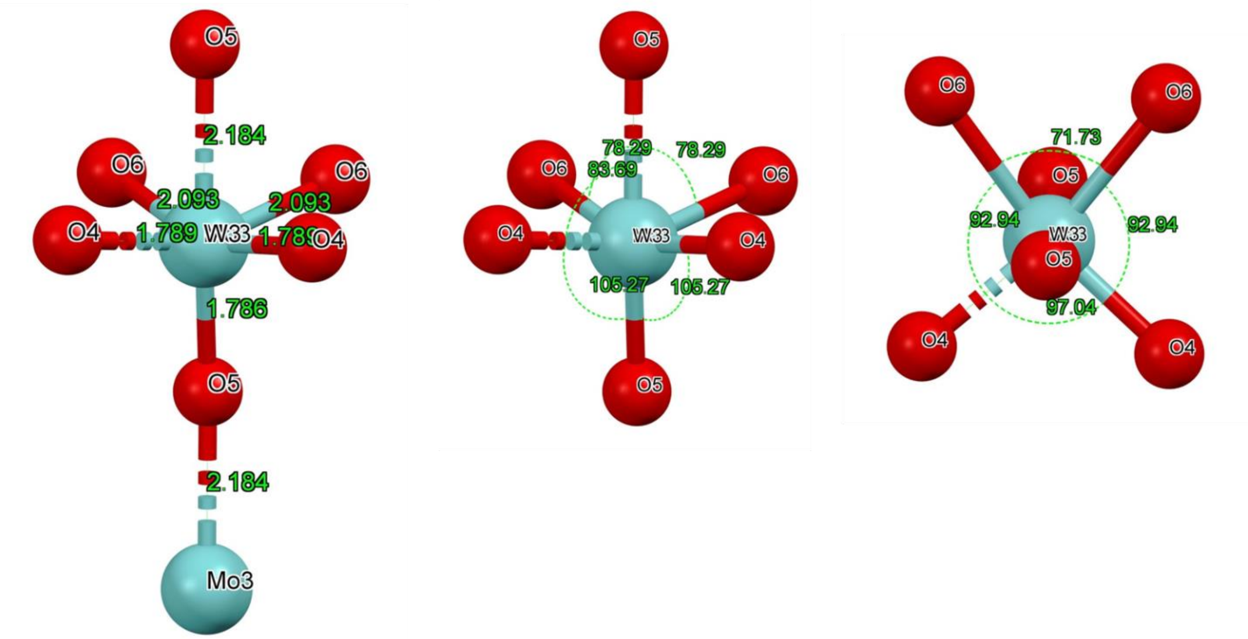

Mo4/W4– O bonds average = 1.936 Å

top view

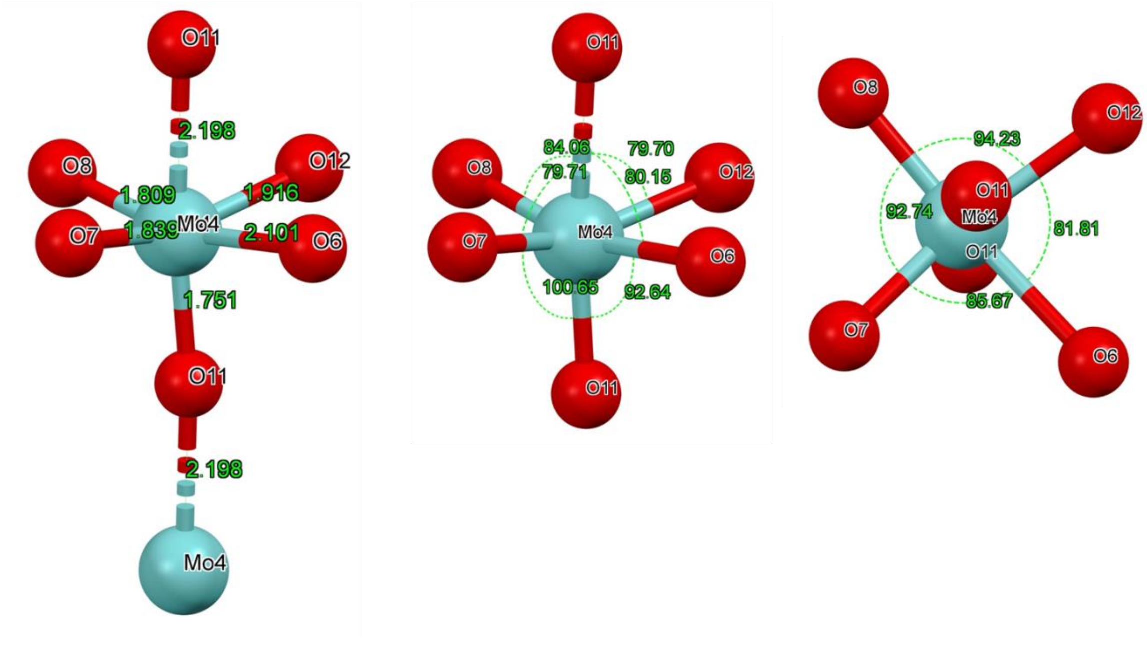

SM 37 **Figure 39**

Analysis of structure of **3**: values of principal bonds and angles at individual metal centers (continued).

Mo5/W5– O bonds average = 1.933 Å

top view

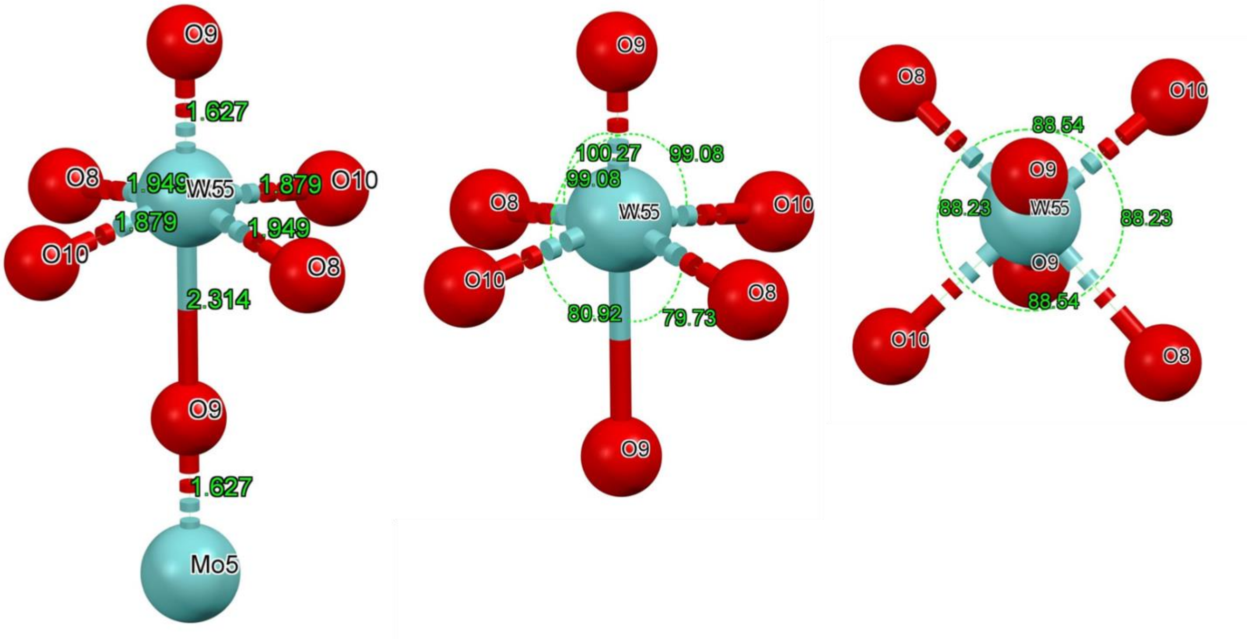

SM 38 **Figure 40**

Literature data analysis of polyhedrons distortions: selected bond lengths and valence angle in the structure of MoO_3 (monoclinic, $P2_1/n$) for comparison with same parameters in structures of **2 - 4**. Non-symmetry related metal centers Mo3 and Mo7 were chosen. A spread of Mo–O bond distances ranging from 1.777–2.276 Å, ~22% of value, while spread of O-Mo-O angles is from 77.69 to 102.51°, which represents 24.2% value (from reference [4] (see the Reference Section at the end).

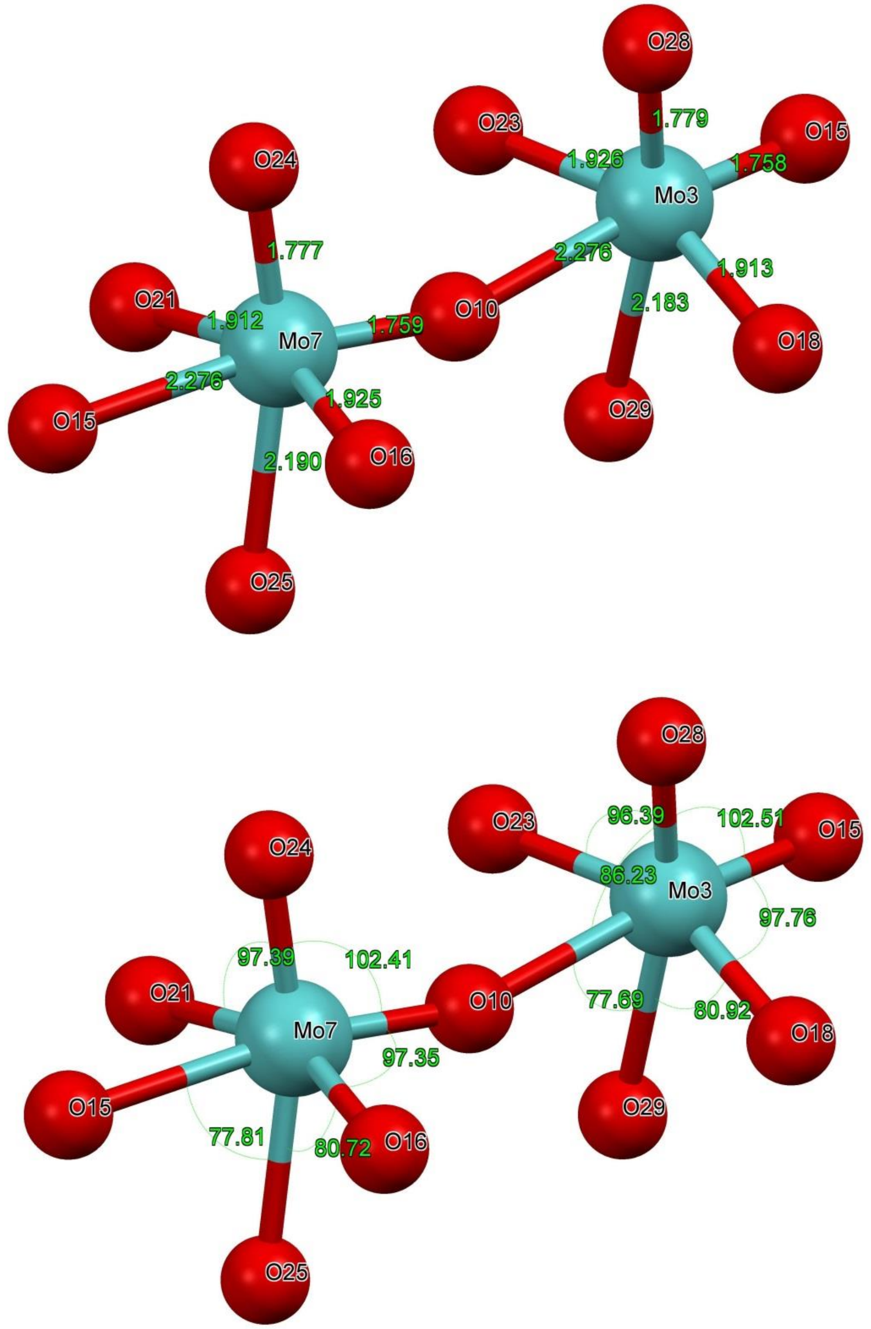

SM 39 **Figure 41**

Analysis of planarity of Metal-Oxygen net in the core of structure of **3**.

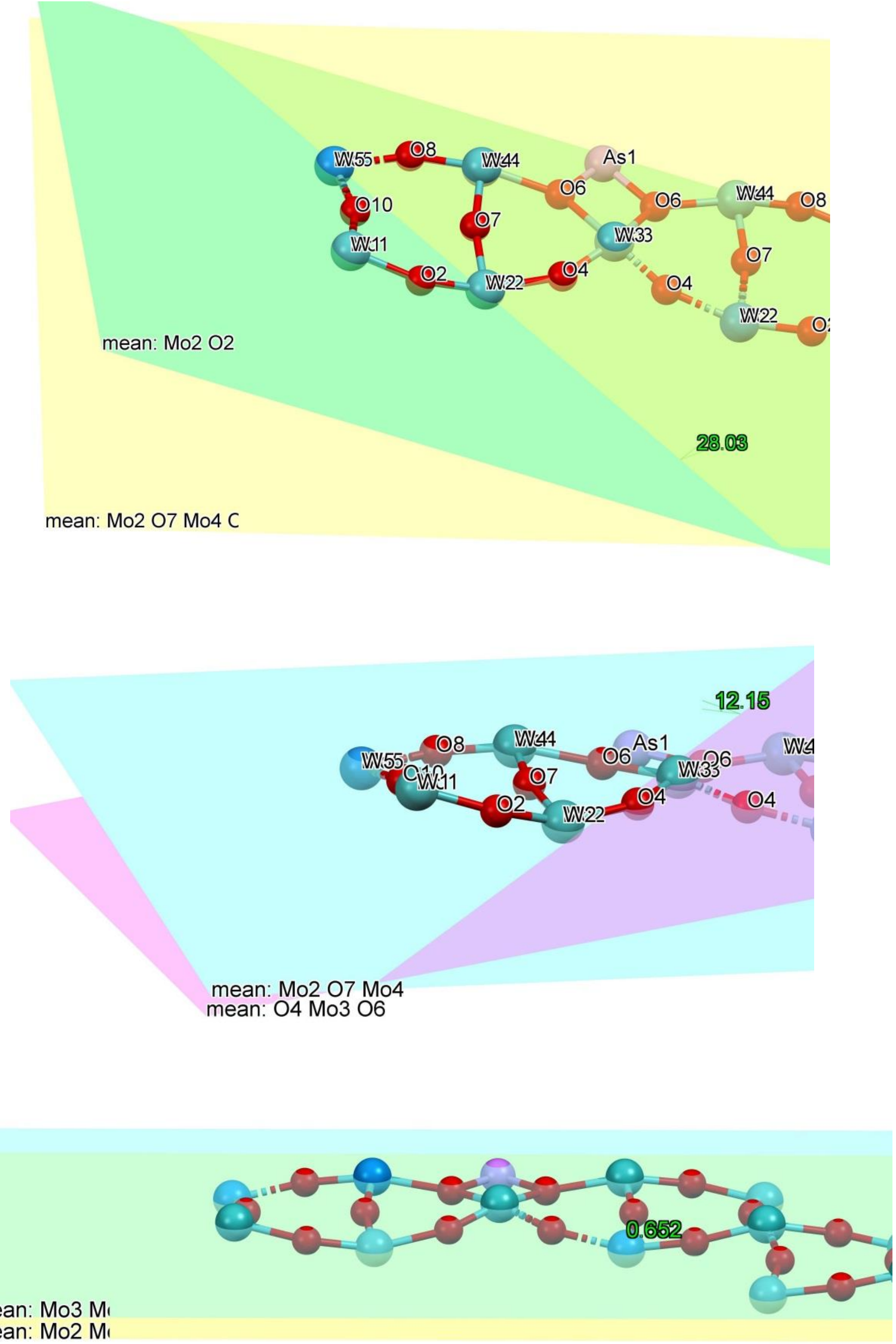

SM 40 Figure 42,43

Core of the structure of **3**: polymeric 2D net of Mo/W and O atoms: Top view (**A**) and side view (**B**).

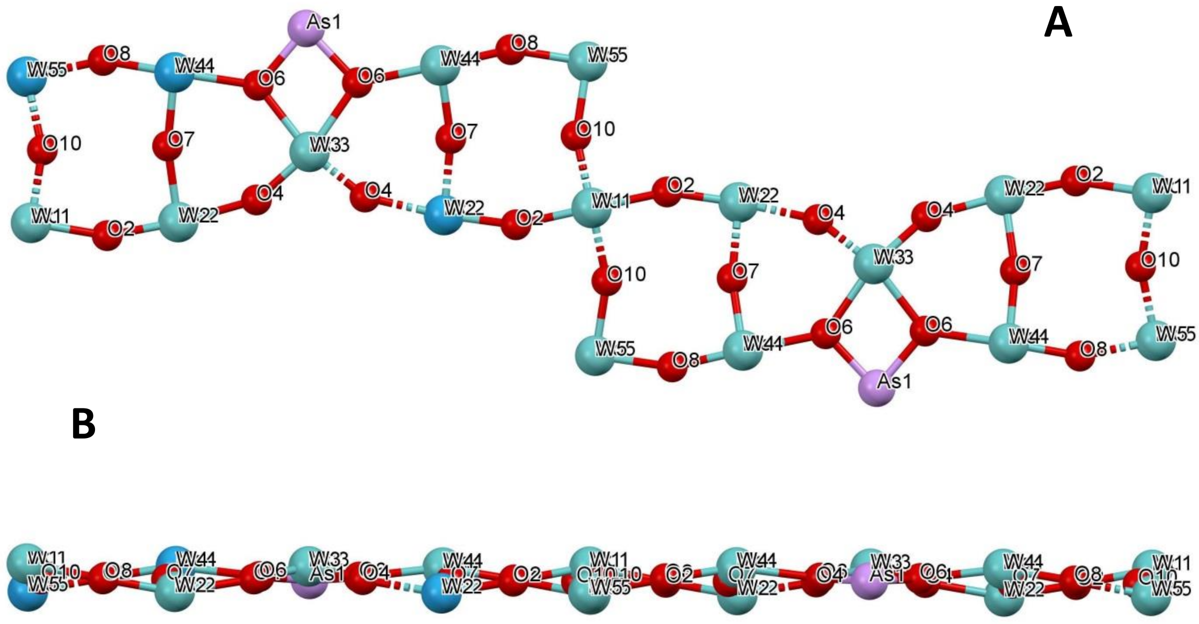

43: Details of geometry of the As1 atom. Place for the lone pair is inferred as well.

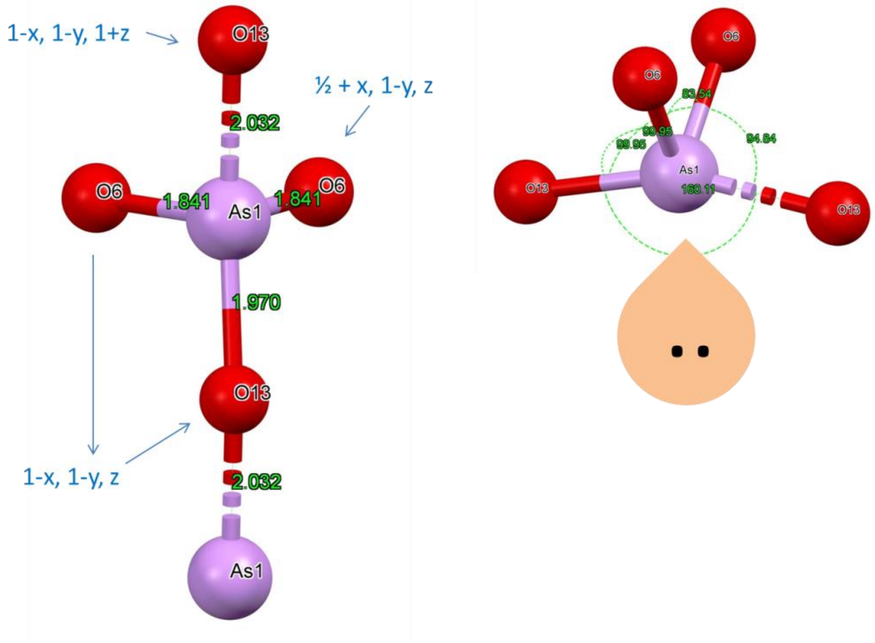

SM 41 **Figure 44**

Polyhedrons’ representation of crystal structure of compound **4**.

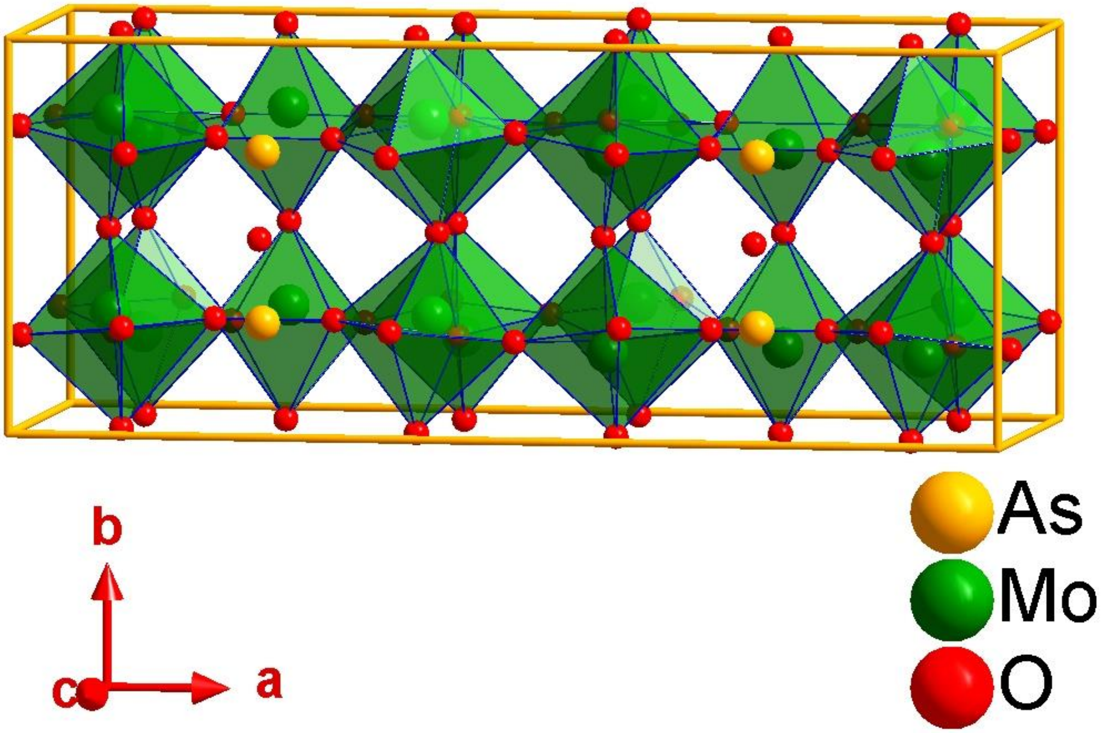

SM 42 **Figure 45**

Two orthogonal views of the GROW fragment in the structure of **4**. Only corner-sharing octahedrons are clearly seen in the structure. Arrows point out on location of As-atoms.

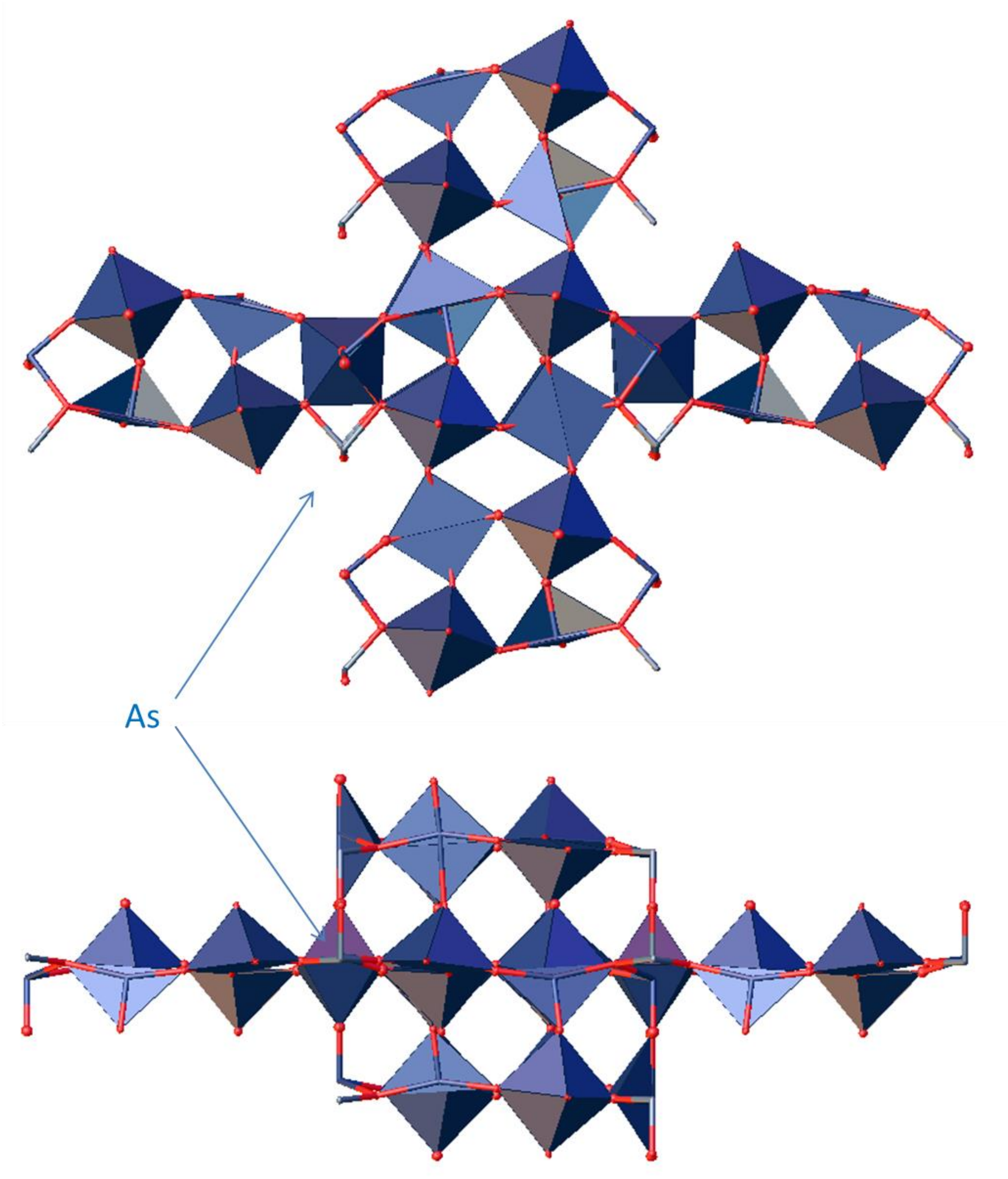

SM 43 Figure 46

Bonds lengths between metal center and oxygen in the structure of compound **4**.

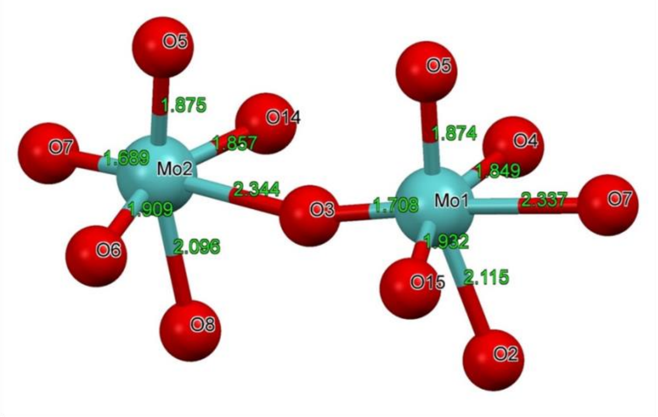

Mo2 – O = 1.961 Å average Mo1 – O = 1.969 Å average

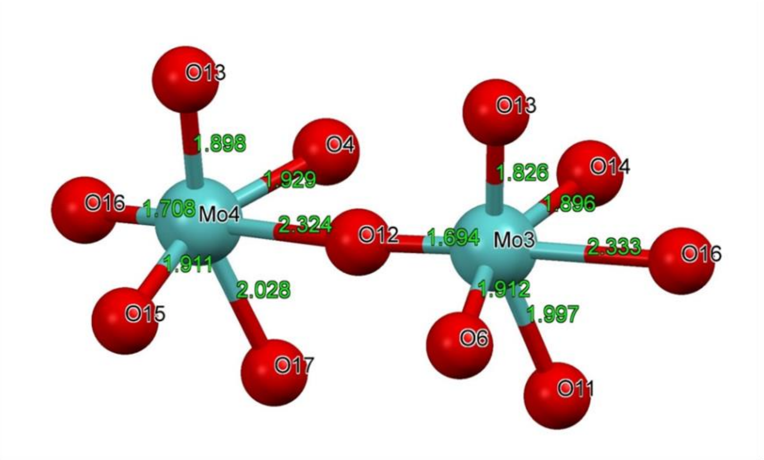

Mo4 – O = 1.965 Å average Mo3 – O = 1.944 Å average

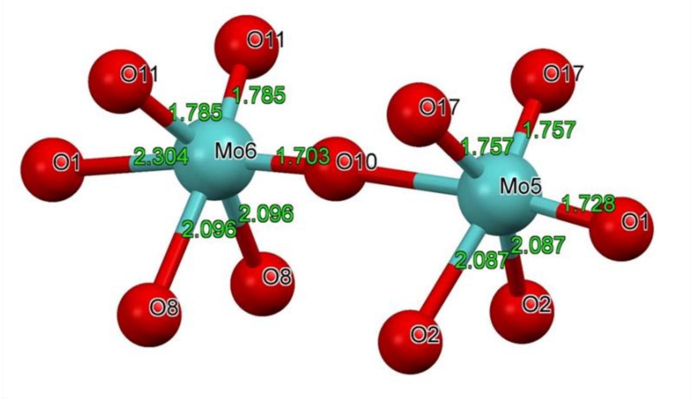

Mo6 – O = 1.962 Å average Mo5 – O = 1.952 Å average

SM 44 Figure 47

Some experimental details for single crystals electrical conductivity measurements:

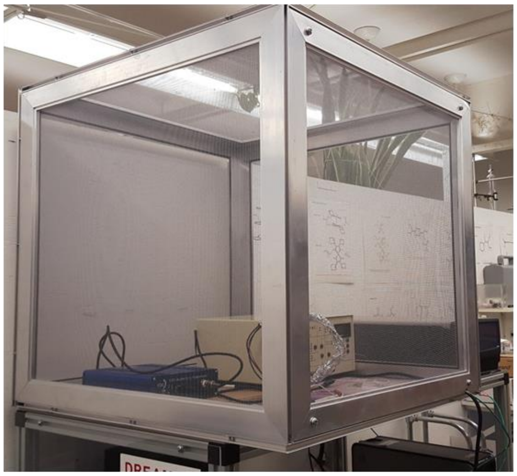

Faradaic cage

Potentiostat

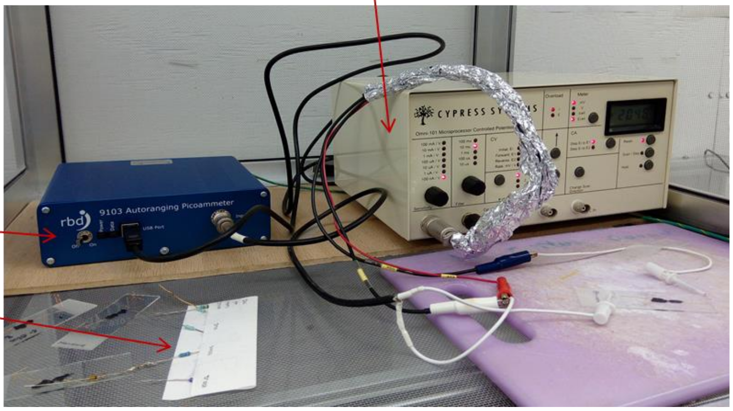

Picoammeter

Bank of standard resistors

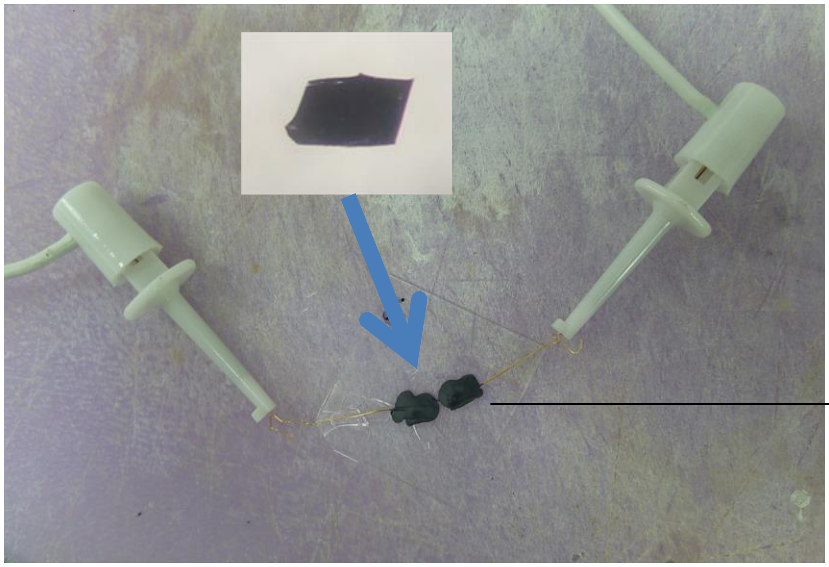

Glass cover slip with single crystal of compound **2** and graphite glue/paste with embedded golden wires.

Shown original crystal (inset) and glued specimen (below)

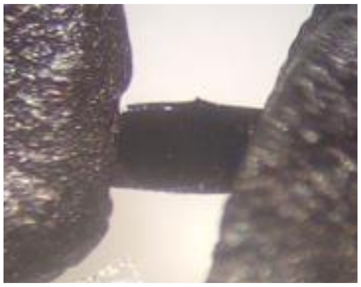

Bulk conductivity formula:

Sample's electric resistance **R** (unit Ω) is calculated from potentiostat's voltage **U** (unit: V) and observed current ***I*** (unit: A) using Ohm's law:

$$R = \frac{U}{I}$$

Electric circuit of the experiment setup:

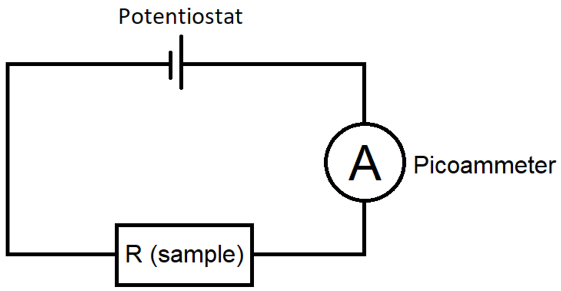

Electrical resistivity **ρ** (unit: Ωm) is calculated from sample's resistance **R**, sample's length **l** (unit: m) and cross sectional area **A** (unit m^2). Junctions covered by the carbon glue are not counted to the crystal's length. Crystals are assumed to resemble cuboids so cross-sectional area **A** is width **w** (unit: m) times the height **h** (unit: m).

$$\rho = \frac{RA}{l} = \frac{Rwh}{l}$$

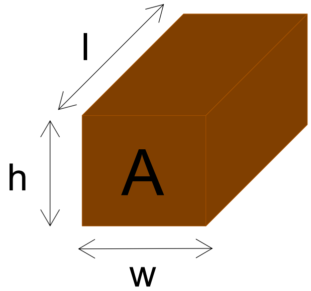

Electrical conductivity **σ** (unit: S/m) is the reciprocal of electrical resistivity **ρ**. Thus,

$$\sigma = \frac{1}{\rho} = \frac{1}{(Rwh)/l} = \frac{l}{Rwh}$$

SM 46 **Text 49**

Reference section:

1. The Materials Project. Materials Data on Nb2O5 by Materials Project. United States: N. p., 2020. Web, doi:10.17188/1276924, https://www.osti.gov/dataexplorer/biblio/dataset/1276924 (accessed on 01/24/2021)

2. Persson, K.A. Materials Data on WO_3 (SG:14) by Materials Project, doi:10.17188/1193818 Available online at: https://materialsproject.org/materials/mp-19033/ (Accessed on 27/01/2021)

3. Jain, A.; Ong, S.-P.; Hautier,G.; Chen, W.; Richards, D.W.; Dacek, S.; Cholia, S.; Gunter, D.; Skinner, D.; Ceder, G.; Persson, K.A. The Materials Project: A materials genome approach to accelerating materials innovation. *Applied Materials*. **2013**, 1, 011002; https://doi.org/10.1063/1.4812323

4. Persson, K.A. Materials Data on MoO_3 by Materials Project. Available online at: https://materialsproject.org/materials/mp-18856/#snl (Accessed on 26/01/2021)
